# Generation of allogeneic and xenogeneic functional muscle stem cells for intramuscular transplantation

**DOI:** 10.1172/JCI166998

**Published:** 2024-05-07

**Authors:** Ajda Lenardič, Seraina A. Domenig, Joel Zvick, Nicola Bundschuh, Monika Tarnowska-Sengül, Regula Furrer, Falko Noé, Christine L. Trautmann, Adhideb Ghosh, Giada Bacchin, Pjeter Gjonlleshaj, Xhem Qabrati, Evi Masschelein, Katrien De Bock, Christoph Handschin, Ori Bar-Nur

**Affiliations:** 1 Laboratory of Regenerative and Movement Biology, Department of Health Sciences and Technology, ETH Zurich, Schwerzenbach, Switzerland.; 2 Biozentrum, University of Basel, Basel, Switzerland.; 3 Functional Genomics Center Zurich, ETH Zurich and University of Zurich, Zurich, Switzerland.; 4 Laboratory of Exercise and Health, Department of Health Sciences and Technology, ETH Zurich, Schwerzenbach, Switzerland.

**Keywords:** Muscle biology, Stem cells, Movement disorders, Skeletal muscle, iPS cells

## Abstract

Satellite cells, the stem cells of skeletal muscle tissue, hold a remarkable regeneration capacity and therapeutic potential in regenerative medicine. However, low satellite cell yield from autologous or donor-derived muscles hinders the adoption of satellite cell transplantation for the treatment of muscle diseases, including Duchenne muscular dystrophy (DMD). To address this limitation, here we investigated whether satellite cells can be derived in allogeneic or xenogeneic animal hosts. First, injection of CRISPR/Cas9-corrected *Dmd^mdx^* mouse induced pluripotent stem cells (iPSCs) into mouse blastocysts carrying an ablation system of host satellite cells gave rise to intraspecies chimeras exclusively carrying iPSC-derived satellite cells. Furthermore, injection of genetically corrected DMD iPSCs into rat blastocysts resulted in the formation of interspecies rat-mouse chimeras harboring mouse satellite cells. Notably, iPSC-derived satellite cells or derivative myoblasts produced in intraspecies or interspecies chimeras restored dystrophin expression in DMD mice following intramuscular transplantation and contributed to the satellite cell pool. Collectively, this study demonstrates the feasibility of producing therapeutically competent stem cells across divergent animal species, raising the possibility of generating human muscle stem cells in large animals for regenerative medicine purposes.

## Abstract 2

Therapeutically competent mouse muscle stem cells generated in rats restore dystrophin expression in a mouse model of Duchenne muscular dystrophy.

## Graphical abstract

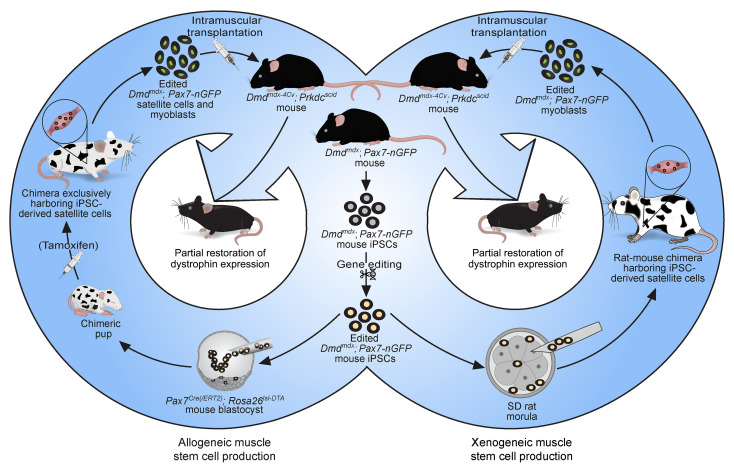

## Introduction

Muscle degeneration denotes the loss of skeletal muscle mass as a consequence of pathological conditions in the form of sarcopenia, cachexia, or muscular dystrophies (1, 2). Following muscle insult, quiescent satellite cells orchestrate a myogenic regeneration program through activation and differentiation into transit-amplifying myoblasts that differentiate into fusion-competent myocytes that merge with damaged multinucleated muscle fibers for tissue repair (3, 4). This stepwise differentiation process is characterized by upregulation of specific transcription factors, including paired box 7 (*Pax7*) in satellite cells, myogenic differentiation 1 (*Myod1*) in myoblasts, and myogenin (*Myog*) in differentiated muscle cells (3, 4). During this regeneration process, a portion of activated satellite cells returns to quiescence, reforming a new satellite cell reservoir (3, 4).

Duchenne muscular dystrophy (DMD) is the most common and currently incurable muscular dystrophy. It arises due to a mutation in the dystrophin gene, which encodes a large structural protein that connects skeletal muscle fibers to the extracellular matrix (2, 5, 6). In patients with DMD, lack of dystrophin renders muscle fibers highly susceptible to breakage due to muscle contraction forces, resulting in increased regeneration cycles by satellite cells (2). However, continuous erosion of myofibers gradually exhausts the regeneration capacity of satellite cells, resulting in muscle fiber replacement with fibrotic and adipogenic tissues over time (7). As a consequence of skeletal muscle wasting, patients with DMD become wheelchair dependent during childhood and consequently succumb to untimely death due to cardiorespiratory complications usually in the second or third decade of life (7).

A variety of therapeutic interventions are currently being explored for their capacity to restore dystrophin expression (8). Such efforts include gene therapy using overexpression of micro-dystrophin or correction of the DMD mutation by CRISPR/Cas9, typically through the use of adeno-associated viruses (AAVs) (8). While promising, these approaches still raise concerns, including AAV toxicity, genomic integration, or DNA breakage, as well as unfavorable immunological responses against repeated AAV treatment or Cas9 (9–13). Alternatively, cell-based therapies have been extensively explored for their potential to restore dystrophin expression in DMD animal models by injection of myogenic stem or progenitor cells into dystrophic muscles (14–16). Such trials aim to add healthy myonuclei to dystrophic myofibers through cell fusion for dystrophin restoration (16, 17). Early endeavors in the 1990s, utilizing healthy myoblasts to restore dystrophin expression in patients with DMD, were unsuccessful, albeit more recent trials reported better outcomes (18–20). In a different disease, myoblast transplantation has been successful in improving the condition of patients with oculopharyngeal muscular dystrophy in a phase I clinical trial (21).

Several reasons have been proposed for the unfavorable outcome of cell-based therapy in skeletal muscle tissue, including immunological rejection of transplanted cells, donor-derived cell death upon transplantation, limited engraftment only around the injection site, and more (14, 15). One notable explanation has been that myoblasts lose in vivo engraftment capabilities following extensive in vitro expansion (22). Therefore, major efforts have been directed toward finding means to augment the engraftment potential of myoblasts, or seeking additional expandable myogenic cell types that can efficiently restore dystrophin expression in vivo following intramuscular injection in DMD animal models (14–16). Several notable examples include induced pluripotent stem cell–derived (iPSC-derived) myogenic precursor cells, teratoma-derived muscle stem cells, or directly reprogrammed induced myogenic progenitor cells (23–28). However, satellite cells are still widely considered one of the most potent cell types capable of restoring dystrophin expression, since low numbers of satellite cells can efficiently engraft and regenerate muscles in vivo (22, 29–32). With respect to treating patients with DMD, harvesting sufficient numbers of satellite cells from donor-derived muscles poses a major challenge for cell-based therapy (14).

Blastocyst complementation represents a sophisticated technology that enables the creation of specific cell types, tissues, or organs from donor-derived pluripotent stem cells (PSCs) (33). To this end, PSCs such as embryonic stem cells (ESCs) or iPSCs are injected into blastocysts that carry genetic mutations that impede the formation of specific cell types or organs in animal chimeras, thereby enabling exclusive generation from injected PSCs (33). In recent years, this approach has been utilized to produce cells and organs in intraspecies mouse-mouse or pig-pig chimeras (33). Notably, this technique has been demonstrated in an interspecies manner, through the production of cell types or organs in xenogeneic animal hosts, including pancreas, bone marrow, blood vasculature, kidneys, thymi, or germ cells in mice or rats (34–42). However, generation of genetically corrected interspecies muscle stem cells in different animal species has not been reported (33). Here, we set out to combine cellular reprogramming, genome engineering, and in vivo differentiation of PSCs in mouse-mouse and rat-mouse chimeras to generate genetically corrected mouse muscle stem cells that can be exploited to treat DMD by restoring dystrophin expression in dystrophic mice.

## Results

### Substantial production of ESC-derived satellite cells in intraspecies mouse chimeras.

**Figure 1:**
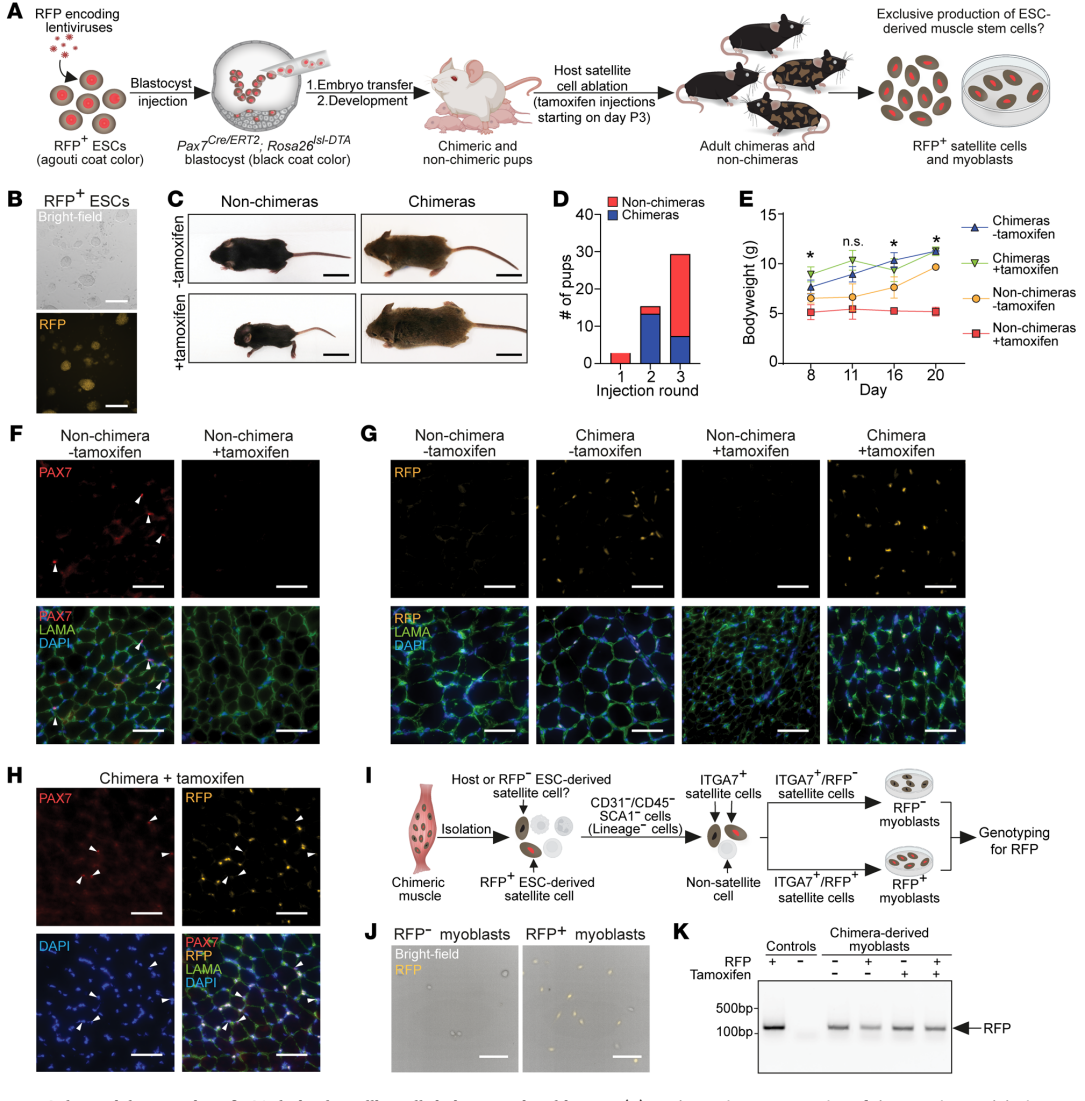
Substantial generation of ESC-derived satellite cells in intraspecies chimeras. (**A**) A schematic representation of the experimental design. RFP, red fluorescent protein; ESCs, embryonic stem cells. (**B**) Representative images of ESCs transduced with lentiviruses encoding RFP. Scale bars: 200 μm. (**C**) Photos showing chimeric and non-chimeric mice on day 17. Chimerism is evidenced by agouti coat color. Scale bars: 1 cm. (**D**) A graph showing the derivation of chimeras per injection round. (**E**) A graph depicting weight changes during postnatal growth of the specified mouse groups. Asterisks indicate a significant difference (*P* < 0.05) in body weight of the “non-chimeras + tamoxifen” group compared with all other groups. *n* ≥ 3 animals, data are presented as mean ± SD. Statistical analysis was performed using a 2-way ANOVA. *P ≤ 0.05. (**F**) Representative immunostaining images for the indicated markers in muscle cross sections of the specified animals on day 17. Scale bars: 50 μm. (**G**) Immunostaining images for the specified markers in skeletal muscle cross sections on day 17 of the indicated animals. Look-up tables (LUTs) for the GFP and DAPI channels were individually adjusted. Scale bars: 50 μm. (**H**) Immunostaining for PAX7 in muscle cross sections of a chimera on day 17 following host satellite cell ablation. White arrowheads indicate PAX7^+^ satellite cells. Scale bars: 50 μm. (**I**) A schematic illustrating the strategy to assess RFP lentiviral transgene silencing in ESC-derived satellite cells. (**J**) Representative images of ITGA7^+^ FACS-purified myoblasts isolated from chimera muscles following satellite cell ablation. Scale bars: 100 μm. (**K**) PCR for RFP in the indicated myoblast lines and conditions. Note that the RFP transgene is present even in myoblast lines that do not express RFP.

We commenced our study by setting out to explore whether ESCs can exclusively produce satellite cells in intraspecies chimeras generated using mouse blastocysts carrying *Pax7^Cre/ERT2^* and *Rosa26^loxSTOPlox–Diphtheria^ ^toxin^ ^A^* (*Rosa26^lsl-DTA^*) homozygous alleles (43, 44). As satellite cells uniquely express *Pax7* in skeletal muscles (45), this system ensures specific ablation of host-derived satellite cells following tamoxifen injection, and can potentially provide a vacant niche receptive for ESC-derived satellite cell colonization in skeletal muscles of chimeras (Figure 1A). To address this question, we employed lentivirus-transduced red fluorescent protein–positive KH2-ESCs (RFP^+^ ESCs), which have been previously reported to contribute robustly to mouse chimerism (Figure 1, A and B) (36, 46). Of note, prior to blastocyst injections, RFP^+^ ESCs were cultured for 5 days in enhanced culture medium to increase chimeric contribution (47). Altogether, we performed 3 blastocyst injection rounds that gave rise to 28 out of 58 (48%) chimeric offspring, based on genotyping for the RFP allele and presence of agouti coat color emanating from RFP^+^ ESCs (Figure 1, C and D, and Supplemental Figure 1A; supplemental material available online with this article; https://doi.org/10.1172/JCI166998DS1). Furthermore, the mice carried the *Rosa26^lsl-DTA^* allele as expected (Supplemental Figure 1A). Next, we sought to assess whether we can exploit the genetic system to ablate host satellite cells in newborn pups, aiming to create a vacant niche receptive for reconstitution with ESC-derived satellite cells during postnatal growth. To this end, we performed tamoxifen injections in 3-day-old chimeric and non-chimeric pups for 3 consecutive days. This early developmental time point was chosen because it is characterized by rapid muscle growth associated with a high proliferation rate of endogenous PAX7^+^ satellite cells (48). Over a course of 3 weeks after birth, we observed no increase in body weight in tamoxifen-injected non-chimeric *Pax7^Cre/ERT2^*; *Rosa26^lsl-DTA^* animals, whereas the non-injected non-chimeric animals gained weight gradually (Figure 1, D and E). Notably, intraspecies *Pax7^Cre/ERT2^*; *Rosa26^lsl-DTA^*/RFP^+^ ESC chimeras showed a gradual body weight increase, even when subjected to tamoxifen injections on postnatal days 3–5, suggesting a rescue by the contribution of injected ESCs (Figure 1, D and E).

To confirm satellite cell ablation in mice, we harvested leg muscles from a non-chimeric *Pax7^Cre/ERT2^*; *Rosa26^lsl-DTA^* mouse subjected to tamoxifen injections as well as a non-injected control animal. We solely detected PAX7-expressing satellite cells in non–tamoxifen-injected muscle sections, although not in muscles of an injected mouse (Figure 1F). Next, we observed that RFP^+^ ESCs contributed extensively to skeletal muscle tissue in chimeras, as muscle sections exhibited prominent RFP expression in resident muscle cells, independent of host satellite cell ablation (Figure 1G). We then assessed whether all PAX7^+^ satellite cells expressed the RFP reporter in these muscle sections. Unexpectedly, we detected PAX7^+^ satellite cells that were RFP negative, suggesting that either host satellite cells persisted in muscles following tamoxifen injections, or that transgene silencing occurred in ESC-derived satellite cells (Figure 1H). To assess which hypothesis is correct, we FACS-purified RFP-negative or -positive CD45^–^CD31^–^SCA1^–^ITGA7^+^ satellite cells from muscles of chimeras subjected to host satellite cell ablation (Figure 1I and Supplemental Figure 1, B–D) (49). Surprisingly, we detected both RFP^+^ and RFP^–^ satellite cell populations following satellite cell ablation and we were further able to generate both RFP^+^ and RFP^–^ myoblast lines from chimeric muscles (Figure 1, I and J). Importantly, PCR analysis for RFP revealed that both the RFP^+^ and RFP^–^ cell populations contained the RFP transgene, indicating that lentiviral vector silencing may have occurred in ESC-derived satellite cells (Figure 1K). Collectively, in this first preliminary trial, we established a system that enables host satellite cell ablation in intraspecies chimeras and successfully generated satellite cells and myoblasts from donor-derived ESCs. However, lentiviral transgene silencing may have occurred in ESC-derived satellite cells, raising a need for an alternative transgenic labeling system that allows distinguishing between host- and donor-derived satellite cells.

### Exclusive generation of genetically corrected DMD iPSC-derived satellite cells in chimeras.

**Figure 2:**
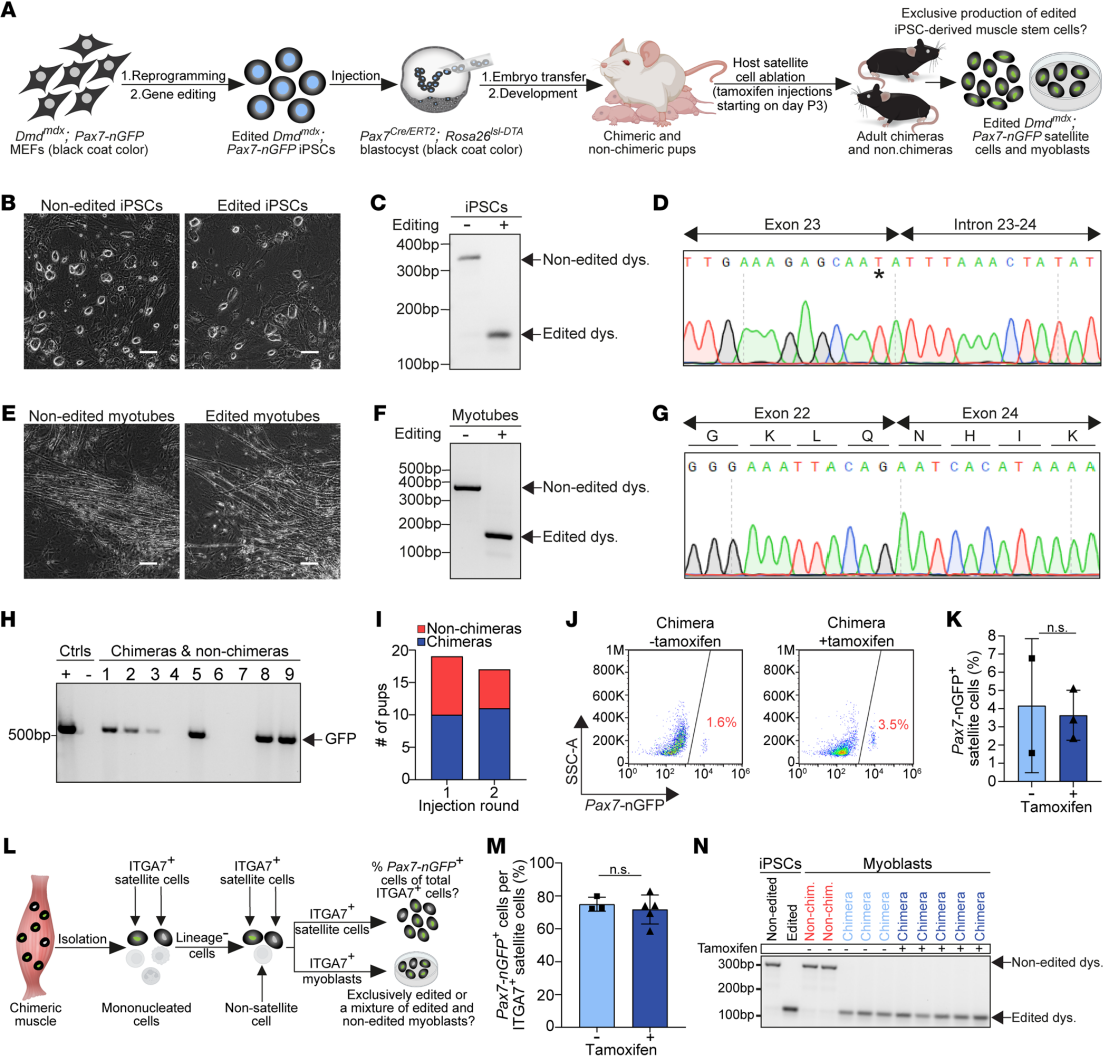
Exclusive generation of edited iPSC–derived muscle stem cells in intraspecies chimeras. (**A**) A schematic overview of the experimental plan. MEFs, mouse embryonic fibroblasts. (**B**) Representative images of the specified cell lines. Scale bars: 100 μm. LUTs were individually adjusted. (**C**) PCR products for dystrophin, amplified from the DNA of non-edited (–) and edited (+) *Dmd^mdx^;* *Pax7-nGFP* iPSCs. (**D**) DNA sequence of the edited dystrophin PCR product lacking a splice donor site. A black asterisk specifies the mdx mutation. (**E**) Representative images of non-edited and edited *Dmd^mdx^; Pax7-nGFP* iPSC–derived myotubes. Scale bars: 100 μm. LUTs were individually adjusted. (**F**) PCR for dystrophin in cDNA isolated from non-edited and edited *Dmd^mdx^; Pax7-nGFP* iPSC–derived myogenic cultures. (**G**) Sanger sequence of an edited dystrophin band shown in **F**, revealing successful exon skipping and reframing of dystrophin at the cDNA level. (**H**) Representative genotyping for the *Pax7-nGFP* allele in non-chimeric and chimeric pups. (**I**) A graph showing chimera numbers based on *Pax7-nGFP* allele genotyping. (**J**) FACS analysis of *Pax7*-nGFP expression in the indicated animals and conditions. (**K**) A graph showing quantification of the percentage of *Pax7*-nGFP^+^ cells in muscles derived from chimeras with or without tamoxifen treatment. *n* = 2–3 animals, data are presented as mean ± SD. Statistical analysis was performed using a Student’s 2-tailed *t* test. (**L**) A schematic representation outlining the strategy to determine the percentage of iPSC-derived satellite cells within the overall ITGA7^+^ (host + donor) satellite cell population of intraspecies chimeras. (**M**) A graph illustrating the percentage of iPSC-derived satellite cells, identified by the *Pax7-nGFP* reporter, out of the total ITGA7^+^ satellite cell population in chimeras. *n* = 3 animals for the non–tamoxifen-injected control, *n* = 5 animals for the tamoxifen-treated group. Data are presented as mean ± SD. Statistical analysis was performed using a Student’s 2-tailed *t* test. (**N**) PCR for dystrophin using DNA of ITGA7^+^ satellite cell–derived expanded myoblasts of the specified animals and conditions. Note that all chimera-derived myoblasts showed only an edited dystrophin band.

Given the encouraging results involving production of ESC-derived satellite cells in intraspecies chimeras, we next sought to evaluate whether a similar approach may enable exclusive production of therapeutically competent and gene-edited satellite cells and myoblasts from the well-established *Dmd^mdx^* mouse model (50). Specifically, we set out to explore whether we can derive and genetically correct *Dmd^mdx^* iPSCs that carry a *Pax7–nuclear* *GFP* (*Pax7-nGFP*) satellite cell-specific genetic reporter (51). We then aimed to utilize corrected *Dmd^mdx^*; *Pax7-nGFP* iPSCs to exclusively generate functional satellite cells from iPSCs in intraspecies chimeras following host satellite cell ablation (Figure 2A).

As the first step, we crossed homozygous *Dmd^mdx^* female mice with homozygous *Pax7-nGFP* males and derived mouse embryonic fibroblast (MEF) lines. Since the dystrophin gene is located on the X chromosome, all male MEF lines inherited the *Dmd^mdx^* mutation from the females and were heterozygous for the *Pax7-nGFP* allele. Reprogramming to pluripotency was performed using a polycistronic *STEMCCA* cassette together with small molecule treatment (Supplemental Figure 2A) (52, 53). Following manual picking, selection, and propagation of iPSC clones, we were able to establish *Dmd^mdx^*; *Pax7-nGFP* iPSCs that expressed well-known pluripotency markers (Supplemental Figure 2, A–C).

Next, we set out to correct the dystrophin mutation in exon 23 of *Dmd^mdx^*; *Pax7-nGFP* iPSCs by employing a previously described CRISPR/Cas9 exon-skipping-based strategy that results in a restored reading frame (Supplemental Figure 2, D and E) (54). To this end, we engineered and utilized a single plasmid that encodes Cas9, guide RNAs, and a puromycin selection cassette (Supplemental Figure 2D). Transfection and antibiotic selection led to the generation of edited *Dmd^mdx^*; *Pax7-nGFP* iPSC clones (Figure 2B). We confirmed successful editing of dystrophin in one of these clones at the DNA level by PCR and Sanger sequencing (Figure 2, C and D). To further validate whether *Dmd^mdx^*; *Pax7-nGFP* iPSCs were successfully edited, we employed an established in vitro–directed differentiation protocol of PSCs into myotubes (23, 55). Within 3 weeks, this effort led to the generation of contractile myotubes from gene-edited *Dmd^mdx^*; *Pax7-nGFP* iPSCs, demonstrating successful reframing of dystrophin at the mRNA level (Figure 2, E–G). Furthermore, we detected by immunostaining dystrophin^+^ myotubes solely in WT ESCs and gene-edited *Dmd^mdx^*; *Pax7-nGFP* iPSCs subjected to the differentiation protocol, but not in unedited *Dmd^mdx^*; *Pax7-nGFP* iPSC–derived myotubes (Supplemental Figure 2F).

Based on these results, we proceeded to inject karyotypically normal (*n* = 40) gene-edited *Dmd^mdx^*; *Pax7-nGFP* iPSCs into *Pax7^Cre/ERT2^*; *Rosa26^lsl-DTA^* blastocysts, producing 36 pups (Figure 2, A and B, and Supplemental Figure 2G). As both the iPSCs and host blastocysts harbored genes that encode black coat color, we employed genotyping for the *Pax7-nGFP* transgene to assess for chimerism, revealing that 21 out of 36 (58%) of the offspring were chimeric (Figure 2, H and I). We then injected chimeras with tamoxifen on postnatal days 3–5 and harvested skeletal muscles from injected and non-injected chimeras at 5 or more weeks of age, aiming to assess the number of *Pax7*-nGFP*^+^* satellite cells in muscles with and without host satellite cell ablation (Figure 2A). Remarkably, we detected *Pax7*-nGFP*^+^* satellite cells in chimeras following satellite cell ablation; however, we also observed an appreciable number of *Pax7*-nGFP^+^ satellite cells in non-injected chimeras, suggesting that cell ablation was not critical for derivation of donor iPSC–derived satellite cells in chimeras (Figure 2, J and K). FACS-purified satellite cells were then isolated from both tamoxifen-injected and non-injected chimeras, giving rise to *Pax7*-nGFP^+^ myoblast lines (Supplemental Figure 2, H and I). Importantly, we confirmed that all examined *Pax7*-nGFP^+^ myoblast lines solely carried a correctly edited dystrophin gene (Supplemental Figure 2J).

The observation that comparable numbers of edited *Dmd^mdx^*; *Pax7-nGFP* satellite cells were generated in tamoxifen-injected and non-injected chimeras prompted us to explore the extent to which PAX7^+^ cell ablation may enhance iPSC contribution to the satellite cell niche. To this end, we analyzed additional chimeras that have been treated with and without tamoxifen injections, and purified satellite cells by FACS from their skeletal muscles using established surface markers (CD45^–^CD31^–^SCA1^–^ITGA7^+^) (Figure 2L and Supplemental Figure 2, K and L) (49). In this way, we determined that most ITGA7^+^ satellite cells were GFP^+^, both with and without tamoxifen administration (Figure 2, L and M, and Supplemental Figure 2, K–M). We then plated CD45^–^CD31^–^SCA1^–^ITGA7^+^ satellite cells and observed that nearly all myoblasts were GFP^+^ (Supplemental Figure 2N). Importantly, all examined ITGA7^+^ satellite cell–derived myoblast lines obtained from chimeras contained only the genetically corrected dystrophin allele, corroborating that indeed all satellite cells were derived from gene-edited *Dmd^mdx^*; *Pax7-nGFP* iPSCs (Figure 2N).

Next, we performed molecular characterization of chimera-derived edited *Dmd^mdx^*; *Pax7-nGFP* myoblasts, documenting nearly homogeneous GFP expression in these lines (Supplemental Figure 3, A and B). Bulk RNA-seq analysis of FACS-purified myoblasts revealed elevated expression of myoblast-related myogenic markers, similar to FACS-purified myoblasts that were derived from *Pax7-nGFP* mice, and much higher than in *Pax7-nGFP* MEFs (Supplemental Figure 3, C–E) (56). We then differentiated edited *Dmd^mdx^*; *Pax7-nGFP* myoblasts into myotubes and observed downregulation of the *Pax7-nGFP* reporter expression (Supplemental Figure 3F). PCR and cDNA sequencing of the myotubes revealed faithful correction of the dystrophin mutation (Supplemental Figure 3, G and H). Notably, we observed dystrophin protein expression only in *Pax7-nGFP* and edited *Dmd^mdx^*; *Pax7-nGFP* myoblast–derived myotubes, but not in unedited *Dmd^mdx^*; *Pax7-nGFP* myoblast–derived myotubes, albeit all expressed myosin heavy chain (MYHC) (Supplemental Figure 3I). Collectively, these results imply that gene-edited iPSCs are the cell of origin of satellite cells isolated from muscles of intraspecies chimeras. Surprisingly, efficient satellite cell derivation was also observed in the absence of host satellite cell ablation.

### An alternative system enabling exclusive satellite cell generation in intraspecies chimeras.

**Figure 3:**
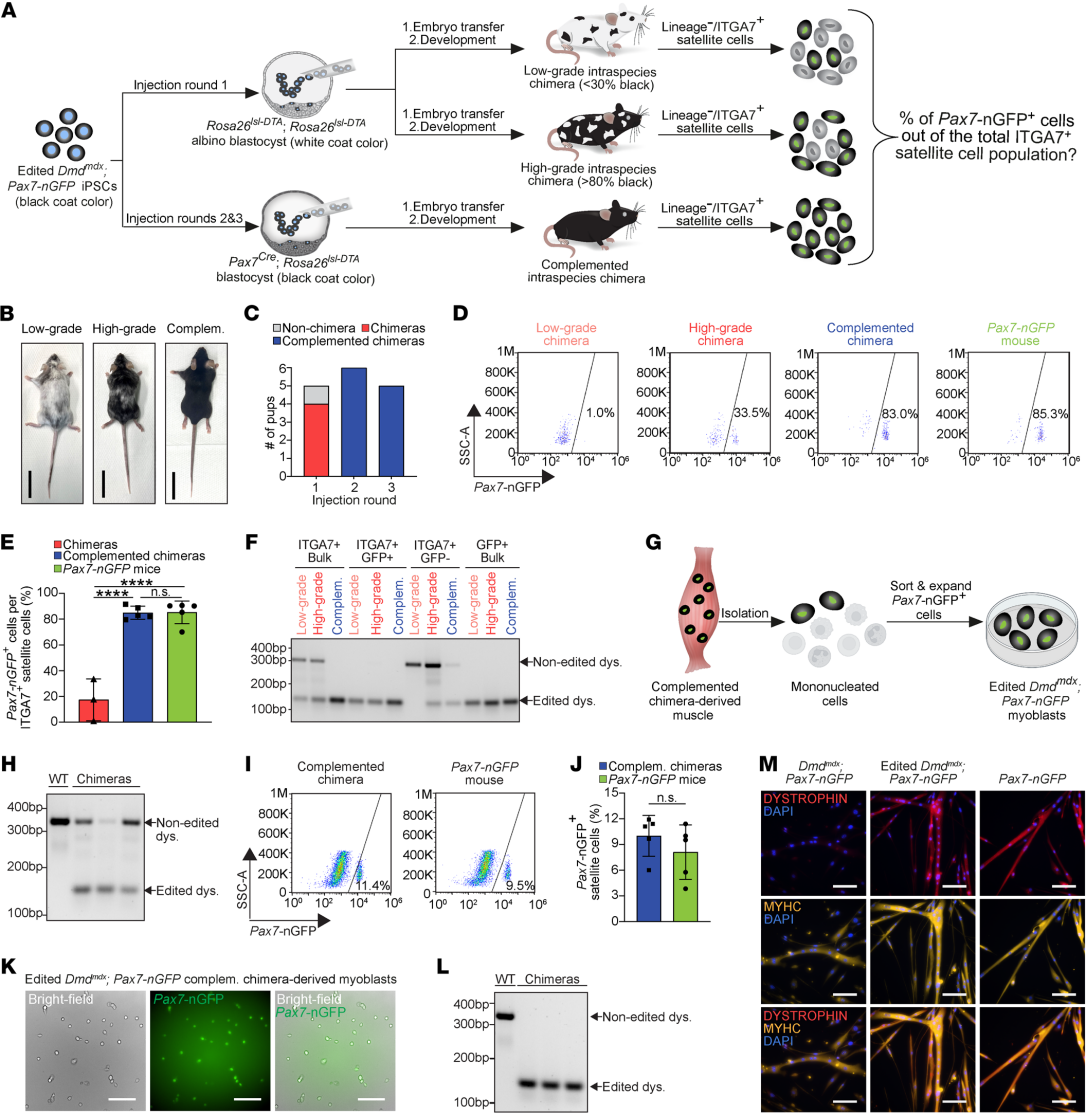
Constitutive PAX7^+^ cell ablation enables exclusive iPSC-derived satellite cell production in chimeras. (**A**) Schematic representation of the experimental design. (**B**) Representative images of a low- and a high-grade chimera, as well as a complemented chimera. Black coat color indicates iPSC chimeric contribution in low- and high-grade chimeras. Scale bars: 3 cm. (**C**) A graph showing chimera numbers based on coat color or *Pax7-nGFP* allele genotyping. (**D**) Representative FACS plots displaying the percentage of *Pax7*-nGFP^+^ cells within the ITGA7^+^ satellite cell population of the indicated animals. (**E**) A graph showing the quantification of the FACS plot shown in **D** for a larger group of analyzed mice. *n* = 3 animals for non-complemented chimeras and *n* = 5 animals for complemented chimeras as well as *Pax7-nGFP* control mice. Data are presented as mean ± SD. Statistical analysis was performed using an ordinary 1-way ANOVA with Tukey’s multiple-comparison test. ****P ≤ 0.0001. (**F**) PCR for dystrophin using DNA extracted from the specified cell populations and animals. (**G**) Schematic representation of the isolation and expansion of myoblasts from the muscles of complemented chimeras. (**H**) PCR for dystrophin in total muscles of the specified mice prior to satellite cell isolation. (**I**) Representative FACS plots showing *Pax7*-nGFP expression in muscles of the indicated animals. (**J**) A graph showing quantification of the analysis shown in **I**. *n* = 5 animals for each group. Data are presented as mean ± SD. Statistical analysis was performed using a Student’s 2-tailed *t* test. (**K**) Representative images of chimera-derived edited *Dmd^mdx^; Pax7*-nGFP*^+^* myoblasts. Scale bars: 100 μm. (**L**) PCR for dystrophin using DNA extracted from FACS-purified *Dmd^mdx^; Pax7*-nGFP*^+^* myoblasts. Note the presence of only an edited band. (**M**) Immunostaining for the indicated markers in myoblast-derived myotubes from the specified cell lines. Scale bars: 100 μm.

The unexpected results thus far pointed toward exclusive satellite cell generation with and without host PAX7^+^ cell ablation during postnatal growth in chimeras. We hypothesize that, in this instance, the gene-edited iPSCs contributed robustly to muscles and the satellite cell pool in chimeras, rendering postnatal host satellite cell ablation dispensable for iPSC-derived muscle stem cell colonization. However, discerning low- or high-grade chimerism based on coat color was challenging, as the iPSCs and host blastocysts gave rise to mice with dark coat color, such that visually distinguishing between them was unfeasible (Figure 2A). Alternatively, leakiness of the Cre enzyme from the *Pax7* promoter in the absence of tamoxifen administration may have led to the ablation of host satellite cells in chimeras. To address these experimental challenges, we opted to assess the contribution of gene-edited *Dmd^mdx^*; *Pax7-nGFP* iPSCs in 2 additional chimera models: (i) albino *Rosa26^lsl-DTA^* blastocysts (i.e., no Cre expression), and (ii) constitutive *Pax7^Cre^*; *Rosa26^lsl-DTA^* blastocysts, wherein PAX7-expressing cells are ablated at the embryonic stage, and do not require tamoxifen injections to induce Cre expression (Figure 3A) (57). This effort has led to the production of 4 *Dmd^mdx^*; *Pax7-nGFP*/albino *Rosa26^lsl-DTA^* low- and high-grade chimeras and 1 non-chimeric mouse (Figure 3, B and C). In addition, in 2 injection rounds of iPSCs into *Pax7^Cre^*; *Rosa26^lsl-DTA^* blastocysts, we generated 11 *Dmd^mdx^*; *Pax7-nGFP*/*Pax7^Cre^*; *Rosa26^lsl-DTA^* chimeras that all carried the *Pax7-nGFP* allele; however, we did not obtain non-chimeric animals (Figure 3, B and C, and Supplemental Figure 4A). This remarkably suggests that blastocyst complementation with iPSCs was critical for embryo survival. To corroborate this hypothesis, we transferred 63 non-injected *Pax7^Cre^*; *Rosa26^lsl-DTA^* blastocysts into foster female mice and did not observe live births, indicating that ablation of PAX7-expressing cells during embryonic development was detrimental to survival (Supplemental Figure 4, B and C).

Next, we harvested and analyzed skeletal muscles from *Dmd^mdx^*; *Pax7-nGFP*/*Rosa26^lsl-DTA^* and *Dmd^mdx^*; *Pax7-nGFP*/*Pax7^Cre^*; *Rosa26^lsl-DTA^* chimeras. Similar to prior trials (Figure 2), we aimed to evaluate the number of iPSC-derived *Pax7*-nGFP^+^ cells out of the total CD45^–^CD31^–^SCA1^–^ITGA7^+^ satellite cells in muscles (Supplemental Figure 2, K and L). In control *Pax7-nGFP* mice, approximately 85% of the CD45^–^CD31^–^SCA1^–^ITGA7^+^ cells were also *Pax7*-nGFP^+^, suggesting that most, but not all, ITGA7^+^ cells express the *Pax7-nGFP* reporter (Figure 3, D and E). Notably, this percentage was similar in complemented chimeras and significantly lower in non-complemented chimeras, which exhibited a variation in *Pax7-nGFP* expression in accordance with the degree of coat color chimerism (Figure 3, D and E). Consistent with this result, PCR analysis for dystrophin revealed a prominent presence of the corrected allele in muscle resident cells that have been purified by FACS from complemented chimeras, and substantially less in non-complemented chimeras (Figure 3F). We then set out to purify *Pax7*-nGFP^+^ satellite cells from complemented chimeras by FACS and confirmed that the percentage of *Pax7*-nGFP^+^ cells in their skeletal muscles was similar to that of *Pax7-nGFP* mice (Figure 3, G–J). We further confirmed that these *Pax7*-nGFP^+^ myoblasts maintained reporter expression in vitro and carried only the gene-edited dystrophin allele (Figure 3, K and L). Importantly, myotubes derived from these *Pax7*-nGFP^+^ myoblasts downregulated reporter expression and were positive for dystrophin, thus unequivocally demonstrating their genetic correction (Figure 3M and Supplemental Figure 4D). In summary, using an alternative genetic system and through blastocyst complementation with iPSCs, we demonstrate overcoming fetal lethality associated with PAX7^+^ cell ablation during embryonic development. These findings have enabled exclusive generation of iPSC-derived satellite cells in intraspecies chimeras that could give rise to myoblasts and derivative myotubes that expressed dystrophin.

### Dystrophin restoration in DMD mice using intraspecies chimera–derived satellite cells and myoblasts.

**Figure 4:**
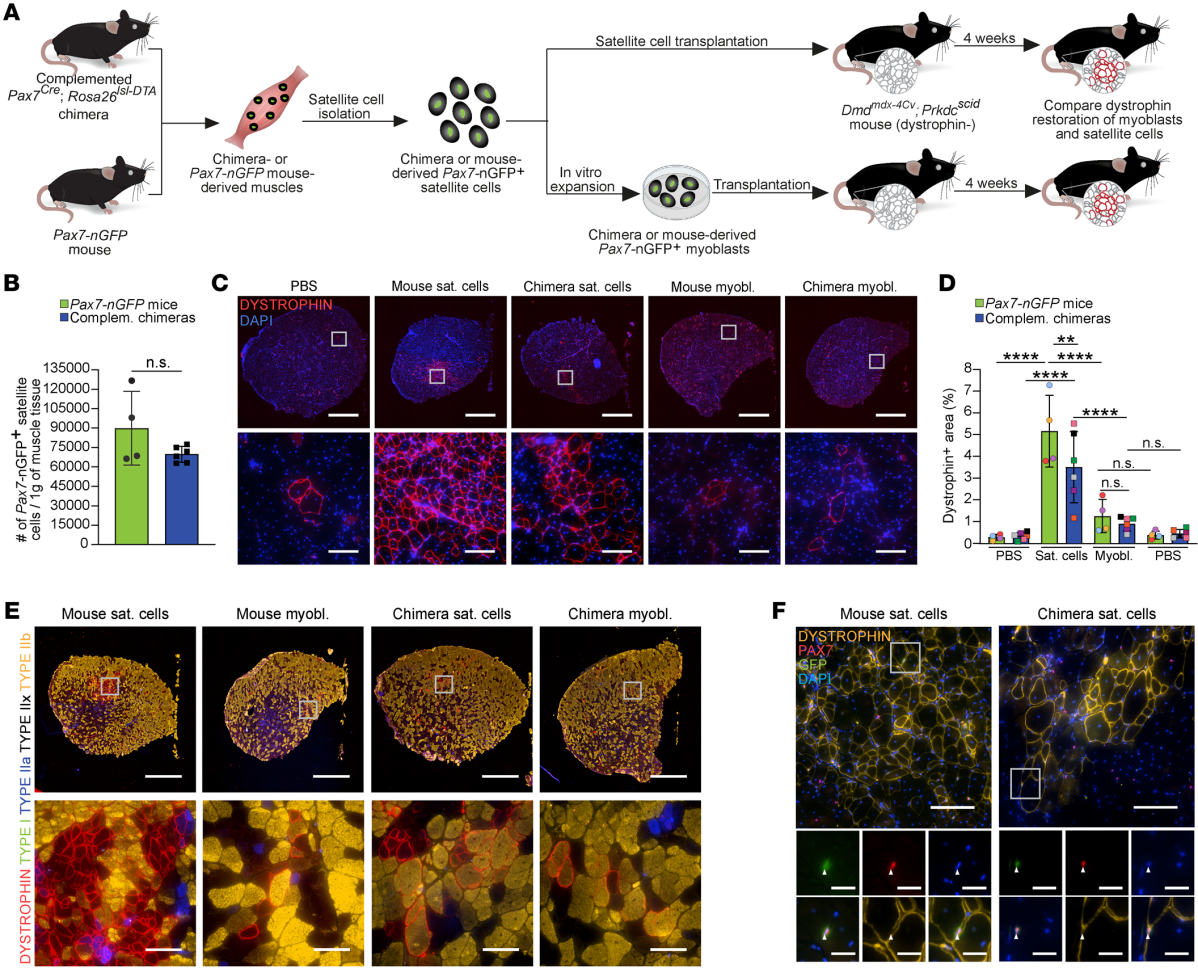
Chimera-derived muscle stem cells restore dystrophin expression in DMD mice. (**A**) Schematic representation depicting the strategy for intramuscular transplantation. A similar number of satellite cells or expanded myoblasts were transplanted from the same mouse. (**B**) A graph showing the number of *Pax7*-nGFP^+^ cells obtained from the specified animals. *n* = 4 animals for *Pax7-nGFP* mice and *n* = 6 animals for chimeras. Data are presented as mean ± SD. Statistical analysis was performed using a Student’s 2-tailed *t* test. (**C**) Representative immunostaining images of tibialis anterior (TA) muscle cross section of *Dmd^mdx-4Cv^*; *Prkdc^scid^* mice stained for dystrophin 4 weeks after transplantation with the indicated cell lines. Scale bars: 1 mm (top panel) and 100 μm (bottom panel). Sat. cells, satellite cells; myobl., myoblasts. (**D**) Quantification of the transplantation trial shown in **C**. *n* = 5 animals for *Pax7-nGFP* mice and *n* = 6 animals for chimeras. Each dot represents 1 recipient, with colors specifying cells derived from the same donor. The number of transplanted cells from each donor is shown in Supplemental Figure 5A. Data are presented as mean ± SD. Statistical analysis was performed using a 2-way ANOVA. **P *≤* 0.01; ****P ≤ 0.0001. (**E**) Representative immunostaining images for dystrophin and fiber typing in TA muscle cross sections of *Dmd^mdx-4Cv^*; *Prkdc^scid^* mice 4 weeks after transplantation with the specified mouse-derived cell lines. Scale bars: 1 mm (top) and 100 μm (bottom). (**F**) Representative images of TA muscle cross section from *Dmd^mdx-4Cv^*; *Prkdc^scid^* mice immunostained for the indicated markers 4 weeks after transplantation with the specified cell lines. Arrowheads point to colocalization of PAX7 expression and the *Pax7-nGFP* reporter in rare cells. Scale bars: 100 μm (top) and 25 μm (bottom). LUTs were individually adjusted.

For cell-based therapy, the capacity of muscle stem cells to fuse and repair damaged muscle fibers in addition to contributing cells to the satellite cell reservoir is of key importance (16). As such, we sought to evaluate whether intraspecies iPSC–derived muscle stem cells can restore dystrophin expression in DMD mice following intramuscular cell transplantation (Figure 4A). To this end, we explored whether edited satellite cells and derivative myoblasts, generated in complemented *Dmd^mdx^*; *Pax7-nGFP*/*Pax7^Cre^*; *Rosa26^lsl-DTA^* chimeras, can efficiently restore dystrophin expression in cardiotoxin-preinjured (CTX-preinjured) dystrophic tibialis anterior (TA) muscles of immunodeficient *Dmd^mdx-4Cv^*; *Prkdc^scid^* mice (Figure 4A) (58, 59). As the first step, we confirmed that complemented chimeras harbored on average the same number of satellite cells in their skeletal muscles as *Pax7-nGFP* mice (Figure 4B). We then transplanted freshly isolated *Pax7*-nGFP^+^ satellite cells and in vitro*–*expanded *Pax7*-nGFP^+^ myoblasts from chimeras or control *Pax7-nGFP* mice into preinjured TA muscles of *Dmd^mdx-4Cv^*; *Prkdc^scid^* mice (Supplemental Figure 5A). From each donor mouse, we ensured that we transplanted approximately the same number of satellite cells or expanded myoblasts for direct comparison between the 2 cell types (Supplemental Figure 5A). Four weeks after transplantation, we harvested and analyzed the muscles, documenting clusters of dystrophin^+^ myofibers around the injection site, which were absent in PBS-injected control animals, aside from rare revertant fibers (Figure 4C). We mostly observed a significant increase in dystrophin restoration when using satellite cells compared with myoblasts, in accordance with prior studies (Figure 4, C and D, and Supplemental Figure 5, B–D) (22, 29). Of note, we did not record a very different number of dystrophin-restored myofibers when using *Pax7-nGFP* satellite cells and myoblasts produced in either control *Pax7-nGFP* mice or intraspecies chimeras (Figure 4, C and D, and Supplemental Figure 5, B–D). Notably, even when using satellite cells, we documented only up to 7% dystrophin restoration in an entire muscle section, highlighting the known challenges associated with the limited migration of transplanted cells in skeletal muscle cell therapy (14–16). Next, we aimed to determine the muscle fiber type (i.e., type I, IIa, IIx, and IIb) in dystrophin-restored myofibers. This analysis revealed that all fiber types were observed in dystrophin-restored myofibers across the transplantation experiments, yet we predominantly documented dystrophin restoration in association with type IIa, IIx, and IIb myofibers (Figure 4E and Supplemental Figure 5, E and F). Finally, we wished to assess whether engrafted intraspecies-derived satellite cells can populate the satellite cell niche in dystrophic muscles. Capitalizing on the *Pax7-*nGFP reporter expression, we detected PAX7^+^ cells that expressed GFP and were in association with dystrophin-restored myofibers, demonstrating that these cells were derived from transplanted satellite cells (Figure 4F).

### Mouse satellite cells produced in interspecies rat-mouse chimeras.

**Figure 5:**
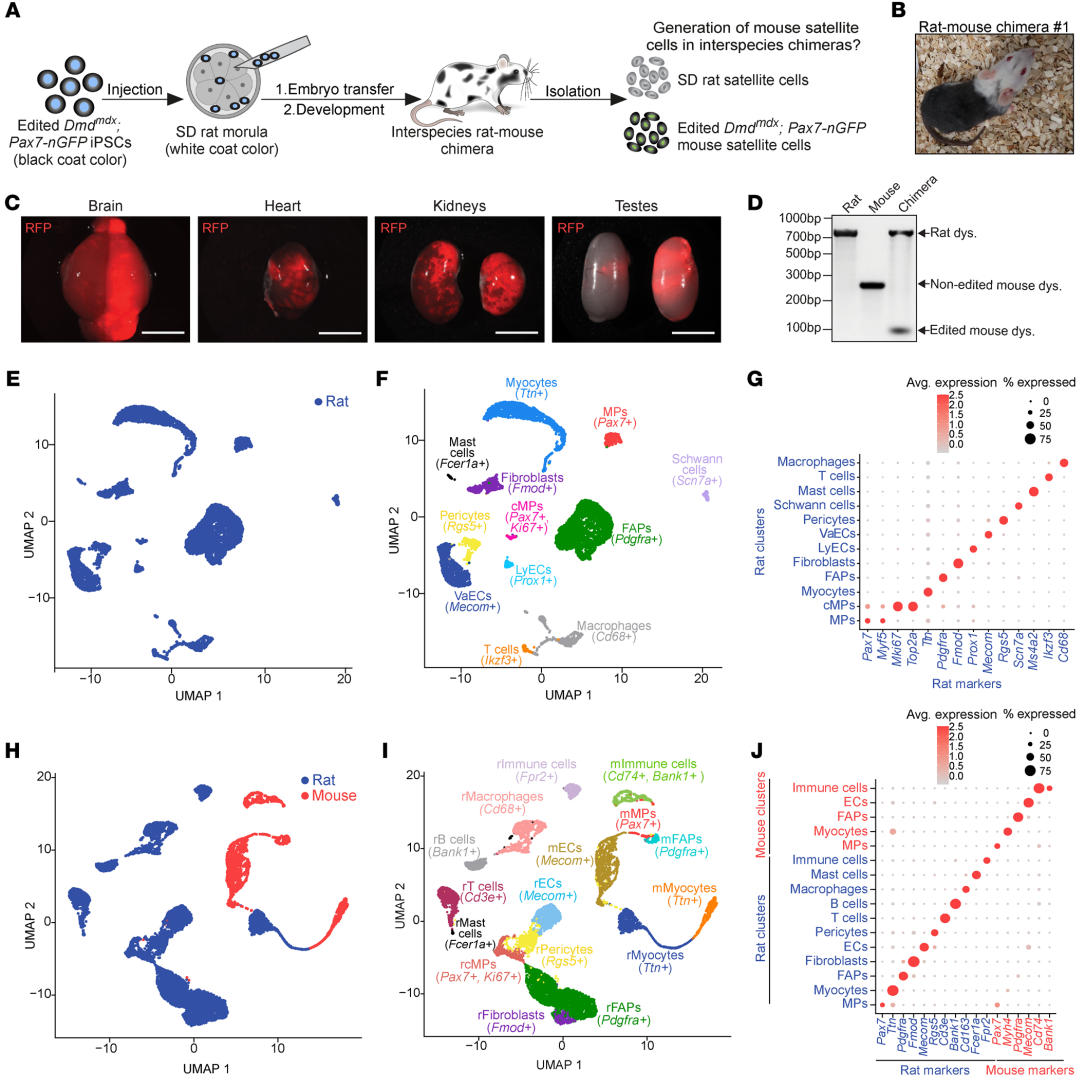
Generation of iPSC-derived mouse satellite cells in rat-mouse chimeras. (**A**) A schematic overview of the experimental design. (**B**) A photo of rat-mouse chimera no. 1. (**C**) Images of RFP expression in organs derived from rat-mouse chimera no. 1. Scale bars: 1 cm. (**D**) PCR for rat and mouse dystrophin using DNA from digested muscles of the indicated animals. (**E**) Uniform manifold approximation and projection (UMAP) based on scRNA-seq of all cells in SD rat–derived muscles colored by species. (**F**) UMAP of all cells in SD rat–derived muscle colored by different cell types. MPs, myogenic progenitors; cMPs, cycling myogenic progenitors; VaECs, vascular endothelial cells; LyECs, lymphatic endothelial cells; FAPs, fibro-adipogenic progenitors. (**G**) Dot plot for individual gene expression in various SD rat–derived cell populations shown in **F**. (**H**) UMAP of all cells in rat-mouse chimera–derived muscles colored by species. (**I**) UMAP of all cells in rat-mouse chimera–derived muscles colored by different cell types. ECs, endothelial cells. The letters “r” and “m” indicate rat and mouse, respectively. (**J**) Dot plot for individual gene expression in the rat-mouse chimera cell populations shown in **I**.

The ability to generate genetically corrected satellite cells in intraspecies chimeras, even without host satellite cell ablation (Figure 2), prompted us to investigate whether mouse muscle stem cells could be generated in another animal host. To address this objective, we chose rats as recipient hosts, since xenogeneic cells and organs were previously produced in rat-mouse chimeras (33, 41). We chose to inject edited *Dmd^mdx^*; *Pax7-nGFP* iPSCs into Sprague-Dawley (SD) rat morulae to assess, after embryonic development, the generation of mouse satellite cells in adult rat-mouse chimeras (Figure 5A). Following injection of 8–12 iPSCs, the embryos were transferred to the oviducts of foster rats and brought to term. Collectively, this effort resulted in the formation of 7 rat-mouse chimeras out of 25 pups (28%), as judged by patches of black coat color, in contrast with the white coat color of SD rats (Figure 5, A and B). To assess for iPSC contribution to internal organs, we prelabeled 1 iPSC clone with lentiviruses encoding RFP prior to morulae injections (Supplemental Figure 6A). This effort culminated in the generation of a rat-mouse chimera that demonstrated extensive mouse iPSC contributions to multiple internal organs, as evidenced by RFP reporter expression (Figure 5, B and C, and Supplemental Figure 6B).

Next, skeletal muscle cells isolated from a rat-mouse chimera were genotyped for dystrophin, unraveling the presence of rat dystrophin, but strikingly also the edited mouse dystrophin allele due to the contribution of *Dmd^mdx^*; *Pax7-nGFP* iPSCs (Figure 5D). However, we could not assess whether these were in muscle stem cells, fibers, or other resident cells of the tissue. To address this question, we performed single-cell RNA-seq (scRNA-seq) analysis of skeletal muscles isolated from 1 of the 7 chimeras and a rat control. Prior to this analysis, we assembled a combined mouse and rat reference genome and mapped the reads as previously reported (36). The rat muscles consisted of 12 cell populations, including fibro-adipogenic progenitors (FAPs), immune and endothelial cells, in addition to myocytes and muscle stem cells, which were annotated based on established markers (Figure 5, E–G, and Supplemental Figure 6C). In the muscles of an interspecies chimera, we could distinguish between rat and mouse cells using read alignment, albeit a small number of mRNA transcripts aligned with both species due to sequence similarity (Figure 5, H–J, and Supplemental Figure 6D). We could readily annotate rat resident muscle cells, which represented the majority of cells within a chimera’s muscles (7997 cells) in comparison with mouse cells (1956 cells) (Figure 5, H–J). Of note, within the mouse cell populations, we detected cells that expressed satellite cell markers (*Pax7*^+^, *Myf5*^+^) and myocyte markers (*Neb*^+^, *Tcap*^+^) (Figure 5, I and J, Supplemental Figure 6E), demonstrating that the mouse iPSCs contributed to these cell populations in a rat-mouse chimera. These findings interestingly imply host immune tolerance against mouse antigens, likely stemming from exposure to both mouse and rat cells during immune system maturation in chimeras.

**Figure 6:**
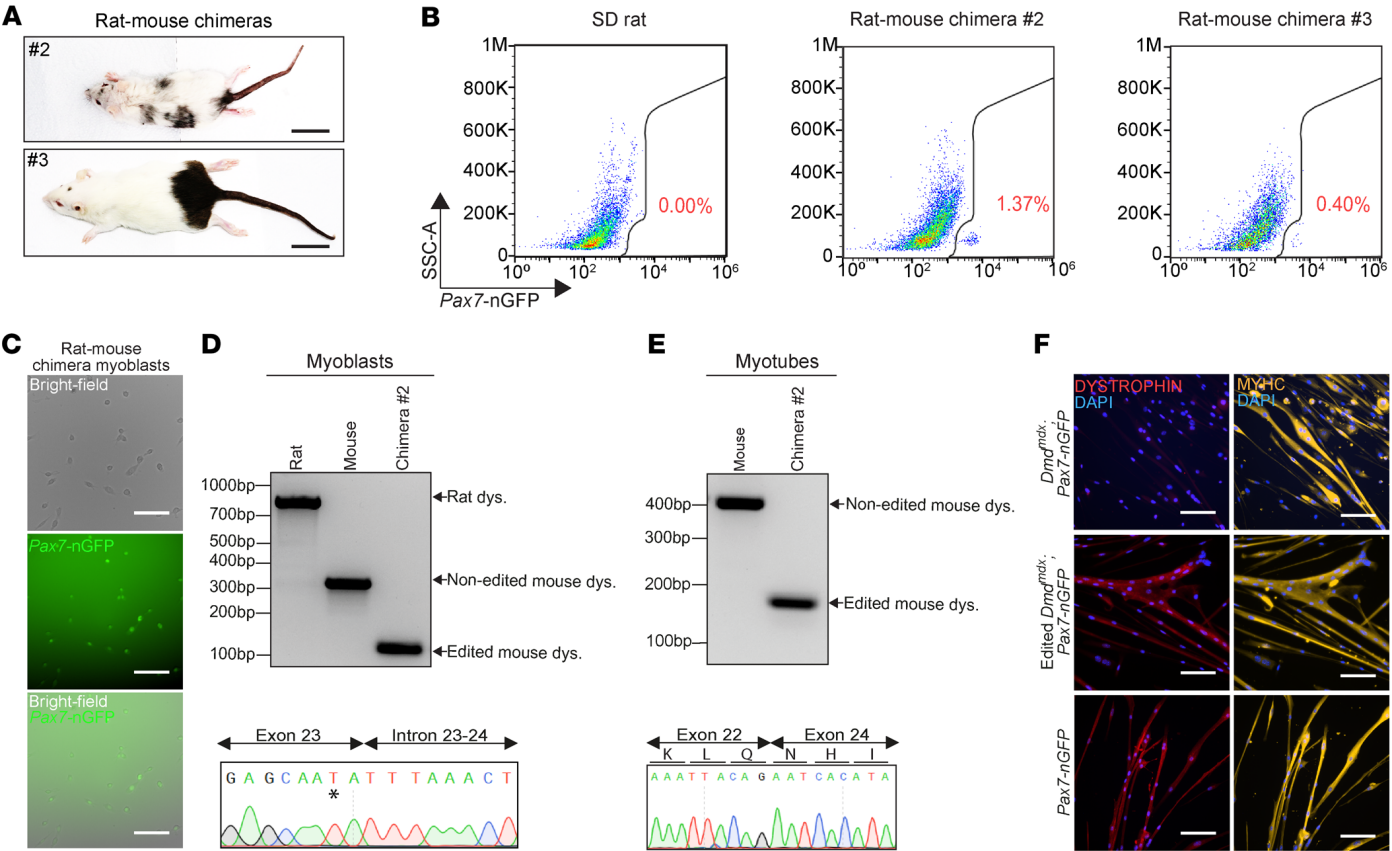
Establishment of gene-edited mouse myoblasts from satellite cells produced in rat-mouse chimeras. (**A**) Representative images of rat-mouse chimeras at 7 weeks of age. Scale bars: 3.5 cm. (**B**) Representative FACS plots demonstrating *Pax7*-nGFP expression in digested muscles from the indicated animals. (**C**) Representative images of FACS-purified *Dmd^mdx^*; *Pax7-nGFP* myoblasts from a rat-mouse chimera. Scale bars: 100 μm. (**D**) PCR for rat and mouse dystrophin in DNA extracted from myoblasts of the indicated animals, accompanied by Sanger sequencing of the PCR product. The black asterisk specifies the mdx mutation. (**E**) PCR for mouse dystrophin using cDNA from myotubes generated from myoblasts of the indicated animals, accompanied by Sanger sequencing of the PCR product, revealing reframing of the dystrophin gene at the cDNA level. (**F**) Immunostaining images for the indicated markers in myotubes derived from the specified myoblasts. Scale bars: 100 μm.

Given the detection of mouse muscle stem cells in rat-mouse chimera muscles, we then set out to investigate whether we can purify *Pax7*-nGFP^+^ satellite cells by FACS from the remaining interspecies chimeras, in which the extent of chimerism was varied, ranging between small black coat color patches to prominent contribution to dark coat color (Figure 6A and Supplemental Figure 7A). Most of these chimeras appeared healthy, although one chimera, which showed one of the highest chimerism based on coat color, demonstrated body asymmetry and malocclusion (Supplemental Figure 7A, top left), in line with previous reports that documented abnormalities in interspecies chimeras exhibiting extensive xenogeneic contribution (60, 61). A DNA genotyping analysis for dystrophin in muscles harvested from several chimeras revealed the presence of both the rat and mouse alleles, as well as the *Pax7-nGFP* transgene (Supplemental Figure 7, B and C). Remarkably, we were able to detect and purify by FACS a small population of *Pax7*-nGFP^+^ cells from the muscles of 3 of 6 interspecies chimeras (50%), corroborating the scRNA-seq analysis (Figure 6B and Supplemental Figure 7D). However, the percentage was smaller than observed in transgenic *Pax7-nGFP* mice (Supplemental Figure 7D). Most notably, when FACS-purified *Pax7*-nGFP^+^ cells were plated and expanded in vitro, they gave rise to myoblasts expressing GFP, and exclusively harbored the edited dystrophin band (Figure 6, C and D, and Supplemental Figure 7E). Subjecting these myoblasts to differentiation conditions resulted in the formation of myotubes that solely carried the edited dystrophin allele and downregulated reporter expression (Figure 6E and Supplemental Figure 7F). Finally, these myotubes were dystrophin positive, in contrast with unedited myotube control (Figure 6F). In conclusion, these findings demonstrate that gene-edited iPSC–derived mouse satellite cells can be obtained in interspecies rat-mouse chimeras, even without blastocyst complementation.

### Functional characterization of interspecies-derived muscle stem cells in vitro and in vivo.

Our results at this stage unveiled the in vivo generation of iPSC-derived satellite cells and derivative myoblasts in either intraspecies or interspecies chimeras. However, it remained unknown whether they are equivalent to one another or WT muscle stem cells with respect to their capacity to differentiate in vitro and in vivo, an important aspect for cell-based therapy. To address this query, we subjected WT and chimera-derived myoblasts to an in vitro differentiation protocol that produces multinucleated MYHC^+^ myotubes, which exhibited a similar fusion index (80%) (Supplemental Figure 8, A and B). These myotubes, whether derived from myoblasts of intraspecies or interspecies chimeras, expressed the sarcomere markers titin (TTN) and actinin α1 (ACTN1), demonstrating striation due to protein aggregation within myotubes (Supplemental Figure 8C). Lastly, the myotubes also contracted spontaneously, thus exhibiting their in vitro functionality (Supplemental Videos 1–4).

**Figure 7:**
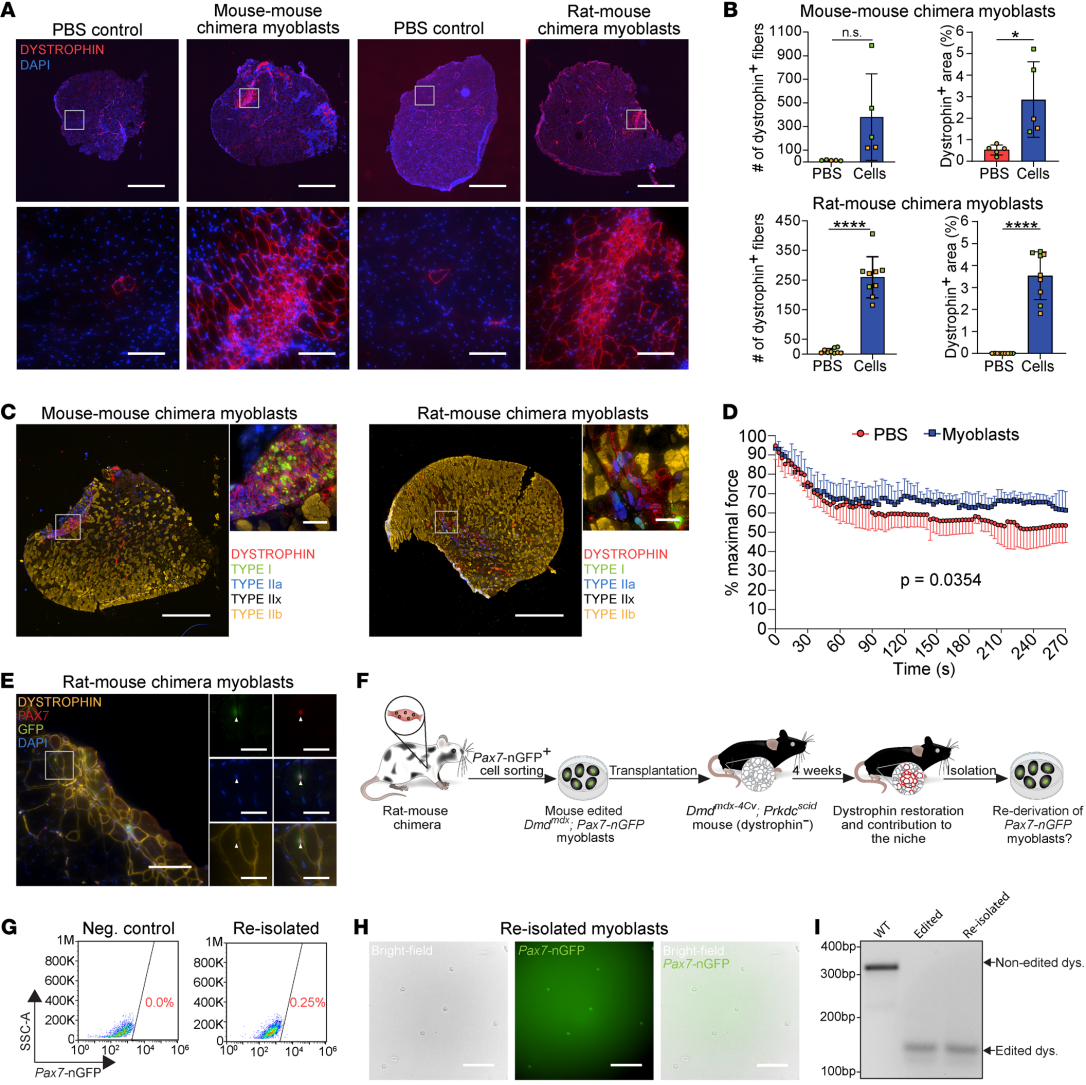
Mouse myoblasts produced in rats restore dystrophin and contribute to the niche in DMD mice. (**A**) Representative immunostaining for dystrophin in tibialis anterior (TA) muscle cross sections of *Dmd^mdx-4Cv^*; *Prkdc^scid^* mice 4 weeks after transplantation with the indicated cell lines. Scale bars: 1 mm (top) and 100 μm (bottom). (**B**) Quantification of the transplantation trials shown in **A**. *n* = 5 transplantation recipients for intraspecies chimera–derived myoblasts and *n* = 9 transplantation recipients for interspecies chimera–derived myoblasts. Each dot represents an individual transplanted muscle, with different dot colors specifying 2 different chimera-derived myoblast lines used for transplantations. Data are presented as mean ± SD. Statistical analysis was performed using a Student’s 2-tailed *t* test. *P ≤ 0.05, ****P ≤ 0.0001. (**C**) Representative immunostaining images for dystrophin and fiber typing in TA muscle cross sections of *Dmd^mdx-4Cv^*; *Prkdc^scid^* mice 4 weeks after transplantation with the indicated myoblasts. Scale bars: 1 mm (left) and 100 μm (right). LUTs were individually adjusted. (**D**) A graph illustrating force measurements during repeated tetanic contractions, showing the decline in TA muscle force of *Dmd^mdx-4Cv^*; *Prkdc^scid^* mice 4 weeks after transplantation with mouse-mouse and rat-mouse chimera–derived myoblasts compared to PBS control. *n* = 8 mice measured per group. Data are presented as mean ± SD. Statistical analysis was performed using a mixed effects model. (**E**) Representative immunostaining of TA muscle cross section from *Dmd^mdx-4Cv^*; *Prkdc^scid^* mice stained for the indicated markers 4 weeks after transplantation with the specified cell lines. Arrowheads point to colocalization of PAX7 expression and the *Pax7-nGFP* reporter in rare cells. Scale bars: 100 μm (left) and 25 μm (right). (**F**) Schematic representation of myoblast re-isolation from transplanted muscles. (**G**) Representative FACS plots showing the percentage of *Pax7*-nGFP^+^ cells detected in digested TA muscles of *Dmd^mdx-4Cv^*; *Prkdc^scid^* mice at 4 weeks following myoblast transplantation. (**H**) Representative images of re-isolated myoblasts. Scale bar: 100 μm. (**I**) PCR for dystrophin in DNA extracted from re-isolated myoblasts. Note the presence of only an edited band in edited and re-isolated myoblasts.

Next, we investigated whether *Pax7*-nGFP^+^ satellite cell–derived myoblasts from intraspecies or interspecies chimeras can restore dystrophin expression in muscles of *Dmd^mdx-4Cv^*; *Prkdc^scid^* mice following intramuscular transplantation. To this end, we transplanted 1 million edited *Dmd^mdx^; Pax7-nGFP* myoblasts into TA muscles that have been preinjured with CTX to facilitate myoblast engraftment, and included a PBS injection control for every transplantation trial. Four weeks after transplantation, we analyzed muscle cross sections for the presence of dystrophin expression. We observed a substantial increase (up to 40-fold) in dystrophin^+^ myofibers in muscles transplanted with edited *Dmd^mdx^*; *Pax7-nGFP* myoblasts compared with PBS-injected controls (Figure 7, A and B). Immunostaining analysis revealed the presence of various fiber types within engrafted dystrophin^+^ muscle areas, in accordance with our former results (Figure 4E, Figure 7C, and Supplemental Figure 9, A and B). We attribute the improved myoblast engraftment, in comparison with prior intraspecies myoblast transplantation trials (Figure 4, C and D), to the substantially higher number (approximately 10-fold) of transplanted myoblasts. Given the favorable outcome, we sought to assess whether dystrophin restoration manifests in functional improvement of dystrophic muscles. To this end, we subjected transplanted TA muscles to repeated tetanic contractions through electrical nerve stimulation. Following this manipulation, we observed a slower force decline in transplanted muscles compared with PBS-injected controls, although other force-related parameters were comparable between the 2 interventions (Figure 7D and Supplemental Figure 9, C and D). Of note, at 4 weeks after transplantation, approximately 20% of the dystrophin^+^ myofibers contained centrally located myonuclei, suggesting a regeneration process (Supplemental Figure 9E).

As a final objective, we wished to determine whether intraspecies- or interspecies-derived myoblasts could populate the satellite cell niche through identification of donor-derived PAX7^+^ cells in their normal anatomical location. Four weeks after transplantation, we detected rare PAX7^+^ cells in association with dystrophin^+^ myofibers that maintained *Pax7*-nGFP reporter expression (Figure 7E and Supplemental Figure 9F). Given the observation that transplanted myoblasts could be detected in the satellite cell anatomical position, we then wished to evaluate whether we can isolate these cells from transplanted muscles for further analysis. Strikingly, several weeks after transplantation, we were able to re-isolate a small population of GFP^+^ cells from TA muscles by FACS purification using the *Pax7-nGFP* reporter, enabling the reestablishment of *Pax7*-nGFP^+^ myoblasts (Figure 7, F–H). As further confirmation, a PCR analysis for dystrophin revealed only the presence of the edited allele in re-isolated myoblasts (Figure 7I). Lastly, these myoblasts readily fused into contractile myotubes that demonstrated a high fusion index and expressed a suite of sarcomere markers (Supplemental Figure 9, G–I and Supplemental Video 5). Together, these results demonstrate that mouse *Dmd^mdx^*; *Pax7-nGFP* iPSC–derived myoblasts produced in rat-mouse chimeras can efficiently restore dystrophin expression in limb muscles of DMD mice in vivo. Additionally, a small number of transplanted myoblasts remained as stem/progenitor cells in engrafted muscles, enabling re-derivation of myoblast lines.

## Discussion

**Figure 8:**
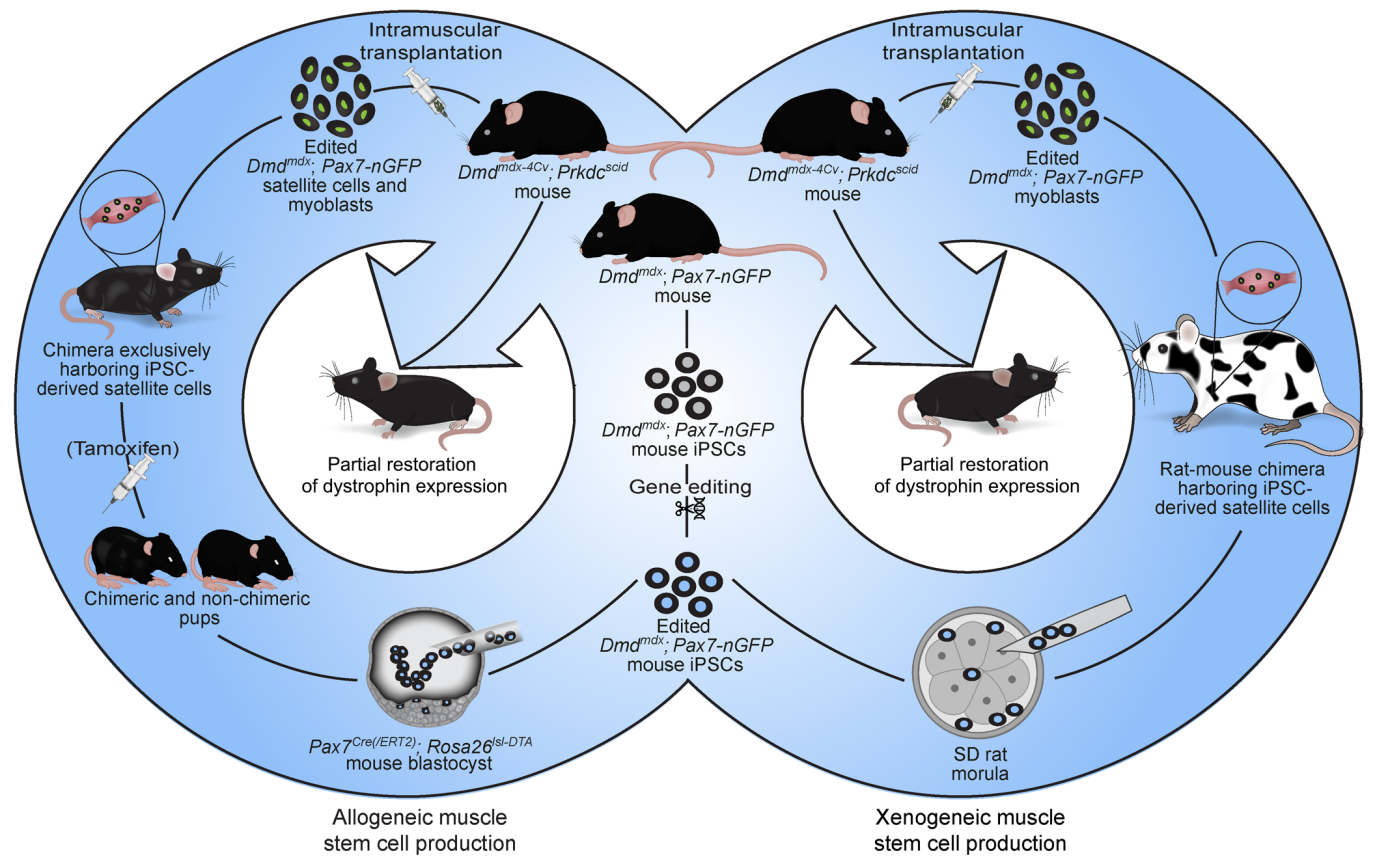
A schematic summarizing the key findings of the study.

In this study, we report on the generation of genetically corrected mouse iPSC-derived satellite cells and myoblasts in mouse-mouse and rat-mouse chimeras. In intraspecies chimeras, we employed 2 genetic ablation systems targeting host PAX7-expressing cells to preferentially obtain ESC-derived or gene-edited iPSC–derived satellite cells and derivative myoblasts, capable of restoring dystrophin expression in dystrophic muscles in vivo (Figure 8). To our surprise, we also observed substantial production of iPSC-derived satellite cells in chimeras even without an ablation system that targets PAX7-expressing cells during postnatal growth, prompting us to investigate the derivation of mouse satellite cells in rat-mouse chimeras. Strikingly, several rat-mouse chimeras contained an appreciable number of iPSC-derived and gene-edited mouse satellite cells, whose derivative myoblasts could efficiently restore dystrophin expression in vivo in muscles of DMD mice, as well as contributing to the stem cell reservoir (Figure 8).

Our work complements a prior study demonstrating that injection of WT ESCs into DMD blastocysts ameliorates disease pathology in *Dmd^mdx^* mice (62). Furthermore, it raises the possibility that a similar approach may enable the production of xenogeneic lineage-specific human muscle stem cells in interspecies chimeras for therapeutic purposes. In recent years, several studies reported on the contribution of human PSCs to chimerism in mouse, pig, and monkey embryos (63–69). However, adapting such a technique for production of human cells in full-term chimeras is associated with ethical concerns. Most notably, it will require means to exclude the generation of undesired human cell types such as brain cells or gametes in human-animal chimeras (70–72). To this end, the use of PSCs that carry a genetic mutation that prevents their differentiation into such cell types may provide a plausible solution, as shown in mice (71).

Unlike the derivation of human cells in full-term pig chimeras, the generation of human cells, including muscle cells, has been demonstrated in human-pig chimeric fetuses (39, 63, 66). Utilizing blastocyst complementation, a recent study reported on pig and human skeletal muscle formation by injection of pig PSCs or P53-null human iPSCs into pig embryos carrying a triple knockout in *MYOD*, *MYF5*, and *MYF6*, thereby enabling PSC colonization of the developing skeletal muscle lineage in chimeric embryos (66). Notably, PSC-derived PAX7-expressing muscle stem cells have been detected in pig-pig chimeras; however, they were not reported in pig-human chimeric embryos (66). Moreover, a notable caveat for production of xenogeneic skeletal muscle or other tissues and organs in interspecies chimeras is the presence of animal host–derived endothelium, mesenchyme, or other cell types, which may evoke immunological responses (70). The approach reported in our study may circumvent this major limitation, as potentially PSC-derived muscle stem cells can be purified by FACS in considerable numbers from interspecies chimeras for cell-based therapy, in the absence of undesired animal cells.

An additional highlight of the approach described in this study is that the PSCs were differentiated in vivo, thereby mitigating potential risk of residual PSCs to form teratomas upon transplantation, an obstacle when employing iPSCs to treat human patients (73). Furthermore, as the iPSCs differentiated into satellite cells in postnatal chimeras, this method ensures the generation of adult muscle stem cells, in comparison with myogenic precursor cells differentiated from PSCs in vitro, which may retain embryonic attributes (74). In relation to this effort, recent studies demonstrated that maturation of PSC-derived myogenic precursor cells requires an in vivo phase, rendering our approach complementary to these trials and potentially advantageous (75, 76). Furthermore, standing in support of our findings, a recent study reported that host muscle stem cell ablation in adult and dystrophic mice facilitated efficient engraftment and maturation of human iPSC–derived myogenic precursors in vivo (77). Looking ahead, it will be of interest to molecularly and functionally compare the muscle stem cells derived from PSCs in vivo using our system to other protocols that produce PSC-derived myogenic precursors in vitro.

For cell-based therapy in patients with DMD, our findings suggest that intraspecies chimera–derived satellite cells are superior to myoblasts, requiring fewer cells for comparable dystrophin restoration in vivo. However, by increasing myoblast numbers, both intraspecies and interspecies iPSC–derived myoblasts efficiently restored dystrophin expression in vivo, as previously reported (22). It is noteworthy to mention that, in the trials involving intraspecies chimeras, an ablation system was critical for producing an optimal quantity of satellite cells for transplantation. Therefore, it will be of interest to investigate whether this or a similar genetic ablation system of muscle stem cells can be used to exclusively generate PSC-derived xenogeneic satellite cells, as recently shown for rat bone marrow cells in mice (42).

In conclusion, our study presents a proof-of-principle approach that combines cellular reprogramming, genome engineering, and in vivo PSC differentiation to produce therapeutically competent allogeneic or xenogeneic muscle stem cells in animal hosts. With respect to implications that extend to human therapy, further work is certainly warranted to address major hurdles associated with the generation of human cells in animals. However, should these challenges be overcome, we envision that this study may pave the way for producing human satellite cells in large animals for the treatment of muscle diseases.

## Methods

The experimental procedures and reagents utilized in this study are detailed in the Supplemental Methods section.

### Sex as biological variable.

Our study examined animals of both sexes, appropriately matched for each experiment.

### Statistics.

Statistical analysis was performed with Prism (versions 9.2.0 and 10, GraphPad Software) and data are presented as mean ± SD. *P* values of 0.05 or less were considered statistically significant. Across all figures, statistical significance is represented using asterisks: *P ≤ 0.05; **P *≤* 0.01; ***P ≤ 0.001; ****P ≤ 0.0001. Nonsignificant differences are labeled as “n.s.” Differences were evaluated using Student’s 2-tailed *t* test and 1- or 2-way ANOVA. Mixed effects model was used to analyze the difference in muscle force reduction between control and transplanted muscles following repeated tetanic contractions.

### Study approval.

The present study was approved by the Federal Food Safety and Veterinary Office, Cantonal veterinary office in Zurich, and granted animal experimental license numbers ZH246/18, ZH177/18, ZH002/22, ZH032/23, and FormG-135.

### Data availability.

All plasmids used in this study can be obtained from the authors upon request, or from Addgene (https://www.addgene.org/Ori_Bar-Nur/). Bulk RNA-seq and scRNA-seq data sets can be accessed in the NCBI Gene Expression Omnibus (GEO) repository under accession number GSE255196. The top 20 markers used to determine the identity of each cell cluster in the scRNA-seq data are provided in Supplemental Data Set 1. Individual data values presented in graphs across all figures are available in the Supporting Data Values file. Complete unedited agarose gel images are provided in the Supplemental Unedited Blot and Gel Images file.

## Author contributions

The study was conceptualized by AL, SAD, JZ, and OBN. Experiments involving intraspecies chimeras were performed by AL, SAD, and NB. Furthermore, AL, JZ, and NB performed experiments involving interspecies chimeras. The blastocyst and morulae injections were performed by MTS. Muscle force measurements and analysis were carried out by RF, AL, CH, EM, and KDB. Intramuscular cell transplantation and analysis were performed by AL, NB, SAD, and GB. Additionally, AL, SAD, JZ, NB, PG, and XQ carried out molecular biology analyses and FN, CLT, AL, and AG analyzed the RNA-seq data. The manuscript was written by AL, SAD, JZ, and OBN. The study was supervised by OBN.

## Supplementary Material

Supplemental data

Supplemental data set 1

Unedited blot and gel images

Supplemental video 1

Supplemental video 2

Supplemental video 3

Supplemental video 4

Supplemental video 5

Supporting data values

## Version 1 05/07/2024

In-Press Preview

## Version 2 06/17/2024

Electronic publication
