# Supplementary material for: Generation of allogeneic and xenogeneic functional muscle stem cells for intramuscular transplantation
Source: J Clin Invest. 2024 May 7;134(12):e166998. doi: 10.1172/JCI166998 (PMC11178549; doi:10.1172/JCI166998)
Supplement: Supplemental data [file jci-134-166998-s251.pdf]

## Supplemental Figures

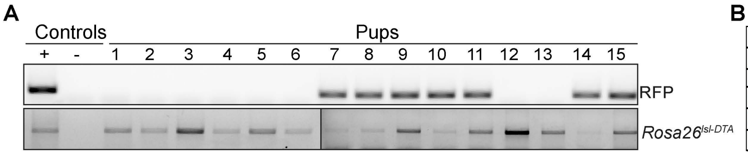


**B**

| Marker | Cell type marked | Fluorophore/Filter |
|---|---|---|
| RFP | ESC-derived cells | mCherry |
| CD31 | Endothelial cells | APC |
| CD45 | Hematopoietic cells | APC |
| SCA1 | Mesenchymal cells | APC |
| ITGA7 | Satellite cells | APC-AF750 |

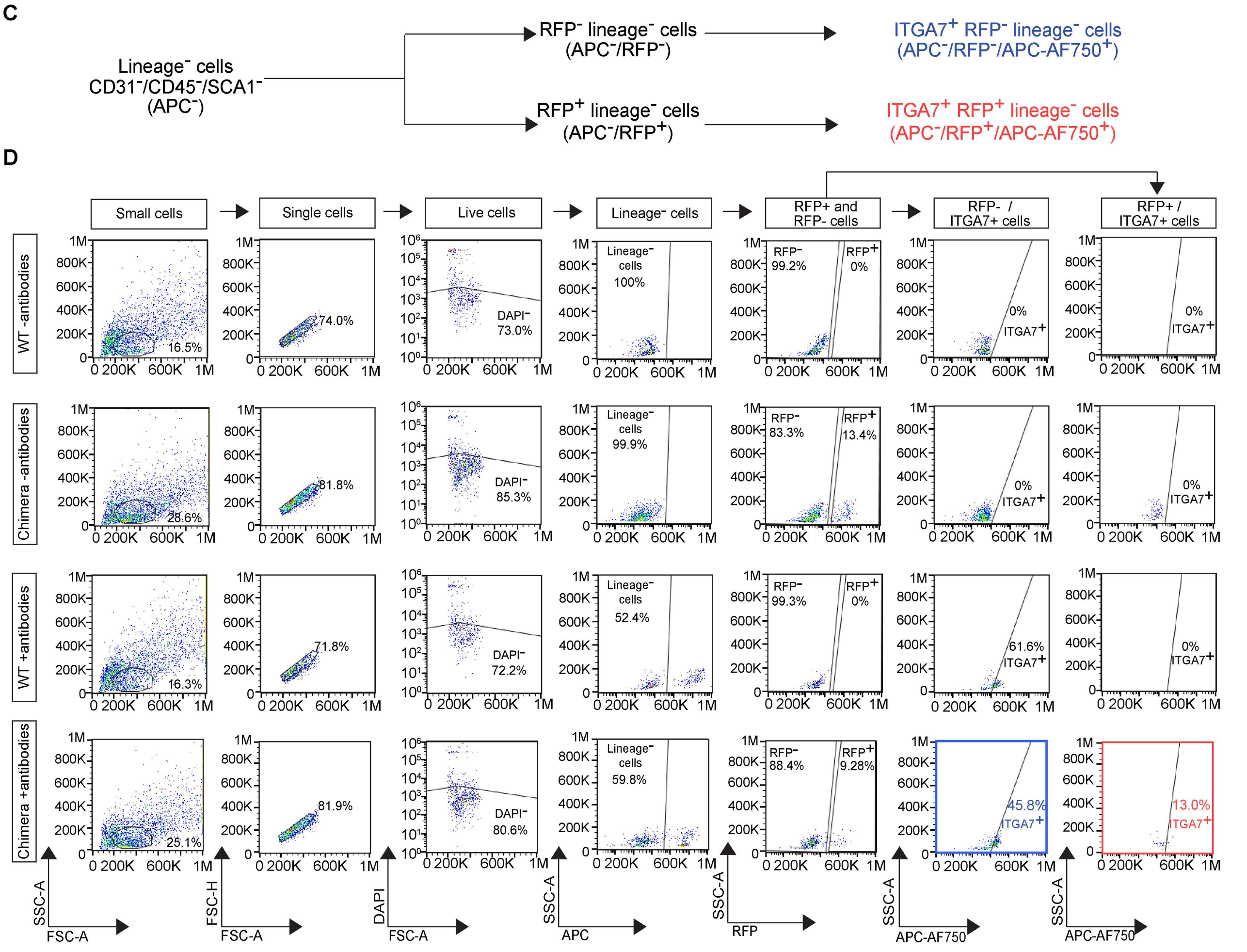


### Supplemental Figure 1

**(A)** Genotyping for the RFP transgene and *Rosa26*$^{lsl\text{-}DTA}$ allele using PCR in non-chimeric and chimeric mice. Results of the PCR reaction for the *Rosa26*$^{lsl\text{-}DTA}$ allele were run on the same gel but were noncontiguous. **(B)** Surface markers used for satellite cell isolation through FACS. **(C)** FACS strategy for purification of satellite cells from chimeras. **(D)** Representative FACS plots showing purification strategy of ITGA7$^{+}$ satellite cells that are either RFP positive or negative from muscles of chimeras.

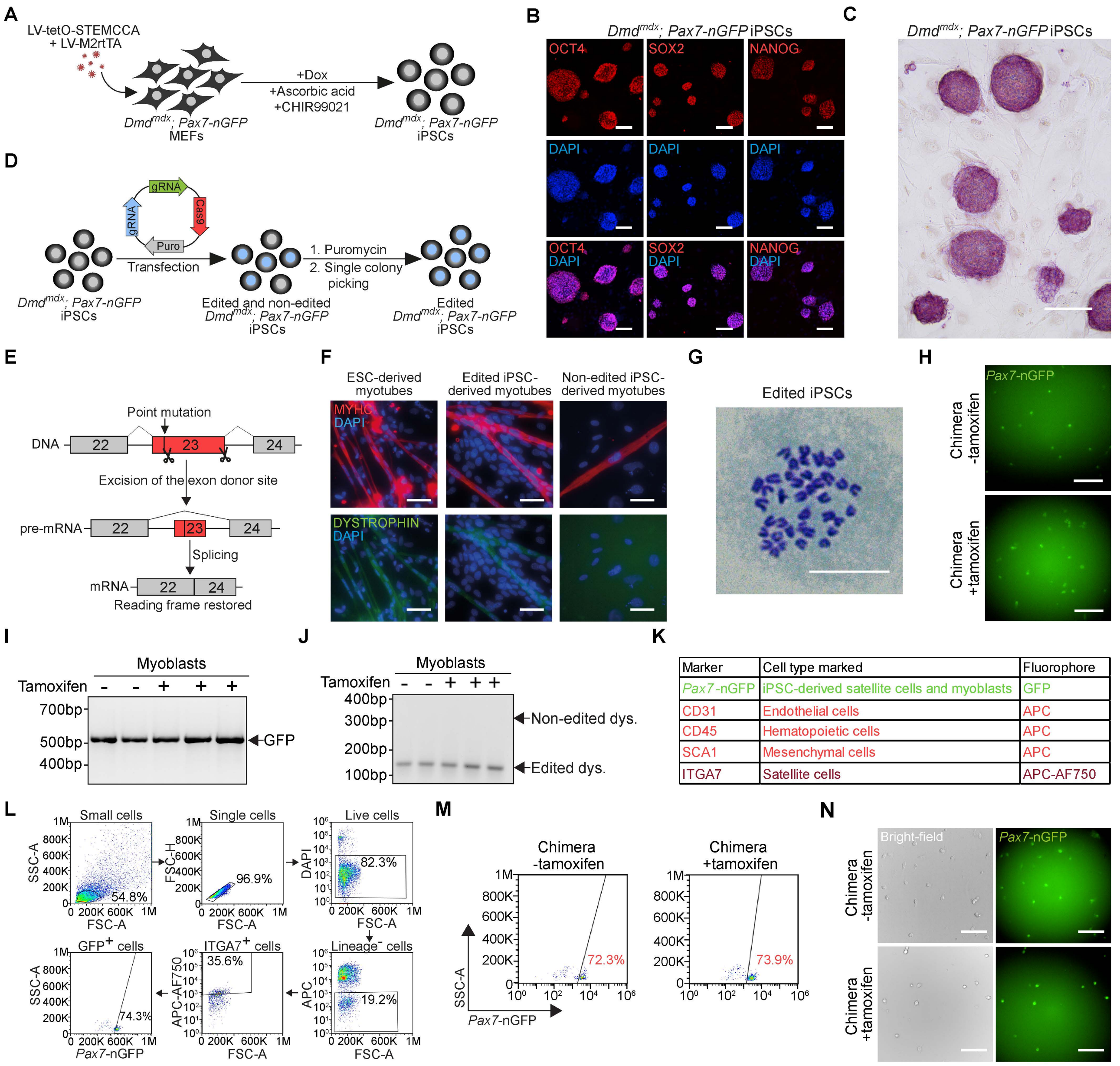


| Marker | Cell type marked | Fluorophore |
|---|---|---|
| *Pax7*-nGFP | iPSC-derived satellite cells and myoblasts | GFP |
| CD31 | Endothelial cells | APC |
| CD45 | Hematopoietic cells | APC |
| SCA1 | Mesenchymal cells | APC |
| ITGA7 | Satellite cells | APC-AF750 |

## Supplemental Figure 2

**(A)** Schematic overview of iPSC derivation from *Dmd*$^{mdx}$*; Pax7-nGFP* MEFs. MEFs, mouse embryonic fibroblasts; Dox, doxycycline. **(B)** Representative immunostaining images for the indicated pluripotency markers in *Dmd*$^{mdx}$; *Pax7-nGFP* iPSCs. Scale bar, 100µm. **(C)** Alkaline phosphatase staining of *Dmd*$^{mdx}$; *Pax7-nGFP* iPSCs. Scale bar, 100µm. **(D)** A schematic outlining the strategy taken to correct the dystrophin mutation through gene editing of *Dmd*$^{mdx}$; *Pax7-nGFP* iPSCs. **(E)** Schematic representation of the CRISPR/Cas9 gene editing strategy used to correct the dystrophin mutation by exon skipping. **(F)** Immunostaining for the indicated muscle markers in myotubes differentiated from the specified cells. Scale bar, 50µm. **(G)** Karyotype of edited *Dmd*$^{mdx}$; *Pax7-nGFP* iPSCs showing a normal chromosome count. Scale bar, 25µm. **(H)** Representative fluorescence images of *Dmd*$^{mdx}$; *Pax7-nGFP* myoblasts that have been FACS-purified using the *Pax7*-nGFP reporter from tamoxifen-treated or non-treated chimeras. Scale bar, 100µm. LUTs were individually adjusted. **(I)** PCR genotyping for the *Pax7-nGFP* allele in myoblasts derived from the indicated chimeras. **(J)** PCR for dystrophin in *Dmd*$^{mdx}$; *Pax7-nGFP* myoblasts that have been FACS-purified from muscles of the indicated chimeras. **(K)** Surface markers used for FACS-purification of ITGA7$^{+}$ satellite cells. **(L)** FACS strategy for the sorting of *Pax7*-nGFP$^{+}$ satellite cells from chimeras. **(M)** Representative FACS plots showing *Pax7-nGFP* expression in ITGA7$^{+}$ satellite cells from the specified animals. **(N)** Representative fluorescence images of ITGA7$^{+}$ satellite cell-derived myoblasts of the indicated animals and conditions. Scale bar, 100µm. LUTs were individually adjusted.

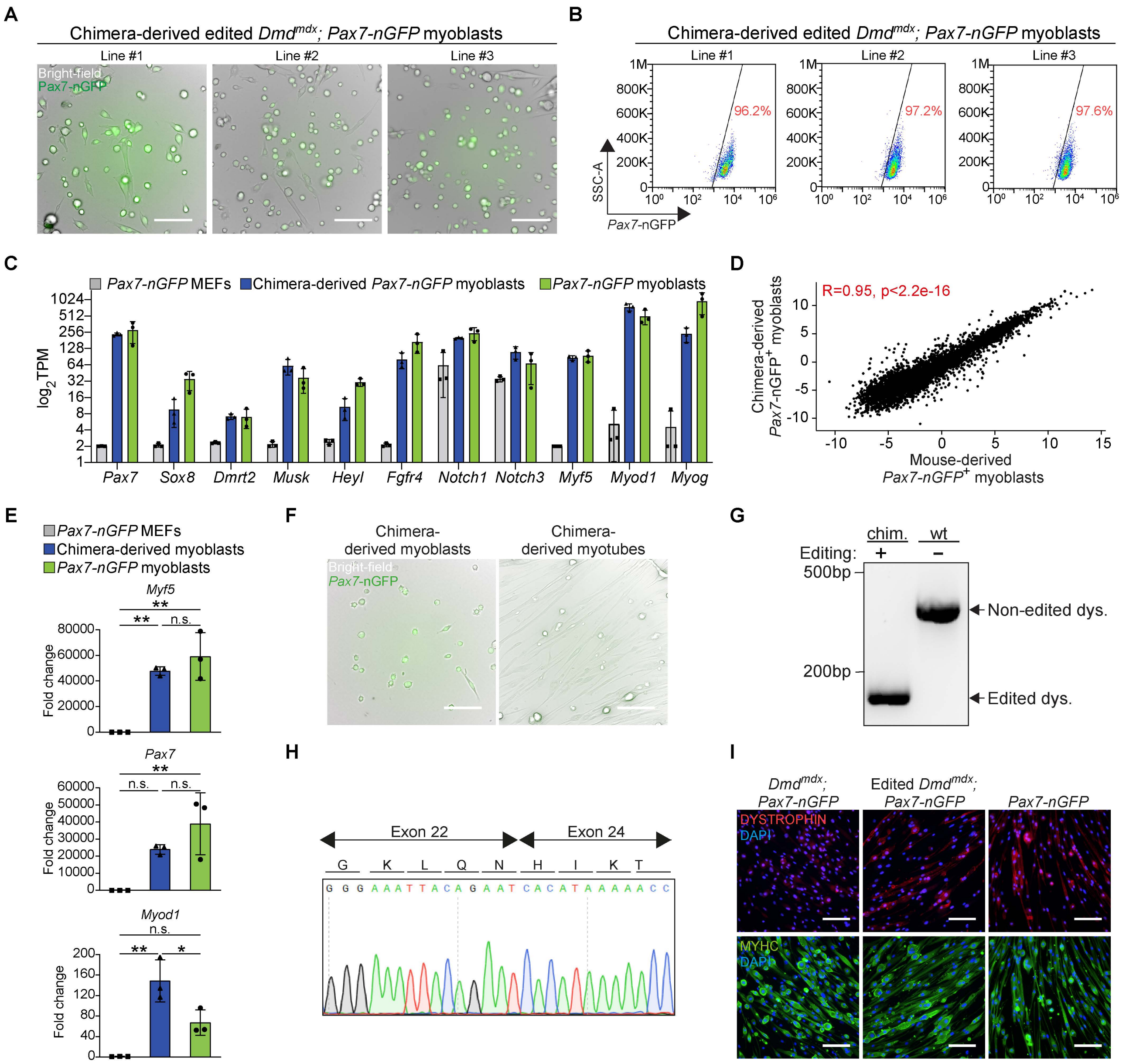


**Supplemental Figure 3**

**(A)** Representative bright-field and fluorescence images of edited *Dmd*$^{mdx}$; *Pax7-nGFP* myoblast lines isolated from three different intraspecies chimeras. Scale bar, 100µm. LUTs were individually adjusted for the bright-field channel. **(B)** Representative FACS plots illustrating *Pax7-nGFP* reporter expression in the specified myoblast lines, corresponding to the cell lines shown in (A). **(C)** Bar plots based on bulk RNA-Seq showing the expression of the indicated genes in the respective cell lines. N=3 cell lines derived from 3 different animals per group. TPM, Transcripts per million. **(D)** Scatter plot depicting correlation based on bulk RNA-Seq of the specified samples. N=3 cell lines derived from 3 different animals per group. **(E)** RT-qPCR demonstrating increase in the expression of myoblast-related genes in myoblasts vs. negative MEF control. N=3 cell lines derived from 3 different animals per group, data is presented as mean±SD. Statistical analysis was performed using an ordinary one-way ANOVA. **(F)** Representative bright-field and fluorescence images of intraspecies chimera-derived edited *Dmd*$^{mdx}$; *Pax7-nGFP* myoblasts and derivative myotubes. Note that reporter expression is downregulated upon differentiation. Scale bar, 100µm. **(G)** PCR for dystrophin using cDNA of intraspecies chimera-derived edited *Dmd*$^{mdx}$; *Pax7-nGFP* myotubes (chim.) and *Pax7-nGFP* (wt) myotubes. **(H)** Sanger sequencing of the dystrophin PCR product reveals the successful ligation of exon 22 and 24 at the cDNA level of chimera-derived myotubes, indicating successful reframing. **(I)** Immunostaining for the indicated muscle markers in myotubes differentiated from the indicated myoblasts. Notably, dystrophin expression is detected solely in chimera-derived edited myotubes and the positive *Pax7-nGFP* control. Scale bar, 100µm.

**A**

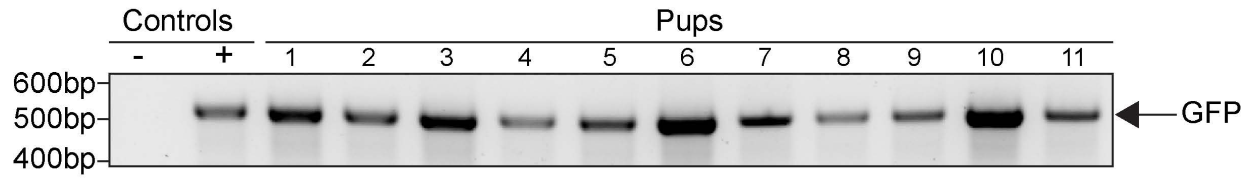


**B**

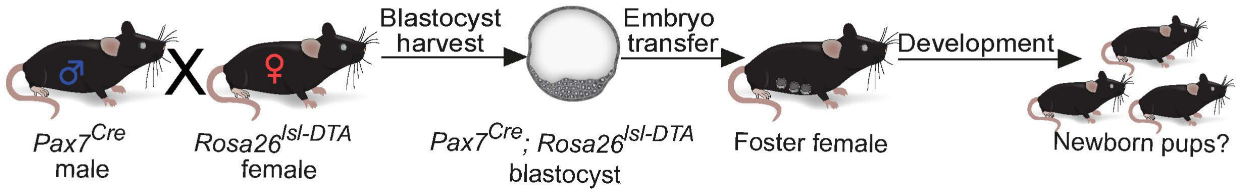


**C**

| # of blastocyst donors | # of flushed embryos | # of transferred blastocysts | # of fosters | Born |
|---|---|---|---|---|
| 5 | 30 | 22 | 2 | 0 |
| 5 | 45 | 41 | 4 | 0 |

**D**

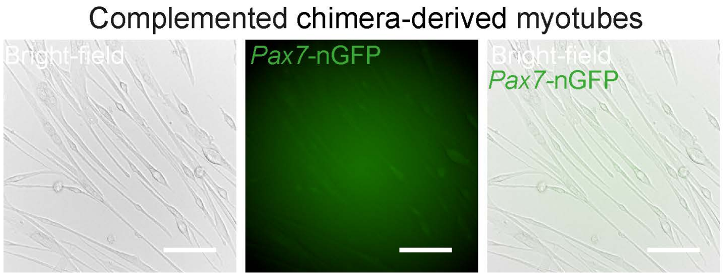


**Supplemental Figure 4**

**(A)** PCR genotyping for the *Pax7-nGFP* allele in DNA extracted from *Dmd*$^{mdx}$*; Pax7-nGFP* / *Pax7*$^{Cre}$ x *Rosa26*$^{lsl\text{-}DTA}$ intraspecies chimeras. **(B)** Schematic representation of the objective to assess the viability of *Pax7*$^{Cre}$ x *Rosa26*$^{lsl\text{-}DTA}$ blastocysts to develop to term. **(C)** Table showing the results of the experiment depicted in (B). Note that no *Pax7*$^{Cre}$ x *Rosa26*$^{lsl\text{-}DTA}$ embryos developed to term. **(D)** Representative bright-field and fluorescence images showing complemented *Pax7*$^{Cre}$ x *Rosa26*$^{lsl\text{-}DTA}$ chimera-derived edited *Dmd*$^{mdx}$; *Pax7-nGFP* myotubes. Note that *Pax7-nGFP* reporter expression is downregulated upon differentiation. Scale bar, 100µm.

**A**

| | Animal weight (g) | Isolated muscle (g) | Approximate # of GFP+ cells | # of transplanted sat. cells | % dystrophin area (sat. cells) | % dystrophin area (PBS) | # of transplanted myobl. | % dystrophin area (myobl.) | % dystrophin area (PBS) |
|---|---|---|---|---|---|---|---|---|---|
| Chimeras | 27.0 | 1.91 | 120 000 | 20 000 | 3.819836835 | 0.085420385 | 20 000 | 1.132252407 | 0.737956666 |
| | 26.5 | 1.76 | 120 000 | 90 000 | 1.160717828 | 0.357146627 | 100 000 | 0.853669053 | 0.601949721 |
| | 27.4 | 1.90 | 120 000 | 90 000 | 5.057658184 | 0.556737697 | 100 000 | 1.223092459 | 0.35586398 |
| | 28.2 | 1.50 | 112 500 | 110 000 | 2.438571094 | 0.470847847 | 110 000 | 0.621799377 | 0.491393212 |
| | 25.5 | 1.20 | 93 000 | 91 000 | 3.041457641 | 0.361479432 | 90 000 | 0.404733708 | 0.258462072 |
| | 30.1 | 1.77 | 127 500 | 125 000 | 5.512067384 | 0.188850601 | 125 000 | 1.137681316 | 0.250941779 |
| *Pax7-nGFP* | 21.0 | 1.81 | 120 000 | 100 000 | 3.79126775 | 0.419345312 | 100 000 | 2.214798279 | 0.17333666 |
| | 29.3 | 1.78 | 122 000 | 120 000 | 5.664846067 | 0.109196947 | 120 000 | 0.683909555 | 0.373608451 |
| | 27.2 | 1.20 | 152 000 | 150 000 | 7.277878292 | 0.33457772 | 150 000 | 0.599471909 | 0.332466248 |
| | 17.2 | 1.0 | 98 000 | 96 000 | 3.902463815 | 0.233070825 | 96 000 | 1.514157459 | 0.629777117 |

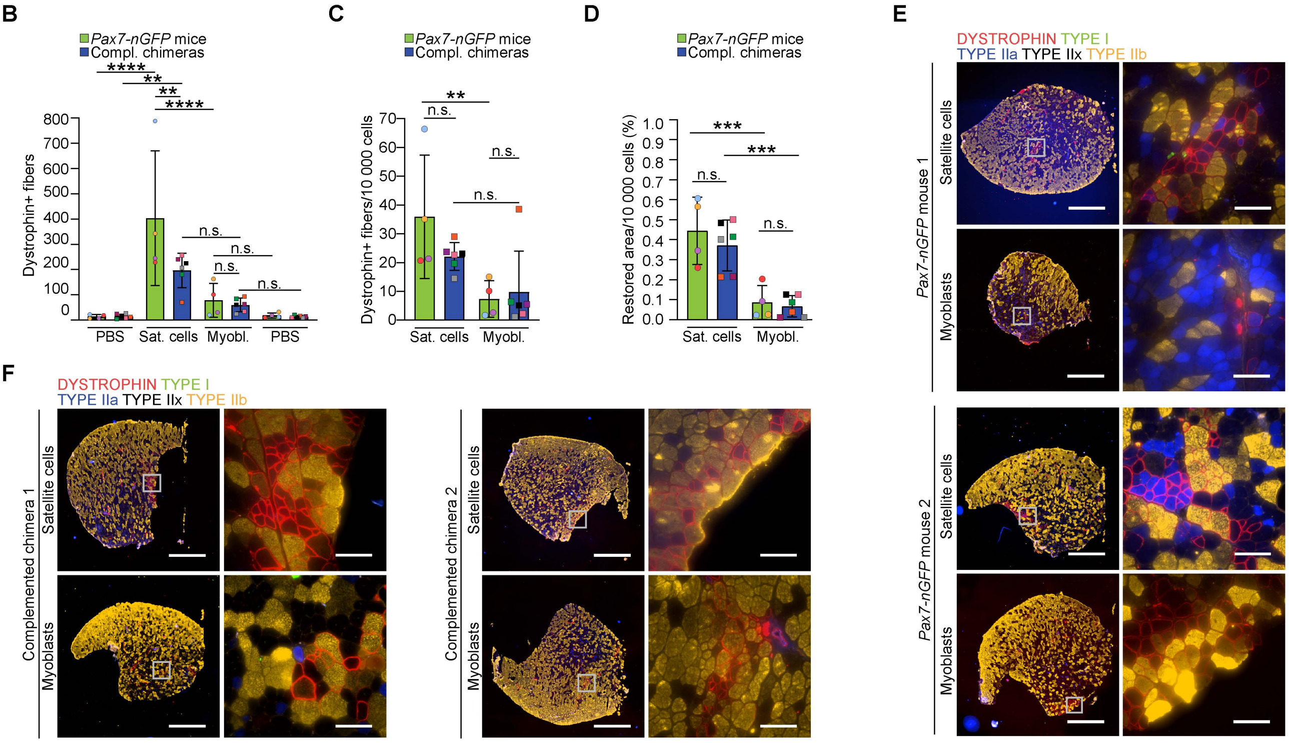


**Supplemental Figure 5**

**(A)** A table showing the number of *Pax7*-nGFP$^+$ satellite cells and myoblasts obtained from *Pax7-nGFP* mice and complemented *Pax7$^{Cre}$* x *Rosa26$^{lsl\text{-}DTA}$* chimeras that were used for transplantation into tibialis anterior (TA) muscles of *Dmd$^{mdx\text{-}4Cv}$; Prkdc$^{scid}$* mice. In addition, the quantitative results of the transplantation experiment are provided. The colors correspond to the datapoints shown in (B – D) and in Figure 4D. Sat. cells, satellite cells; myobl., myoblasts. **(B)** Number of dystrophin$^+$ myofibers in TA muscle cross-section of *Dmd$^{mdx}$; Prkdc$^{scid}$* mice at 4 weeks post-transplantation with the specified cell lines in comparison to PBS control. Each dot represents one recipient and colors indicate cells derived from the same donor. N=4 donors for *Pax7-nGFP* mouse-derived cells and N=6 donors for chimera-derived cells. Data is presented as mean±SD. Statistical analysis was performed using a two-way ANOVA. **(C)** The number of dystrophin$^+$ fibers in TA muscle cross-section normalized to 10,000 injected cells and PBS control. Each dot represents one recipient and colors indicate cells derived from the same donor. N=4 donors for *Pax7-nGFP* mouse-derived cells and N=6 donors for chimera-derived cells. Data is presented as mean±SD. Statistical analysis was performed using a two-way ANOVA. **(D)** Quantification of dystrophin$^+$ area in TA muscle cross-section of *Dmd$^{mdx\text{-}4Cv}$; Prkdc$^{scid}$* mice at 4 weeks post-transplantation with the specified cell lines. Results were normalized to 10,000 injected cells and PBS control. Each dot represents one recipient and colors indicate cells derived from the same donor. N=4 donors for *Pax7-nGFP* mouse-derived cells and N=6 donors for chimera-derived cells. Data is presented as mean±SD. Statistical analysis was performed using a two-way ANOVA. **(E)** Representative immunostaining images for dystrophin and fiber typing in TA muscle cross-sections of *Dmd$^{mdx\text{-}4Cv}$; Prkdc$^{scid}$* mice at 4 weeks post-transplantation with the specified *Pax7-nGFP* mouse-derived cell lines. Scale bar, 1mm (left) and 100µm (right). **(F)** Representative immunostaining images for dystrophin and fiber typing in TA muscle cross-sections of *Dmd$^{mdx\text{-}4Cv}$; Prkdc$^{scid}$* mice at 4 weeks post-transplantation with the specified complemented chimera-derived cell lines. Scale bar, 1mm (left) and 100µm (right).

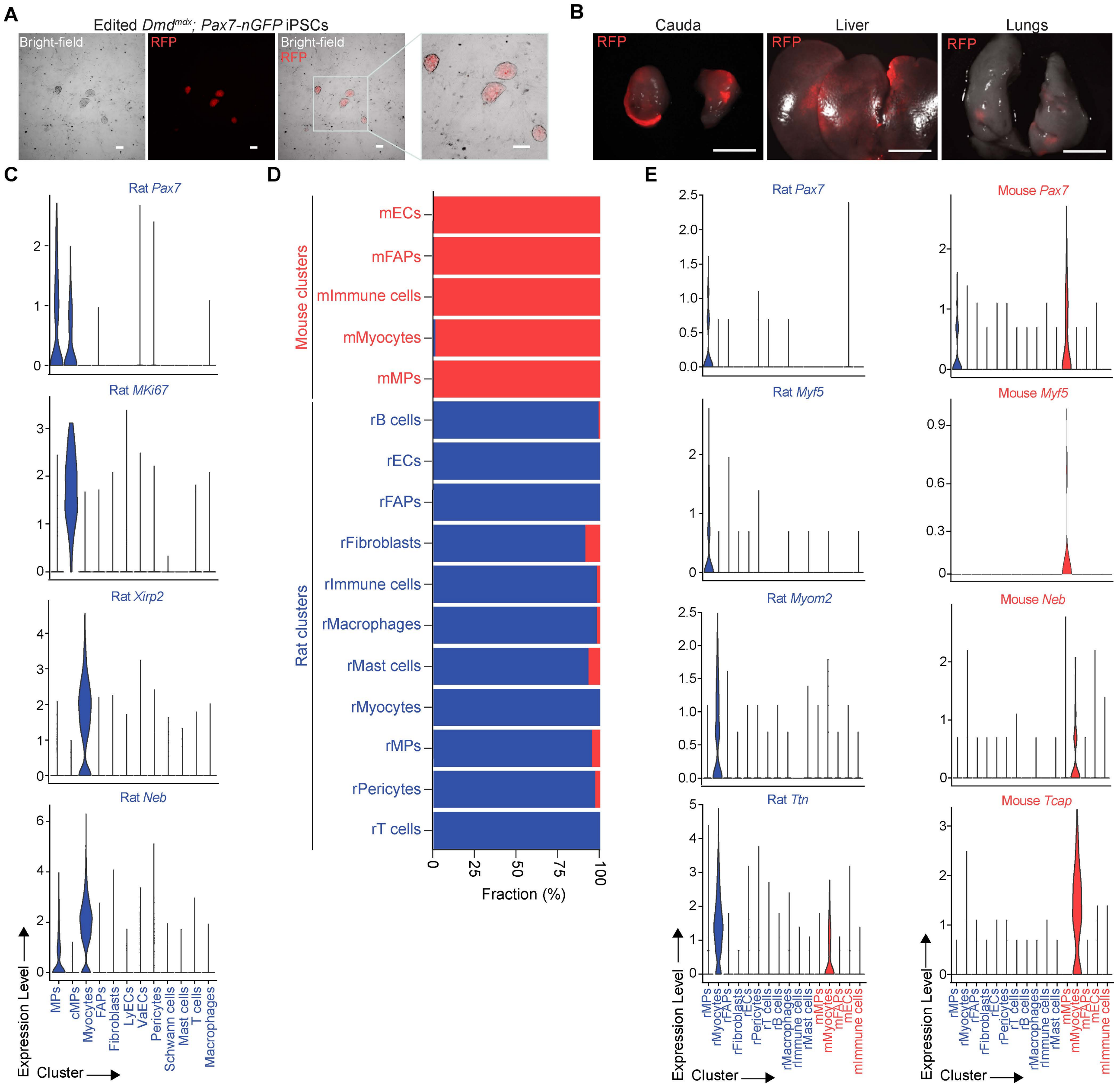


**Supplemental Figure 6**

**(A)** Representative bright-field and fluorescence images of RFP-labeled and edited *Dmd*$^{mdx}$; *Pax7-nGFP* iPSCs. Scale bar, 100μm. **(B)** Images of RFP expression in organs derived from rat-mouse chimera #1. Scale bar, 1cm. **(C)** Violin plots based on scRNA-Seq showing the expression of the specified rat myogenic markers in the respective cell populations comprising SD rat muscles. MPs, myogenic progenitors; cMPs, cycling myogenic progenitors; VaECs, vascular endothelial cells; LyECs, lymphatic endothelial cells; FAPs, fibro-adipogenic progenitors. **(D)** The top 100 markers per cell cluster from rat-mouse chimera muscle scRNA-Seq data colored by species origin. Note that very few mouse markers are assigned as rat due to species sequence similarity. The letters "r" and "m" denote rat and mouse, respectively. MPs, myogenic progenitors; ECs, endothelial cells; FAPs, fibro-adipogenic progenitors. **(E)** Violin plots showing the expression of the indicated rat and mouse myogenic markers in specific cell populations comprising rat-mouse chimera muscles. The letters "r" and "m" denote rat and mouse, respectively. Note that mouse *Pax7* is also assigned to rat MPs due to high sequence similarity between the two species.

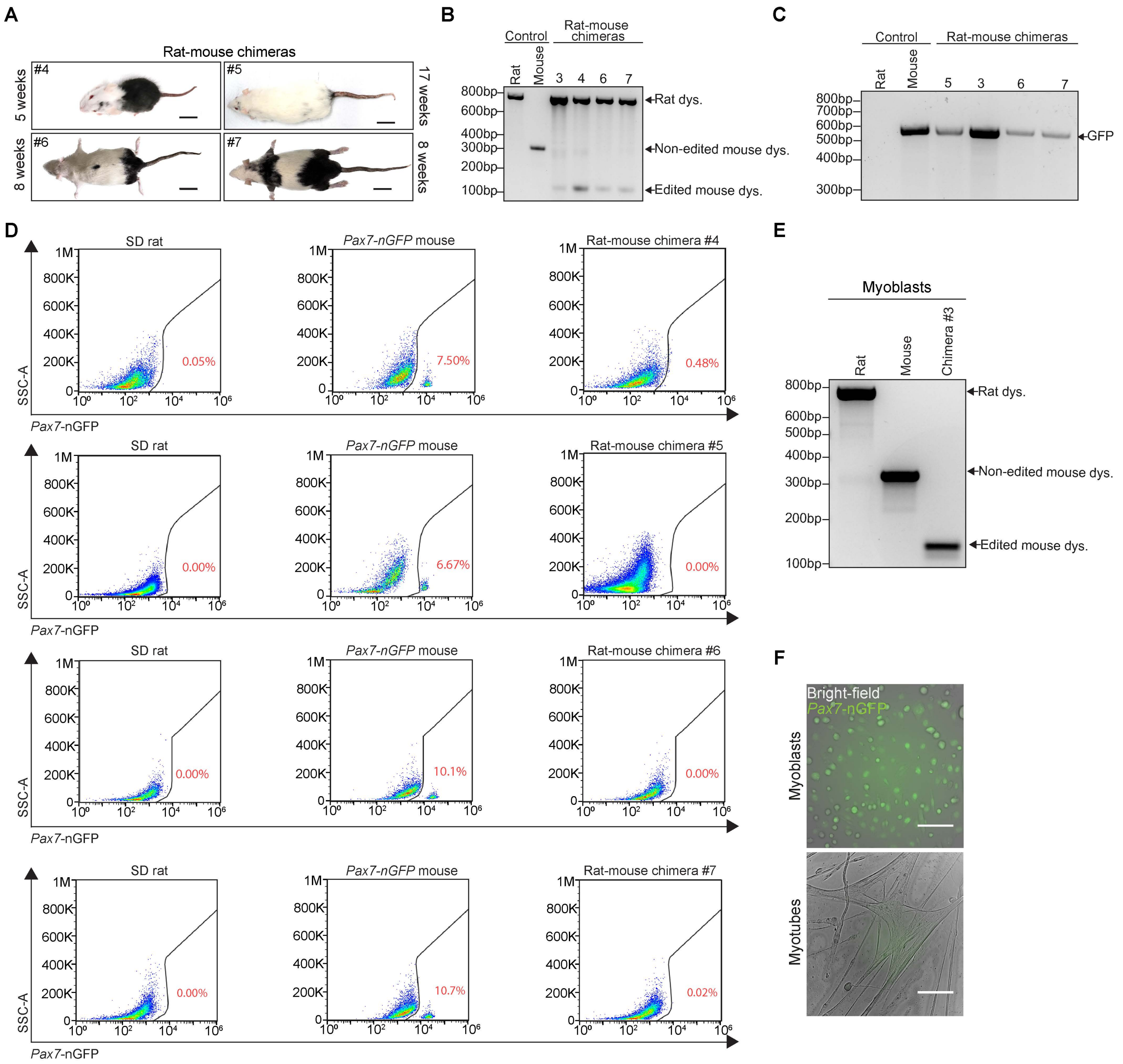


**Supplemental Figure 7**

**(A)** Photos of rat-mouse chimeras at the listed ages. Scale bar, 3.5cm. **(B)** PCR for rat and mouse dystrophin using DNA isolated from muscle lysates of the indicated animals. **(C)** PCR genotyping for the *Pax7-nGFP* transgene in muscle lysates of the specified animals. **(D)** Representative FACS plots of *Pax7-nGFP* reporter expression in skeletal muscles isolated from SD rat, *Pax7-nGFP* mouse and specified rat-mouse chimeras. **(E)** PCR for rat and mouse dystrophin in DNA isolated from *Pax7*-nGFP+ myoblasts obtained from rat-mouse chimera #3, as well as from *Pax7-nGFP* mouse-derived myoblasts and rat muscle-derived cells. **(F)** Representative bright-field and fluorescence images of rat-mouse chimera-derived edited *Dmd*$^{mdx}$; *Pax7-nGFP* myoblasts and derivative myotubes. Scale bar, 100μm. Bright-field settings differ between images, whereas the GFP channel is identical.

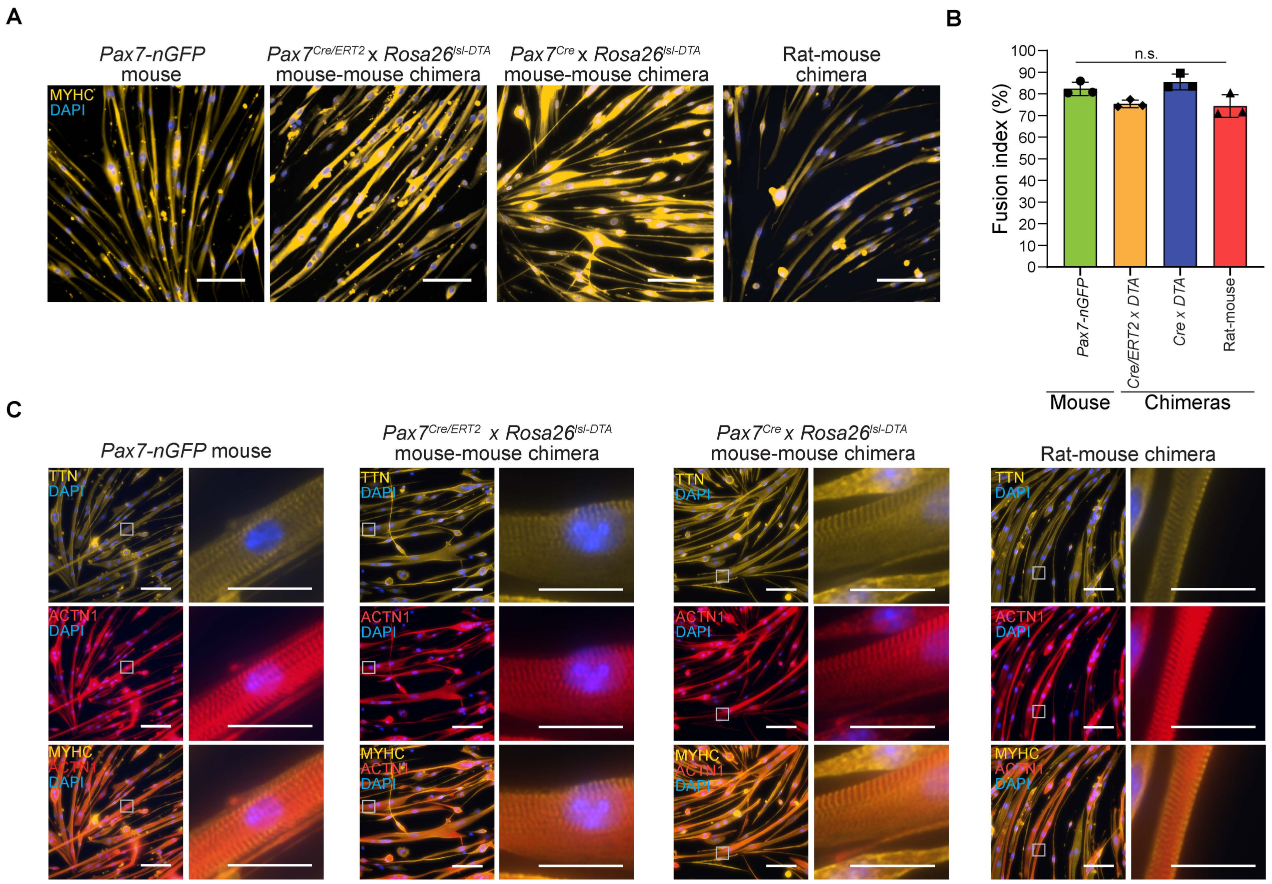


**Supplemental Figure 8**

**(A)** Representative immunostaining images of myosin heavy chain (MYHC) in myotubes differentiated from the indicated myoblast lines. Scale bar, 100μm. **(B)** Fusion index analysis of myotubes differentiated from the indicated myoblast lines. N=3 images per myoblast line. One myoblast line was used per animal group. Data is presented as mean±SD. The "n.s." line indicates no significant difference between *Pax7-nGFP* and all other groups. Statistical analysis was performed using a one-way ANOVA. **(C)** Representative immunostaining images for the indicated skeletal muscle markers in myotubes differentiated from the specified myoblasts. Scale bar, 100μm (left), 25μm (right).

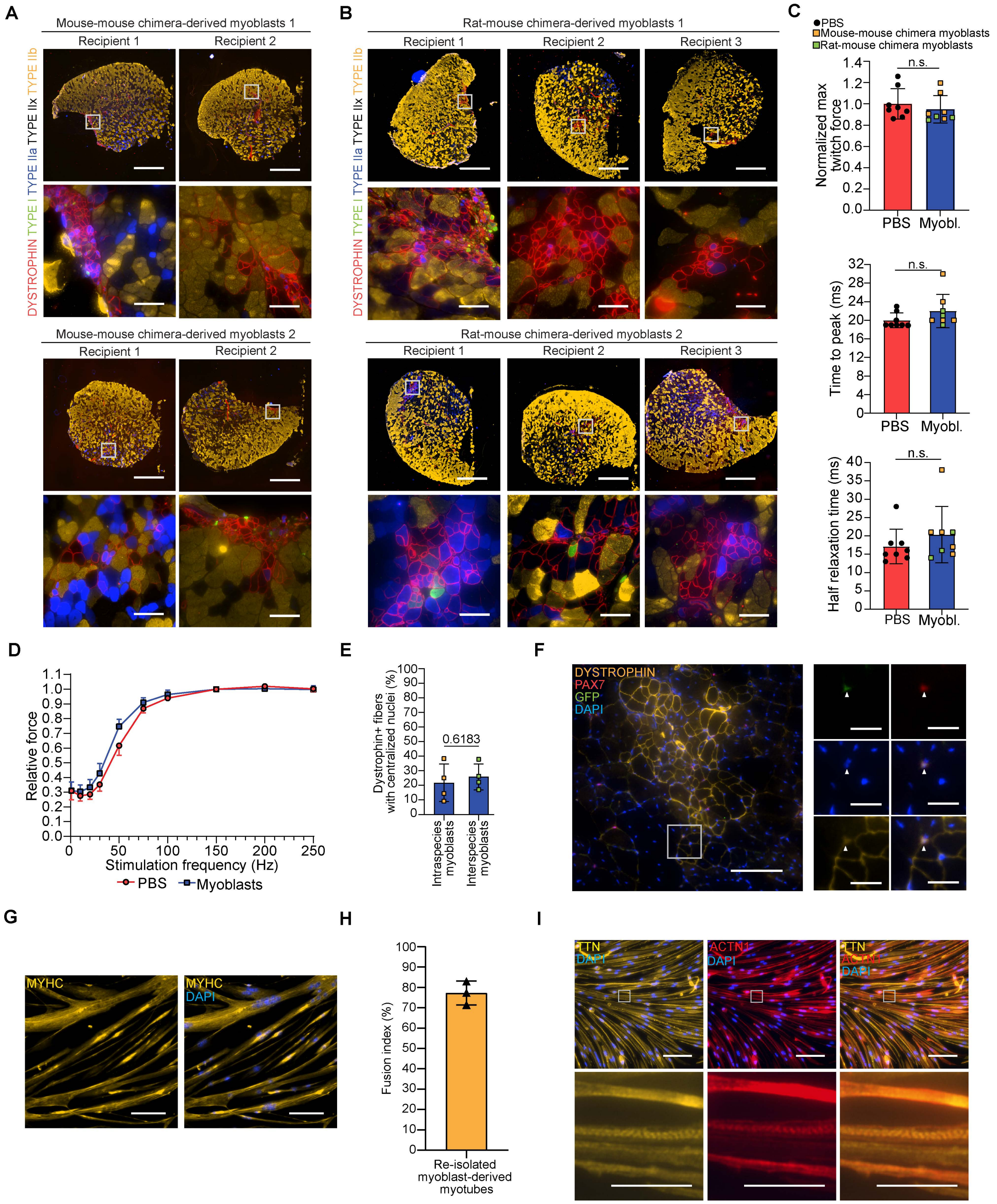


**Supplemental Figure 9**

**(A)** Representative immunostaining images for dystrophin and fiber typing in TA muscle cross-sections of *Dmd*$^{mdx\text{-}4Cv}$*; Prkdc*$^{scid}$ mice at 4 weeks post-transplantation with the specified mouse-mouse chimera-derived myoblasts. Scale bar, 1mm (top), 100μm (bottom). **(B)** Representative immunostaining images for dystrophin and fiber typing in TA muscle cross-sections of *Dmd*$^{mdx\text{-}4Cv}$*; Prkdc*$^{scid}$ mice at 4 weeks post-transplantation with the specified rat-mouse chimera-derived myoblasts. Scale bar, 1mm (top), 100μm (bottom). **(C)** A graph showing maximal twitch force (top), time to peak twitch force (middle) and half relaxation time after twitch contraction (bottom) in TA muscles of *Dmd*$^{mdx\text{-}4Cv}$*; Prkdc*$^{scid}$ mice at 4 weeks post-transplantation with the mouse-mouse (yellow) or rat-mouse (green) chimera-derived myoblasts ("Myobl") compared to PBS control. The results were normalized to muscle weight. Max twitch forces were further normalized to the PBS control group. N=8 mice measured per group, with different dot colors specifying mouse-mouse (yellow) or rat-mouse

(green) chimera-derived myoblast lines used for transplantations. 3 different mouse-mouse and 2 different rat-mouse chimera-derived myoblast lines were used for transplantation. Data is presented as mean±SD. Statistical analysis was performed using a t-test. **(D)** A graph showing the force-frequency relationship in TA muscles of *Dmd*$^{mdx\text{-}4Cv}$; *Prkdc*$^{scid}$ mice at 4 weeks post-transplantation with mouse-mouse and rat-mouse chimera-derived myoblasts vs. PBS control. Results were normalized to the muscle weight and force measured at 150Hz. N=8 animals per group. Data is presented as mean±SD. **(E)** Quantification of dystrophin$^{+}$ myofibers with centrally located nuclei per total number of dystrophin$^{+}$ myofibers in TA muscle cross-section of *Dmd*$^{mdx\text{-}4Cv}$; *Prkdc*$^{scid}$ mice at 4 weeks post-transplantation with the indicated myoblasts. N=4 transplantation recipients per group. Data is presented as mean±SD. Statistical analysis was performed using a t-test. **(F)** Representative images of TA muscle cross-section from *Dmd*$^{mdx\text{-}4Cv}$; *Prkdc*$^{scid}$ mice immunostained for the indicated markers at 4 weeks post-transplantation with the specified cell lines. Arrowheads point to co-localization of PAX7 expression and the *Pax7-nGFP* reporter in rare cells. Scale bar, 100μm (left) and 25μm (right). **(G)** Representative immunostaining images of MYHC in myotubes differentiated from re-isolated myoblasts. Scale bar, 100μm. **(H)** Fusion index analysis of myotubes differentiated from re-isolated myoblasts and a positive myoblast control. N=3 images per myoblast line, 1 myoblast line was used. Data is presented as mean±SD. **(I)** Representative immunostaining images for the indicated skeletal muscle markers in myotubes differentiated from re-isolated myoblasts. Scale bar, 100μm (top), 25μm (bottom).

**Supplemental Video Legends**

**Supplemental video 1:** This movie shows contractility of myotubes obtained through in vitro differentiation of *Pax7-nGFP* mouse-derived myoblasts.

**Supplemental video 2:** This movie shows contractility of myotubes obtained through in vitro differentiation of intraspecies *$Pax7^{Cre/ERT2}$; $Rosa26^{lsl\text{-}DTA}$ / $Dmd^{mdx}$; Pax7-nGFP* chimera-derived myoblasts.

**Supplemental video 3:** This video shows contractility of myotubes obtained through in vitro differentiation of intraspecies *$Pax7^{Cre}$; $Rosa26^{lsl\text{-}DTA}$ / $Dmd^{mdx}$; Pax7-nGFP* chimera-derived myoblasts.

**Supplemental video 4:** This movie shows contractility of myotubes obtained through in vitro differentiation of interspecies rat-mouse chimera-derived myoblasts.

**Supplemental video 5:** This movie shows contractility of myotubes obtained through in vitro differentiation of *Pax7-nGFP* myoblasts that have been re-isolated from the muscles of *$Dmd^{mdx\text{-}4Cv}$; $Prkdc^{scid}$* 4 weeks after intramuscular transplantation with interspecies rat-mouse chimera-derived myoblasts.

## Supplemental Methods

### Animals

The mice and rats used in this study were housed in Allentown cages, under standard conditions at room temperature and a relative humidity of 50-60%, with a 12h light-dark cycle. All animals had ad libitum access to food and water. The following mouse strains from Jackson Laboratory were used: C57BL/10ScSn-*Dmd*$^{mdx}$/J (Stock No: 001801, in this manuscript referred to as *Dmd*$^{mdx}$); B6.129P2-*Gt(ROSA)26Sor*$^{tm1(DTA)Lky}$/J (Stock No 009669, in this manuscript referred to as *Rosa26*$^{lsl\text{-}DTA}$); *B6.Cg-Pax7*$^{tm1(cre/ERT2)Gaka}$/J (Stock No: 017763 in this manuscript referred to as *Pax7*$^{Cre/ERT2}$); *Pax7*$^{tm1(cre)Mrc}$/J (stock No: 010530, in this manuscript referred to as *Pax7*$^{Cre}$) and B10ScSn.Cg-*Prkdc*$^{scid}$ *Dmd*$^{mdx}$/J (stock No: 018018) x B6Ros.Cg-*Dmd*$^{mdx\text{-}4Cv}$/J (stock No: 002378), in this manuscript referred to as *Dmd*$^{mdx\text{-}4Cv}$*; Prkdc*$^{scid}$. The *Tg:Pax7-nGFP/C57BL6;DBA2* mouse strain, a kind gift from Dr. Shahragim Tajbakhsh (1), referred to as *Pax7-nGFP* in this manuscript, was used for production of *Dmd*$^{mdx}$*; Pax7-nGFP* MEFs and as donor for satellite cells and myoblasts in transplantation experiments. In addition, B6 Albino B67Rj-*Tyr*$^{c/c}$ mice (Janvier Labs, France) crossed with B6.129P2-*Gt(ROSA)26Sor*$^{tm1(DTA)Lky}$/J mice were used to produce *Rosa26*$^{lsl\text{-}DTA}$ albino blastocysts. Furthermore, SWISS RjOrl:SWISS (CD-1) mice were used as foster females and vasectomized males (Janvier Labs, France). Finally, Sprague-Dawley RjHan:SD rats were used as rat blastocyst donors, foster females and vasectomized males for rat-mouse chimera production (Janvier Labs, France). The present study was approved by the Federal Food Safety and Veterinary Office, Cantonal veterinary office in Zurich under animal experimental licenses: ZH246/18, ZH177/18, ZH 002/22, ZH032/23 and FormG-135.

### Cell culture

Homozygous *Dmd*$^{mdx}$ female mice were crossed with *Pax7-nGFP* homozygous male mice to obtain *Dmd*$^{mdx}$*; Pax7-nGFP* embryos for MEF derivation. To this end, E13.5 embryos underwent removal of internal organs and heads, followed by thorough mincing and propagation in 'MEF medium' (Table 1) for fibroblast cell line production. HEK-293T cells used for lentiviral generation were grown in 'MEF medium'. Cellular reprogramming to produce and maintain iPSCs was performed in 'MES medium' (Table 1). Both iPSCs and ESCs were cultured on γ-irradiated CF-1 or DR1 MEFs (produced in-house), and cultured in 'Enhanced medium' (Table 1) for 5 days prior to blastocyst injection (2). Myoblasts were cultured in 'Myoblast medium' on Matrigel (CLS354234, Corning) and differentiated in 'Differentiation medium' (Table 1). For myoblast differentiation, $4x10^4$ myoblasts per well were plated on Matrigel-coated 12-well plates in 'Myoblast medium'. On the next day, 'Myoblast medium' was replaced by 'Differentiation medium'. Cells were differentiated for 5-7 days. All cells were passaged using Gibco Trypsin-EDTA (25300054, Thermo Fisher Scientific) and tested for mycoplasma (LT07-318, Lonza). All cells were maintained at 37°C in a 5% $CO_2$ incubator. For detailed media composition, please refer to Supplemental Table 1.

### Lentiviral transduction

To produce lentiviruses, HEK293T cells were cultured in 10cm plates until reaching 90-95% confluency. At this stage, cells were transfected with 770µl of OptiMEM (31985062, Thermo Fisher) mixed with 50µl of TransIT-LT1 Transfection Reagent (MIR 2305, Labforce) and incubated at room temperature for 5min. About 5.5µg of the envelope vector plasmid "VSVG", 8.25µg of the packaging vector plasmid "delta 8.9" and 11µg of the "target" vector plasmid were added together and the mixture was incubated at room temperature for 15min before being added to HEK-293T cells in antibiotic-free medium. To produce lentiviruses overexpressing pluripotency factors under doxycycline (dox) control, we used *FUW-M2rtTA* and *tetO-mSTEMCCA* plasmids as previously reported (3, 4). For RFP labeling of ESCs or iPSCs, viral particles carrying a *pLV[Exp]-Puro-EF1A>TurboRFP(ns):T2A:(H2B-RFP)* transgene (Addgene plasmid ID: 201120) were used as previously reported (5). At 48h post-transfection, viral titers were collected from HEK-293T cells, filtered (0.45µm) and added to medium containing 1:2000 polybrene (TR-1003-G, Sigma-Aldrich) to facilitate efficient transduction. Viral supernatant was then added to the target cells which were at approximately 70% confluency. About 24h later, transduction was repeated with 72h viral titers. On the next day, the medium containing viral particles was washed several times with PBS and replaced with fresh medium. Transduced cells were then expanded to confluency and split prior to experimental use.

**Generation of iPSCs**

About $2x10^4$ MEFs that have been transduced with lentiviruses encoding for *FUW-M2rtTA* and *tetO-mSTEMCCA* were seeded onto one well of a 6-well plate in 'MES medium' supplemented with 2µg/ml dox (D9891-5G, Sigma-Aldrich), 3µM CHIR99021 (4423/50, R&D Systems) and 50µg/ml ascorbic acid (A4403-100MG, Sigma-Aldrich) to initiate reprogramming to pluripotency (4). The medium and small molecules were replaced daily until iPSC colonies appeared in the culture dish. Dox was withdrawn on day 7 and 3 days later dox-independent iPSC colonies were picked, transferred onto γ-irradiated CF-1 MEFs and expanded in 'MES medium' (Table 1).

**Gene editing**

We transfected single-cell suspension of iPSCs for 1h after passaging with *pRP[CRISPR]-EGFP/Puro-hCas9-U6>(long left)-U6>(long right)* plasmid (Addgene plasmid ID: 216871) using Lipofectamine 3000 Transfection Reagent (L3000008, Thermo Fisher Scientific) according to the manufacturer's instructions. At 1-day post-transfection, puromycin (A1113803, Thermo Fisher Scientific) was added at a final concentration of 1µg/ml for 3 days to enable selection for cells that received the plasmid. After recovery in puromycin-free medium, 24 single iPSC colonies were picked, expanded and characterized. Of these, 2 iPSC clones showed the expected editing of the dystrophin gene, with an additional 6bp of intronic deletion that did not impact the skipping of the mutant exon.

**Alkaline phosphatase staining**

Testing for alkaline phosphatase activity was performed using the Leukocyte Alkaline Phosphatase Kit (86R-1KT, Sigma-Aldrich) per the manufacturer's protocol.

**Differentiation of iPSCs into myogenic cells**

A previously reported iPSC differentiation protocol towards the myogenic lineage was performed as described by the authors (6, 7). We used the protocol tailored to mouse cells which contained serum in the medium.

**Karyotyping**

KaryoMAX Colcemid Solution (15212012, Thermo Fisher Scientific) was added to the iPSCs at a final concentration of 100ng/ml. After a 5 hour incubation at 37°C, cells were harvested, centrifuged, and the supernatant was removed to about 1ml. Cells were then re-suspended by vortexing at low setting and 5ml of pre-warmed hypotonic solution (0.56g KCl+0.5g sodium citrate in 200ml $H_2O$) was added dropwise while mixing. The cell suspension was then incubated at 37°C and after 30min, 2.5ml of fixative (Methanol:Acetic acid = 3:1) was added. The mixture was centrifuged, and following supernatant removal, the cell pellet was re-suspended in 2ml of fixative while vortexing followed by incubation at room temperature for 5min. This step was repeated 3 times. Next, the cell pellet was re-suspended in 1ml of fixative and 400µl of the cell suspension was pipetted single-drop-wise onto a polarized microscope slide from a height of about 150cm. After the slides dried, they were immersed in Giemsa solution (T862.1, Carl Roth) for 7min, added into Gurr's buffer (0.469g $NaH_2PO_4$ + 0.937g $Na_2HPO_4$ in 1l of $H_2O$) for 2min and then washed with water. Slides were allowed to dry prior to examination. For analysis, 30 cell spreads were randomly selected for chromosome counting.

**Blastocyst and morulae injections**

Blastocyst injections and embryo transfer were performed in-house, abiding to legal rules of the Federal Food Safety and Veterinary Office, Cantonal veterinary office (Zurich) and animal experimental licenses: ZH246/18, ZH032/23 and FormG-135.

For mouse intraspecies blastocyst injections, mice between the age of 3-6 weeks were superovulated via intraperitoneal injection of 5IU PMSG (HOR-272, ProSpec). About 46-48h later, the mice were injected again with 5IU hCG (HOR-250, ProSpec) to induce ovulation, and paired with stud males. The following morning, the females were separated and euthanized after 48 hours. The morulae were flushed from the oviduct using M2 Medium (M7167, Sigma-Aldrich) and incubated overnight at 37°C and 5% $CO_2$. On the injection day, cells were pre-plated and kept on ice until injection. Typically, 8-12 iPSCs or ESCs were injected per blastocyst. Successfully injected mouse blastocysts were transferred to a 60mm center-well-organ culture dish containing EmbryoMax KSOM medium (MR-106-D, Sigma-Aldrich) and kept in an incubator at 37°C and 5% $CO_2$ for 1-2h.

The injected blastocysts were then transferred to the uteri of 2.5dpc pseudo-pregnant female mice. For generation of *Pax7*$^{Cre/ERT2}$*; Rosa26*$^{lsl\text{-}DTA}$ blastocysts, male and female mice homozygous for both alleles were utilized. For generation of *Pax7*$^{Cre}$*; Rosa26*$^{lsl\text{-}DTA}$ blastocysts, superovulated and homozygous B6.129P2-*Gt(ROSA)26Sor*$^{tm1(DTA)Lky}$/J females were paired with homozygous *Pax7*$^{tm1(cre)Mrc}$/J males.

For mouse-to-rat interspecies morulae injections, SD rats (aged 8-15 weeks) were synchronized via injection of 40µg of LHRH (L4513, Sigma-Aldrich). About 94-96h later, estrous cycle was checked using Rat Vaginal Impedance Checker (MK-12, Muromachi Kikai Co., Ltd.) and the rats were paired with stud males. After 90-94h, the morulae were flushed in M2 medium. Injections were carried out in droplets of M2 medium covered with Mineral oil (M8410-1L, Sigma-Aldrich). We chose to inject cells into morulae because injected blastocysts oftentimes did not yield pups. On the injection day, cells were pre-plated and kept on ice until injection. Typically, 8-12 iPSCs were injected per morula. Successfully injected morulae were transferred onto a 60mm center well organ culture dish containing rat KSOM (CSR-R-R148, Cosmo Bio) and kept in an incubator at 37°C and 5% $CO_2$ for 1-2h. The injected morulae were then transferred to the uteri of 2.5dpc pseudo-pregnant female SD rats.

Injections were performed with the Olympus fully-automated microinjection system IXplore Pro combined with a Narishige MTK-1 hydraulic micromanipulator (Narishige), a CellTram oil and a PiezoXpert (both from Eppendorf). When applicable, the presence of chimerism in intraspecies and interspecies chimeras was first assessed using visual examination of coat color. Chimerism was further assessed by DNA genotyping for the RFP or *Pax7-nGFP* transgenes carried by donor ESCs/iPSCs. The number of animals and embryos used for production of chimeras can be found in Supplemental Tables 2-6.

**Postnatal satellite cell ablation**

A tamoxifen-inducible '*Pax7*$^{Cre/ERT2}$*; Rosa26*$^{lsl\text{-}DTA}$' system was used to ablate host satellite cells during postnatal growth. Mice received an intraperitoneal injection of 50µl of 1mg/ml tamoxifen (T5648-1G, Sigma-Aldrich) in corn oil (C8267, Sigma-Aldrich) every day between postnatal days 3 and 5 followed by bi-weekly injections of 75mg tamoxifen/kg bodyweight until analysis.

**Satellite cell isolation**

A Sony SH800S Cell Sorter was utilized for all FACS analyses and purifications reported in this study. For satellite cell isolation, skeletal muscles were harvested, minced and centrifuged in PBS at 350g for 3min. The tissue pellet was then resuspended in a digestion solution containing 2mg/ml Collagenase Type II (17101015, Thermo Fisher Scientific) in DMEM (41966029, Thermo Fisher Scientific) and incubated for 90min. This step was followed by a 30min incubation in a digestion solution consisting of F-10 (22390025, Thermo Fisher Scientific) supplemented with 10% horse serum (16050122, Thermo Fisher Scientific), 5.5% of DMEM (41966029, Thermo Fisher Scientific) containing 0.2% Collagenase Type II (17101015, Thermo Fisher Scientific) and 7% of PBS supplemented with 0.4% Dispase II (Thermo Fisher Scientific, 17105041). Both incubation steps were performed in a shaking 37°C water bath (VWR 10128-126). An 18-gauge needle syringe was then used to dislodge the cells from the fibers, followed by filtering the cells with 100µm, 70µm and 40µm cell strainers, respectively. The cell pellet was then resuspended in 'FACS buffer' consisting of PBS supplemented with 2% FBS (10270106, Thermo Fisher Scientific) and kept on ice until sorting. Satellite cells were FACS-purified using either the *Pax7-nGFP* reporter or the following combinations of cell surface markers: APC anti-mouse lymphocyte antigen 6 family member A, LY6A (SCA1) antibody (108111, BioLegend), mouse Integrin alpha 7 (ITGA7) Alexa Fluor 750-conjugated antibody (FAB3518S, R&D Systems), APC anti-mouse protein tyrosine phosphatase receptor type C antibody (PTPRC, CD45) (103111, BioLegend) and APC anti-mouse platelet/endothelial cell adhesion molecule 1 antibody (PECAM1/CD31) (102409, BioLegend). In each FACS experiment, DAPI (62248, Thermo Fisher Scientific) was used at 1:1000 dilution to exclude dead cells. The EGFP channel was used for FACS-purification of *Pax7*-nGFP cells. For FACS-purification using cell surface markers, all SCA1$^{+}$, CD45$^{+}$ and CD31$^{+}$ cells were excluded using the APC channel. The ITGA7$^{+}$ cells were then sorted from the remaining population and analyzed for expression of the *Pax7-nGFP* reporter.

**Intramuscular transplantation of myoblasts and satellite cells**

*Pax7-nGFP* mice, intraspecies and interspecies chimeras were used for satellite cell isolation between 5-35 weeks of age and were sex- and age-matched as closely as possible. Recipient mice (*Dmd*$^{mdx\text{-}4Cv}$*; Prkdc*$^{scid}$) were of both sexes, between 12 and 41 weeks of age, and appropriately sex- and age-matched for experiments. Prior to transplantation, *Dmd*$^{mdx\text{-}4Cv}$*; Prkdc*$^{scid}$ mice received an injection of 50µl of 10µM Cardiotoxin (L8102, Latoxan) into the TA muscles to enhance cell engraftment. One day post-injury, a cell suspension was injected craniocaudally into one pre-injured TA muscle using an insulin syringe (324824, BD). As a control, PBS was injected into the contralateral pre-injured TA muscle, unless otherwise indicated. Notably, for force measurement analysis post-transplantation, PBS was injected into TA muscles of mice that were not subjected to cell transplantation. In general, mice were euthanized and TA muscles were harvested 4 weeks after cell transplantation. For satellite cell transplantation, cells were centrifuged at 500g for 5min immediately after FACS-purification and resuspended in 20µl PBS until injection. Unless otherwise indicated, 1 million myoblasts have been used for intramuscular transplantations. CTX injury and intramuscular transplantation were performed under isoflurane inhalation anesthesia.

**Force measurements**

Animals were anesthetized using Ketamine and Xylazine for the duration of the experiment prior to euthanasia and recovery of muscles. Once fully anesthetized (i.e. loss of pedal withdrawal reflex), the distal tendon of the right TA muscle was surgically detached from the bone and the sciatic nerve was exposed. Mineral oil (8042-47-5. Sigma-Aldrich) was used to prevent the open tissues from drying. The mouse was then placed onto a heating pad of the 809C in-situ Mouse Apparatus (Aurora Scientific), connected to the 300C muscle Lever (Aurora Scientific) and fixed according to the manufacturer's instructions. Briefly, the knee was firmly fixed with a needle, the foot was fixed with a clamp and the TA distal tendon was connected to the lever arm of the force transducer using a silk suture. Electrodes were placed below the exposed sciatic nerve. Several twitch contractions at 1Hz were performed to determine optimal muscle length. Next, force-frequency relationship was assessed. To this end, the muscle was stimulated at 1Hz, 10Hz, 20Hz, 30Hz, 50Hz, 75Hz, 100Hz, 150Hz, 200Hz and 250Hz and active force during each contraction was determined. Between each stimulation at frequencies 1Hz-100Hz, there was a 30s rest period. Between contractions induced at frequencies higher than 100Hz, there was a 1min resting period. After the last contraction at 250Hz, a 1min resting period was taken, and a second 1Hz twitch contraction was induced. Maximum twitch force was determined as the highest force measured at 1Hz. Time to peak and half relaxation times were determined from the same twitch contraction. At 1min after the second 1Hz contraction, a fatigue protocol was started to determine the reduction in the muscle force after continuous tetanic contractions. In this protocol, 350ms tetanic contractions at 100Hz were induced every 3s over a period of 4.5min. Active force was determined for each contraction. At the end of the protocol, the animal was removed from the machine, euthanized, and the TA muscles were carefully dissected and immediately weighed. For the data analysis, muscle force was normalized to the TA weight. Data for the fatigue curve was further normalized to the force measured at 100Hz during the determination of the force-frequency curve. Maximal twitch forces were further normalized to the average maximal twitch force of the PBS group. Data for the force-frequency curve is expressed relative to the force generated at 150Hz.

**Muscle embedding and processing**

Harvested TA muscles were placed onto a cork covered with 10% Tragacanth (G1128, Sigma-Aldrich), and frozen for 30-60s in 2-Methylbutane (3927.1, Carl Roth) pre-cooled in liquid nitrogen. The samples were added to liquid nitrogen for 1min and then stored at -80°C. Frozen muscles were cryo-sectioned into 10µm thick muscle cross-sections using a cryostat (Leica CM1950) and stored at -80°C.

**Genomic DNA isolation and PCR of DNA**

Isolation of genomic DNA from cells was performed using the DNeasy Blood & Tissue Kit (69504, Qiagen) according to the manufacturer's instruction. DNA for PCR from muscle lysates was obtained by direct lysis of muscle slurry generated by mincing and followed by 90min incubation in digestion solution containing 2mg/ml Collagenase Type II (17101015, Thermo Fisher Scientific) in DMEM (41966029, Thermo Fisher Scientific) at 37°C using DirectPCR Lysis Reagent (mouse tail) (VIG102-

T, Viagen Biotech). All PCR reactions were performed using GoTaq G2 Hot Start Green Master Mix (M7423, Promega). PCR for the RFP transgene and *Rosa26-DTA* allele was performed with primers mRFP1.F/mRFP1.R and DTA.F/DTA.R via the following program: 94°C 5min, 30x (94°C 30s, 60°C (RFP) or 55°C (DTA) 1min, 72°C 30s), 72°C 5min. The PCR for the *Pax7-nGFP* transgene was performed using primers Pax7nGFP.F/Pax7nGFP.R via the following program: 94°C 2min, 30x (94°C 30s, 65°C 30s, 72°C 30s), 72°C 5min. Gene editing of mouse dystrophin was verified using primers Dmd_i22-i23.F/Dmd_i22-i23.R or primers Dmd-i22-i23.F2/Dmd-i22-i23.R2 using the following program: 94°C 5min, 30x (94°C 30s, 55°C 30s, 72°C 30s), 72°C 5min. Rat dystrophin was detected using primers Rat Dmd i22-i23.F/Rat Dmd i22-i23.R via the following program: 94°C 5min, 30x (94°C 30s, 57°C 1min, 72°C 45s), 72°C 5min. PCR products were separated on a 1.5-2% agarose gel (7-01P02-R, BioConcept) dissolved in TAE buffer (3-07F03-I, BioConcept) and visualized using GelRed Nucleic Acid Stain (Cat# 41003, Biotium). All primers were synthetized by Microsynth (Balgach, Switzerland). For primer sequences please refer to Supplemental Table 7.

**RNA Extraction and cDNA synthesis**
RNA isolation was performed using the Qiagen's RNeasy kit, with 15min DNase digest (74104, Qiagen) according to the manufacturer's protocol. The concentration was measured by a Tecan Spark 10M. The cDNA was synthesized using the High-Capacity cDNA Reverse Transcription Kit (4368814, Thermo Fisher Scientific) utilizing 1µg of RNA according to the manufacturer's instructions.

**PCR and qPCR of cDNA**
To assess gene editing of dystrophin at the mRNA/cDNA level, RT-PCR was performed with primers Dmd_e22-e24.F/Dmd_e22-e24.R using GoTaq G2 Hot Start Green Master Mix (M7423, Promega) according to the manufacturer's instructions via the following program: 94°C 5min, 35x (94°C 30s, 55°C 30s, 72°C 30s), 72°C 5min. For primer sequences please refer to Supplemental table 7. PCR products were run on a 1.5-2% agarose gel (7-01P02-R, BioConcept) in TAE buffer (3-07F03-I, BioConcept) and imaged using GelRed Nucleic Acid Stain (41003, Biotium). Probe-based qRT-PCR for myogenic markers was performed using the PrimeTime Gene Expression Master Mix (1055771, IDT). 10ng of cDNA was used per each qPCR reaction. All reactions were performed in triplicates using the QuantStudio5 qPCR machine (A34322, Thermo Fisher Scientific). Data was analyzed using the $2^{-\Delta\Delta Ct}$ method, with *Gapdh* serving as a housekeeping control gene. For probe and primer sequences please refer to Supplemental Table 8.

**Sanger Sequencing**
PCR products for dystrophin (DNA- or cDNA-derived) were run on a 1.5-2% agarose gel (7-01P02-R, BioConcept) in TAE buffer (3-07F03-I, BioConcept). Products were extracted from the gel using the QIAquick Gel Extraction Kit (28706, Qiagen) and Sanger sequenced by Microsynth (Balgach, Switzerland). Results were evaluated with SnapGene Viewer 3. The following primers were used for sequencing: Dmd_e22-e24.F (for sequencing of PCR products obtained from cDNA), Dmd_i22-i23.F (for sequencing of PCR products obtained from genomic DNA). For primer sequences please refer to Supplemental Table 7.

**Immunostaining**
Cells cultured in 6-well plates were washed with PBS and fixed with 4% Paraformaldehyde (11400580, Fisher Scientific) in PBS for 5min at room temperature (RT). After two PBS washing steps, 'blocking solution' composed of PBS supplemented with 2% BSA (A1391, AppliChem) and 1% Triton X-100 (9002-93-1, Sigma-Aldrich) was added to the fixed cells for 30min at RT. Primary antibodies diluted in 'blocking solution' containing 0.2-1% Triton-X-100 were added for 1h at RT. Cells were then washed twice with PBS and incubated with secondary antibodies and DAPI (62248, Thermo Fisher Scientific) in 'blocking solution' for 1h. Cells were washed twice with PBS and covered with ProLong Gold Antifade Mountant (P36934, Thermo Fisher Scientific) to prevent photobleaching.

For DYSTROPHIN/DAPI, PAX7/LAMA/DAPI and DYSTROPHIN/PAX7/GFP/DAPI staining, TA muscle sections on microscopy slides (J1800AMNZ, Epredia) were fixed with 4% Paraformaldehyde (11400580, Thermo Fisher Scientific) in PBS for 5min and washed twice with PBS. 'Blocking solution' consisting of PBS supplemented with 1% BSA (A1391, AppliChem) and 0.2% Triton X-100 (9002-93-1, Sigma-Aldrich) was added for 15min. Primary antibodies diluted in

‘blocking solution’ were added for 1h at RT, after which the sections were washed twice with PBS and incubated for 30-60min with secondary antibodies and DAPI (62248, Thermo Fisher Scientific) diluted in ‘blocking solution’. Finally, the sections were washed twice with PBS and covered with a few drops of ProLong Glass Antifade Mountant (P36980, Thermo Fisher Scientific) and a coverslip.

For muscle fiber typing, TA muscle cross-sections on microscopy slides were permeabilized with 0.5% Trition-X-100 (9002-93-1, Sigma-Aldrich) in PBS for 10min, washed in PBS for 5min and then blocked with PBS supplemented with 10% goat serum (G9023, Sigma-Aldrich) for 1h at RT. Primary antibodies diluted in PBS supplemented with 10% goat serum (G9023, Sigma-Aldrich) were added for 2h at RT. Slides were then washed in PBS 3 times for 5min and incubated in a mixture of appropriate secondary antibodies and DAPI diluted in PBS supplemented with 10% goat serum (G9023, Sigma-Aldrich) for 1h. Sections were washed 3 times with PBS for 5min and covered with a few drops of ProLong Glass Antifade Mountant (P36980, Thermo Fisher Scientific) and a coverslip. For more information about the antibodies used including their source and dilution please refer to Supplemental Tables 9 and 10.

**Microscopy and image processing**

All microscopy images were taken with a Nikon ECLIPSE Ti2 microscope. For the assessment of background signal, cells stained only with secondary antibodies were used. Look-up tables (LUTs) determining fluorescence intensities for each fluorophore and other settings were kept the same for all samples in each experiment unless stated otherwise in the figure legends. Fusion index was determined as the percentage of MYHC$^{+}$/DAPI$^{+}$ cells per total number of DAPI$^{+}$ cells. 3 images were quantified per each myoblast line. For quantification of dystrophin$^{+}$ fibers and area in TA muscle cross-sections, those with the highest dystrophin content were used from each muscle across all samples. In all presented restored dystrophin fibers/area quantifications, each dot represents quantification from one recipient mouse. PBS controls correspond to the quantification of dystrophin+ area/revertant fibers in the contralateral legs of the same recipient mice. Surface measurements of dystrophin$^{+}$ area on muscle cross-sections were performed using the microscope’s software. The number of dystrophin$^{+}$ fibers in each section was counted using ImageJ software’s ‘Multipoint’ function.

**Bulk RNA sequencing**

RNA was isolated from cells using a Qiagen RNeasy kit, with 15min DNase digest (74104, Qiagen) according to the manufacturer’s protocol. RNA sequencing was performed at the FGCZ on an Illumina NovaSeq instrument and library was prepared according to Illumina’s TruSeq mRNA protocol. The sequencing reads were analyzed using the SUSHI framework (8, 9) developed at the FGCZ. After the quality control (i.e. adapter and low-quality base trimming) with fastp v0.20 (10), raw reads were pseudo-aligned against the reference mouse genome assembly GRCm39 and gene expression level (GENCODE release 26) was quantified using Kallisto v0.46.1 (11). Genes were considered to be detected if they had at least 10 counts in 50% of the samples. *Pax7-nGFP* MEFs and *Pax7-nGFP* myoblast samples from the GEO dataset GSE169053 were re-processed as previously done (12). TPM (Transcripts per million mapped reads) was used as the unit for normalized gene expression.

**Single cell RNA sequencing**

Animals were euthanized and skeletal muscle tissue was collected from the whole body. Around 2-3g of the tissue was minced, washed and centrifuged at 350g for 5min. The pellet was re-suspended and incubated in HBSS (14025050, Thermo Fisher Scientific) supplemented with 1.5% BSA (A1391, AppliChem) and 0.2% Collagenase II (17101015, Thermo Fisher Scientific) at 37°C for 90min. One volume of low glucose DMEM (31885049, Thermo Fisher Scientific) supplemented with 10% FBS (10270106, Thermo Fisher Scientific) was added to the sample. After vortexing, the sample was filtered through a 100μm cell strainer and centrifuged at 350g for 5min. The pellet was re-suspended in 2ml of ACK lysis buffer (A1049201, Thermo Fisher Scientific), kept on ice for 1min, topped with approximately 10 volumes of cold PBS, vortexed and filtered using a 40μm cell strainer. Following 8min centrifugation at 650g, the pellet was re-suspended in cold PBS supplemented with 0.5% BSA (A1391, AppliChem), transferred to a FACS tube and centrifuged at 450g for 5min. The pellet was re-suspended in PBS supplemented with 0.5% BSA (A1391, AppliChem) and Calcein Violet 450 AM Viability Dye (65-0854-39, Thermo Fisher Scientific) was added to the sample at a 1:10,000 dilution.

After 3min incubation, live cells were sorted based on Calcein fluorescence using FACS. The sorted cells were centrifuged at 650g for 5min and the pellet was re-suspended in PBS supplemented with 0.5% BSA (A1391, AppliChem). Around 20,000 live cells were counted using Neubauer's chamber and used for library preparation. Libraries were prepared according to the manufacturer's Chromium Next GEM Single Cell 3' Reagent Kits v3.1 protocol using the 10X Genomics platform. Libraries were processed on an Illumina NovaSeq 6000 SP Flow Cell, with parameters set according to the 10x Genomics recommendations. Paired-end reads with the following specifications were used: R1=28, i7=10, i5=10, R2=90. Sequencing achieved an average depth of around 50,000 reads per cell. Bioinformatic analysis of the sequencing data was performed as previously described (5). Clusters were annotated based on established markers (13-17).

**Supplemental Table 1: Media compositions**

| Medium | Composition |
|---|---|
| MEF medium | DMEM (41966029, Thermo Fisher Scientific) |
| | 10% FBS (10270106, Thermo Fisher Scientific) |
| | 1% MEM Non-Essential Amino Acids Solution (100X) (11140050, Thermo Fisher Scientific) |
| | 1% Penicillin-Streptomycin (10,000 U/mL) (15140122, Thermo Fisher Scientific) |
| | 0.1% Gibco 2-Mercaptoethanol (21985023, Thermo Fisher Scientific). |
| MES medium | DMEM (41966029, Thermo Fisher Scientific) |
| | 1% GlutaMAX Supplement (35050061, Thermo Fisher Scientific) |
| | 1% Penicillin-Streptomycin (10,000 U/mL) (15140122, Thermo Fisher Scientific) |
| | 1% MEM Non-Essential Amino Acids Solution (100X) (11140050, Thermo Fisher Scientific) |
| | 0.1% Gibco 2-Mercaptoethanol (21985023, Thermo Fisher Scientific) |
| | 15 % FBS (10270106, Thermo Fisher Scientific) |
| | 1000U/ml mLIF (#PG-A1140-0010, PolyGene Transgenetics) |
| 'Enhanced' medium | MES medium |
| | 0.5µmol/l VPA (P4543-10G, Sigma-Aldrich) |
| | 1.5µmol/l CGP77675 (SML0314, Merck ) |
| | 3µmol/l CHIR99021 (4423, Tocris Bioscience) |
| Myoblast medium | 50% DMEM (41966029, Thermo Fisher Scientific) |
| | 50% F-10 medium (22390025, Thermo Fisher Scientific) |
| | 10% Horse serum (16050122, Thermo Fisher Scientific) |
| | 20% FBS (10270106, Thermo Fisher Scientific) |
| | 1% Penicillin-Streptomycin (10,000 U/mL) (15140122, Thermo Fisher Scientific) |
| | 10ng/ml basic FGF (233-FB-500, R&D Systems) |
| Differentiation medium | DMEM (41966029, Thermo Fisher Scientific) |
| | 2% Horse serum (16050122, Thermo Fisher Scientific) |
| | 1% Penicillin-Streptomycin (10,000 U/mL) (15140122, Thermo Fisher Scientific). |

**Supplemental Table 2: Number of animals and embryos used to produce *Pax7$^{Cre/ERT2}$; Rosa26$^{lsl\text{-}DTA}$* / RFP$^{+}$KH2-mESC chimeras**

| Injection round | # of blastocyst donors | # of flushed embryos | # of transferred embryos | # of fosters | Total pups alive more than one week after birth | Chimeras |
|---|---|---|---|---|---|---|
| 1 | 15 | 80 | 34 | 3 | 6 | 0 |
| 2 | 15 | 130 | 72 | 4 | 19 | 17 |
| 3 | 15 | 105 | 63 | 4 | 33 | 11 |

**Supplemental Table 3: Number of animals and embryos used to produce *Pax7$^{Cre/ERT2}$; Rosa26$^{lsl-DTA}$ / Dmd$^{mdx}$; Pax7-nGFP* iPSC chimeras**

| Injection round | # of blastocyst donors | # of flushed embryos | # of transferred embryos | # of fosters | Total pups alive more than one week after birth | Chimeras |
|---|---|---|---|---|---|---|
| 1 | 15 | 60 | 46 | 6 | 19 | 10 |
| 2 | 15 | 38 | 36 | 4 | 17 | 11 |

**Supplemental Table 4: Number of animals and embryos used to produce *Rosa26$^{lsl\text{-}DTA}$; Rosa26$^{lsl\text{-}DTA}$ / Dmd$^{mdx}$; Pax7-nGFP* iPSC chimeras**

| Injection round | # of blastocyst donors | # of flushed embryos | # of transferred embryos | # of fosters | Total pups alive more than one week after birth | Chimeras |
|---|---|---|---|---|---|---|
| 1 | 7 | 230 | 20 | 2 | 5 | 4 |

**Supplemental Table 5: Number of animals and embryos used to produce *Pax7$^{Cre}$; Rosa26$^{lsl\text{-}DTA}$ / Dmd$^{mdx}$; Pax7-nGFP* iPSC chimeras**

| Injection round | # of blastocyst donors | # of flushed embryos | # of transferred embryos | # of fosters | Total pups alive more than oneweek after birth | Chimeras |
|---|---|---|---|---|---|---|
| 1 | 5 | 60 | 30 | 3 | 5 | 5 |
| 2 | 7 | 62 | 20 | 2 | 6 | 6 |

**Supplemental Table 6: Number of animals and embryos used to produce interspecies chimeras**

| Injection round | # of blastocyst donors | # of flushed embryos | # of transferred embryos | # of foster females | Total pups alive more than one week after birth | Chimeras |
|---|---|---|---|---|---|---|
| 1 | 2 | 11 | 11 | 3 | 3 | 2 |
| 2 | 1 | 8 | 8 | 2 | 1 | 0 |
| 3 | 5 | 26 | 26 | 3 | 2 | 1 |
| 3 | 3 | 25 | 9 | 3 | 1 | 1 |
| 4 | 3 | 22 | 14 | 3 | 0 | 0 |
| 5 | 3 | 15 | 14 | 3 | 0 | 0 |
| 6 | 3 | 12 | 12 | 3 | 0 | 0 |
| 7 | 2 | 3 | 2 | 1 | 8 | 1 |
| 8 | 5 | 22 | 22 | 4 | 2 | 0 |
| 14 | 6 | 34 | 34 | 3 | 4 | 1 |
| 15 | 6 | 32 | 32 | 3 | 4 | 1 |
| 16 | 6 | 42 | 42 | 4 | 0 | 0 |

**Supplemental Table 7: Primers used for PCR**

| Target | Primer name | Primer sequence (5'→3') |
|---|---|---|
| RFP | mRFP1.F | CCCCGTAATGCAGAAGAAGA |
| | mRFP1.R | CTTGGCCATGTAGGTGGTCT |
| Rosa26-DTA | DTA.F | CGACCTGCAGGTCCTCG |
| | DTA.R | CTCGAGTTTGTCCAATTATGTCAC |
| GFP | Pax7-nGFP.F | CCACACCTCCCCCTGAACCTGAAACATAAA |
| | Pax7-nGFP.R | GAATTCCCCGGGGAGTCGCATCCTGCGG |
| Mouse dystrophin (DNA) | Dmd_i22-i23.F | TGAAACTCATCAAATATGCGTGT |
| | Dmd_i22-i23.R | TCTGTTTCCCATCACATTTTCCA |
| Mouse dystrophin (DNA) | Dmd_i22-i23.F2 | TGC GTGTTAGTGTAAATGAACTT |
| | Dmd_i22-i23.R2 | TCTTTGAAGGACTCTGGGTAAAA |
| Mouse dystrophin (cDNA) | Dmd_e22-e24.F | CACTTTACCACCAATGCGCT |
| | Dmd_e22-e24.R | ACATCAACTTCAGCCATCCA |
| Rat dystrophin (DNA) | Rat Dmd i22-i23.F | AGAAAACTCCTGTGATGTGAGG |
| | Rat Dmd i22-i23.R | ACATAGGACAAATAGGCGAGTT |

**Supplemental Table 8: Primers and probes used for qPCR**

| Target | Forward primer (F), reverse primer (R) and probe (P) sequences (5'→3') | Manufacturer |
|---|---|---|
| mGapdh<br>F+R+P | F: GTGGAGTCATACTGGAACATGTAG<br>R: AATGGTGAAGGTCGGTGTG<br>P:_/56-FAM/TGCAAATGG/ZEN/CAGCCCTGGTG/3IABkFQ/ | IDT Assay Mm.PT.39a.1 |
| mMyf5<br>F+R+P | F: ACATGCATTTGATACATCAGGAC<br>R: CACCTCCAACTGCTCTGAC<br>P:/56-FAM/TGCCTGAAT/ZEN/GTAACAGCCCTGTCTG/3IABkFQ/ | IDT Assay Mm.PT.58.5271235 |
| mMyod1<br>F+R+P | F: GACACAGCCGCACTCTT<br>R: GCTCTGATGGCATGATGGAT<br>P: /56-FAM/ACGACACCG/ZEN/CCTACTACAGTGAGG/3IABkFQ/ | IDT Assay Mm.PT.58.8193525 |
| mPax7<br>F+R+P | F: GAAGAAGTCCCAGCACAGC<br>R: GCTACCAGTACAGCCAGTATG<br>P:/56-FAM/CCAAAAACG/ZEN/TGAGCCTGTCCACAC/3IABkFQ/ | IDT Assay Mm.PT.58.12398641 |

**Supplemental Table 9: Primary antibodies used for immunostaining**

| Antibody | Isotype | Catalog number and manufacturer | Dilution |
|---|---|---|---|
| Mouse anti-mouse MYHC | IgG2b | MAB4470, R&D Systems | 1:500 |
| Mouse anti-mouse OCT4 | IgG1 | MA1104, Thermo Fisher Scientific | 1:200 |
| Mouse anti-mouse PAX7 | IgG1 | MAB1675, R&D Systems | 1:100 |
| Rabbit anti-mouse Dystrophin | IgG | ab15277, Abcam | 1:200 |
| Rabbit anti-mouse Laminin | IgG | ab11575, Abcam | 1:100 |
| Rabbit anti-mouse SOX2 | IgG1 | 481400, Thermo Fisher Scientific | 1:200 |
| Rat anti-mouse NANOG conjugated eFluor 660 | IgG2a, κ | 50576182, Thermo Fisher Scientific | 1:100 |
| Mouse anti-mouse ACTN1 (sarcomeric) | IgG1 | A7811, Sigma-Aldrich | 1:750 |
| Mouse anti-bovine TTN | IgM | 9 D10, DSHB | 1:100 |
| Mouse anti-GFP | IgG2a | A11120, Thermo Fisher Scientific | 1:100 |
| Mouse anti-mouse Myosin heavy chain (slow, alpha- and beta-) (Type I) | IgG2b | BA-F8, DSHB | 1:50 |
| Mouse anti-mouse Myosin heavy chain Type IIA | IgG1 | SC-71, DSHB | 1:200 |
| Mouse anti mouse MYH4 (Type IIB) | IgM | BF-F3, DSHB | 1:100 |

**Supplemental Table 10: Secondary antibodies used for immunostaining**

| Antibody | Catalog number and manufacturer | Dilution |
|---|---|---|
| Goat anti-mouse IgG1 350 | A21120, Thermo Fisher Scientific | 1:250 |
| Goat anti-mouse IgG1 647 | A21240, Thermo Fisher Scientific | 1:400 |
| Donkey anti-rabbit IgG 647 | A31573, Thermo Fisher Scientific | 1:250 (fibre typing) or 1:400 (other stainings) |
| Goat anti-rabbit IgG 488 | A11008, Thermo Fisher Scientific | 1:400 |
| Goat anti-rabbit IgG 546 | A11010, Thermo Fisher Scientific | 1:400 |
| Goat anti-mouse IgG2b 546 | A21143, Thermo Fisher Scientific | 1:400 |
| Goat anti-mouse IgG2b 647 | A21242, Thermo Fisher Scientific | 1:400 |
| Goat anti-mouse IgG2b 488 | A21141, Thermo Fisher Scientific | 1:250 |
| Goat anti-mouse IgG2a 488 | A21131, Thermo Fisher Scientific | 1:400 |
| Goat anti-mouse IgM 546 | A21045, Thermo Fisher Scientific | 1:250 |

**References**


1. Sambasivan R, Gayraud-Morel B, Dumas G, Cimper C, Paisant S, Kelly RG, et al. Distinct regulatory cascades govern extraocular and pharyngeal arch muscle progenitor cell fates. *Dev Cell.* 2009;16(6):810-21.
2. Mori M, Furuhashi K, Danielsson JA, Hirata Y, Kakiuchi M, Lin CS, et al. Generation of functional lungs via conditional blastocyst complementation using pluripotent stem cells. *Nat Med.* 2019;25(11):1691-8.
3. Sommer CA, Stadtfeld M, Murphy GJ, Hochedlinger K, Kotton DN, and Mostoslavsky G. Induced pluripotent stem cell generation using a single lentiviral stem cell cassette. *Stem Cells.* 2009;27(3):543-9.
4. Bar-Nur O, Brumbaugh J, Verheul C, Apostolou E, Pruteanu-Malinici I, Walsh RM, et al. Small molecules facilitate rapid and synchronous iPSC generation. *Nat Methods.* 2014;11(11):1170-6.
5. Zvick J, Tarnowska-Sengul M, Ghosh A, Bundschuh N, Gjonlleshaj P, Hinte LC, et al. Exclusive generation of rat spermatozoa in sterile mice utilizing blastocyst complementation with pluripotent stem cells. *Stem Cell Reports.* 2022.
6. Chal J, Oginuma M, Al Tanoury Z, Gobert B, Sumara O, Hick A, et al. Differentiation of pluripotent stem cells to muscle fiber to model Duchenne muscular dystrophy. *Nat Biotechnol.* 2015;33(9):962-9.
7. Chal J, Al Tanoury Z, Oginuma M, Moncuquet P, Gobert B, Miyanari A, et al. Recapitulating early development of mouse musculoskeletal precursors of the paraxial mesoderm in vitro. *Development.* 2018;145(6).
8. Hatakeyama M, Opitz L, Russo G, Qi W, Schlapbach R, and Rehrauer H. SUSHI: an exquisite recipe for fully documented, reproducible and reusable NGS data analysis. *BMC Bioinformatics.* 2016;17(1):228.
9. Qi W, Schlapbach R, and Rehrauer H. RNA-Seq Data Analysis: From Raw Data Quality Control to Differential Expression Analysis. *Methods Mol Biol.* 2017;1669:295-307.
10. Chen S, Zhou Y, Chen Y, and Gu J. fastp: an ultra-fast all-in-one FASTQ preprocessor. *Bioinformatics.* 2018;34(17):i884-i90.
11. Bray NL, Pimentel H, Melsted P, and Pachter L. Near-optimal probabilistic RNA-seq quantification. *Nat Biotechnol.* 2016;34(5):525-7.
12. Kim I, Ghosh A, Bundschuh N, Hinte L, Petrosyan E, von Meyenn F, et al. Integrative molecular roadmap for direct conversion of fibroblasts into myocytes and myogenic progenitor cells. *Sci Adv.* 2022;8(14):eabj4928.
13. Han X, Wang R, Zhou Y, et al. Mapping the Mouse Cell Atlas by Microwell-Seq. *Cell.* 2018; 172(5):1091-1107.e7.
14. Wang R, Zhang P, Wang J, et al. Construction of a cross-species cell landscape at single-cell level. *Nucleic Acids Res*. 2023;51(2):501-516.
15. Franzén O, Gan LM, Björkegren JLM. PanglaoDB: A web server for exploration of mouse and human single-cell RNA sequencing data. *Database*. 2019;2019(1):1-9.
16. Orchard P, Manickam N, Ventresca C, et al. Human and rat skeletal muscle single-nuclei multiomic integrative analyses nominate causal cell types, regulatory elements, and SNPs for complex traits. *Genome Res*. 2021;31(12):2258-2276.
17. Saleh KK, Xi H, Switzler C, et al. Single cell sequencing maps skeletal muscle cellular diversity as disease severity increases in dystrophic mouse models. *iScience*. 2022;25(11):105415.
