# Supplementary material for: Generation of allogeneic and xenogeneic functional muscle stem cells for intramuscular transplantation
Source: J Clin Invest. 2024 May 7;134(12):e166998. doi: 10.1172/JCI166998 (PMC11178549; doi:10.1172/JCI166998)

Full unedited gel for Figure 1K

Full unedited gel

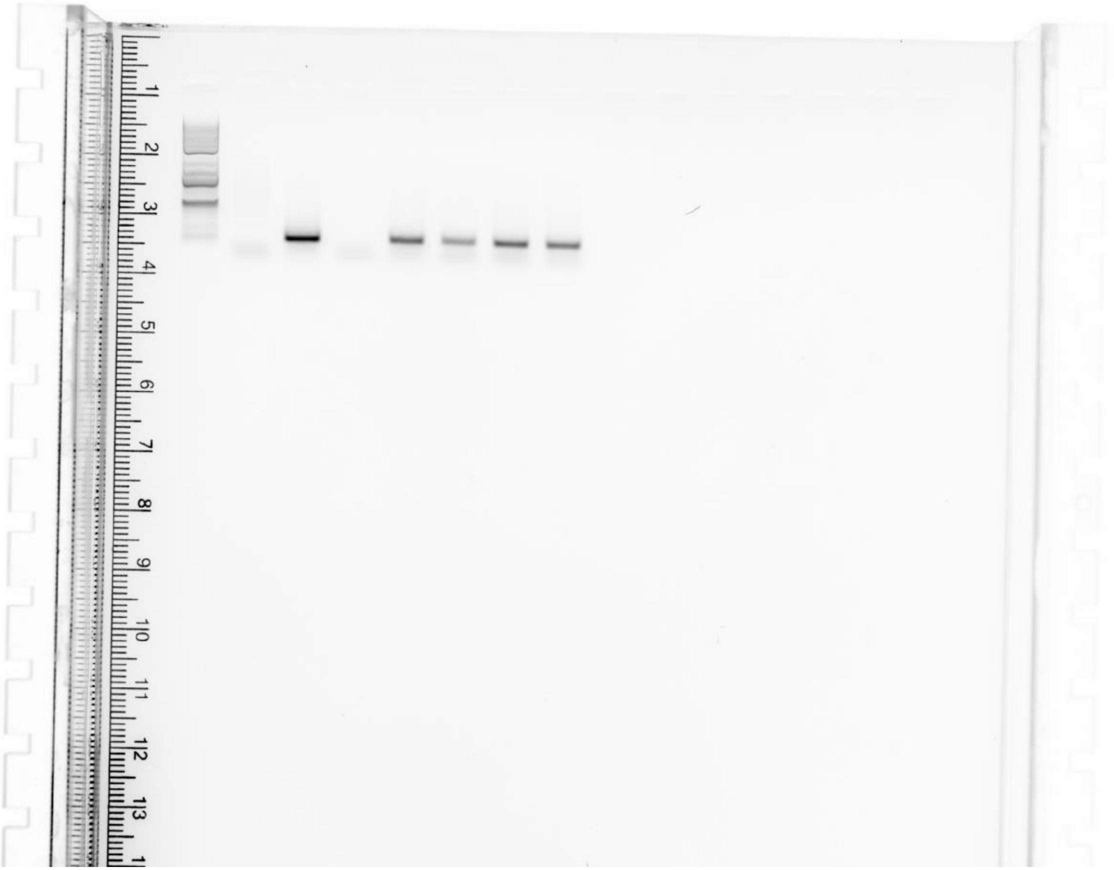

Full unedited gel with marked bands used in the figure

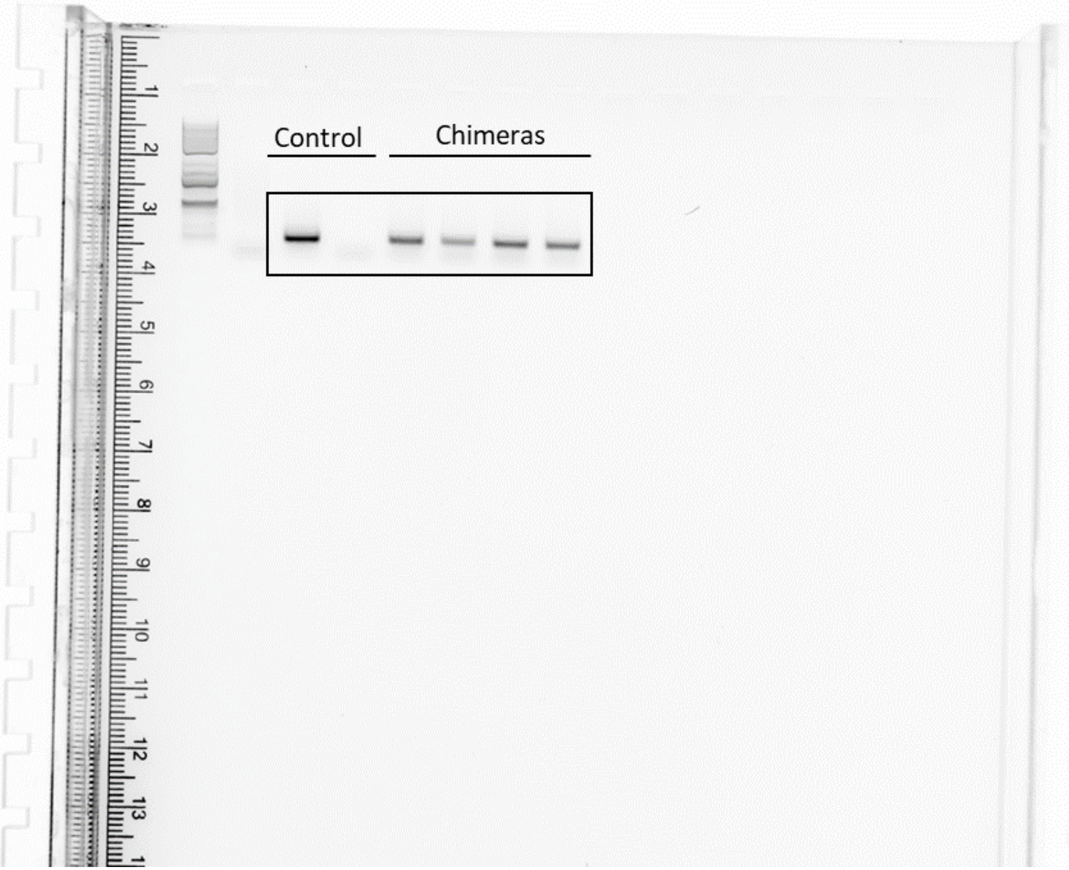

Full unedited gel for Figure 2C

Full unedited gel

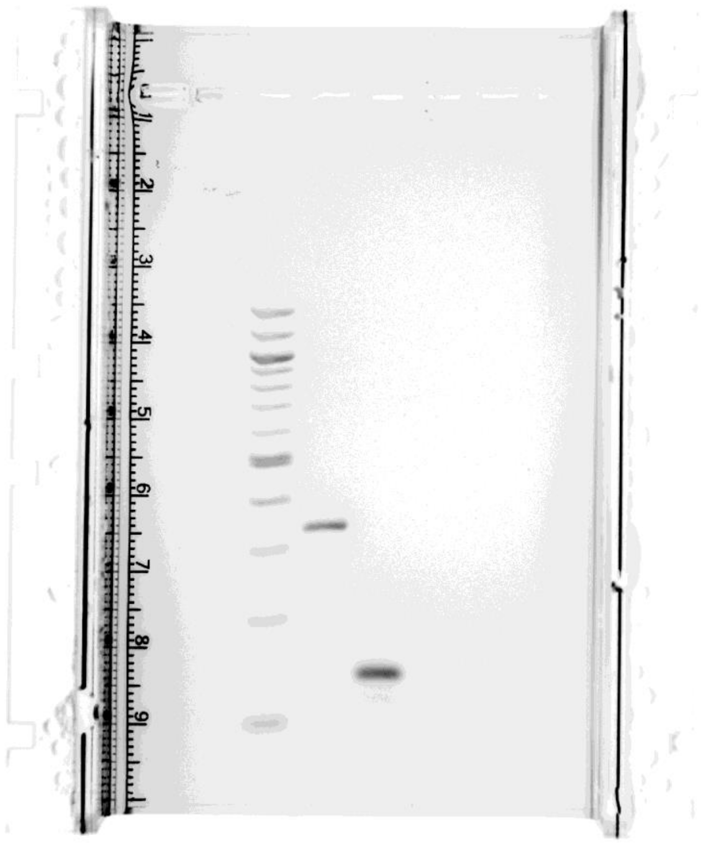

Full unedited gel with marked bands used in the figure

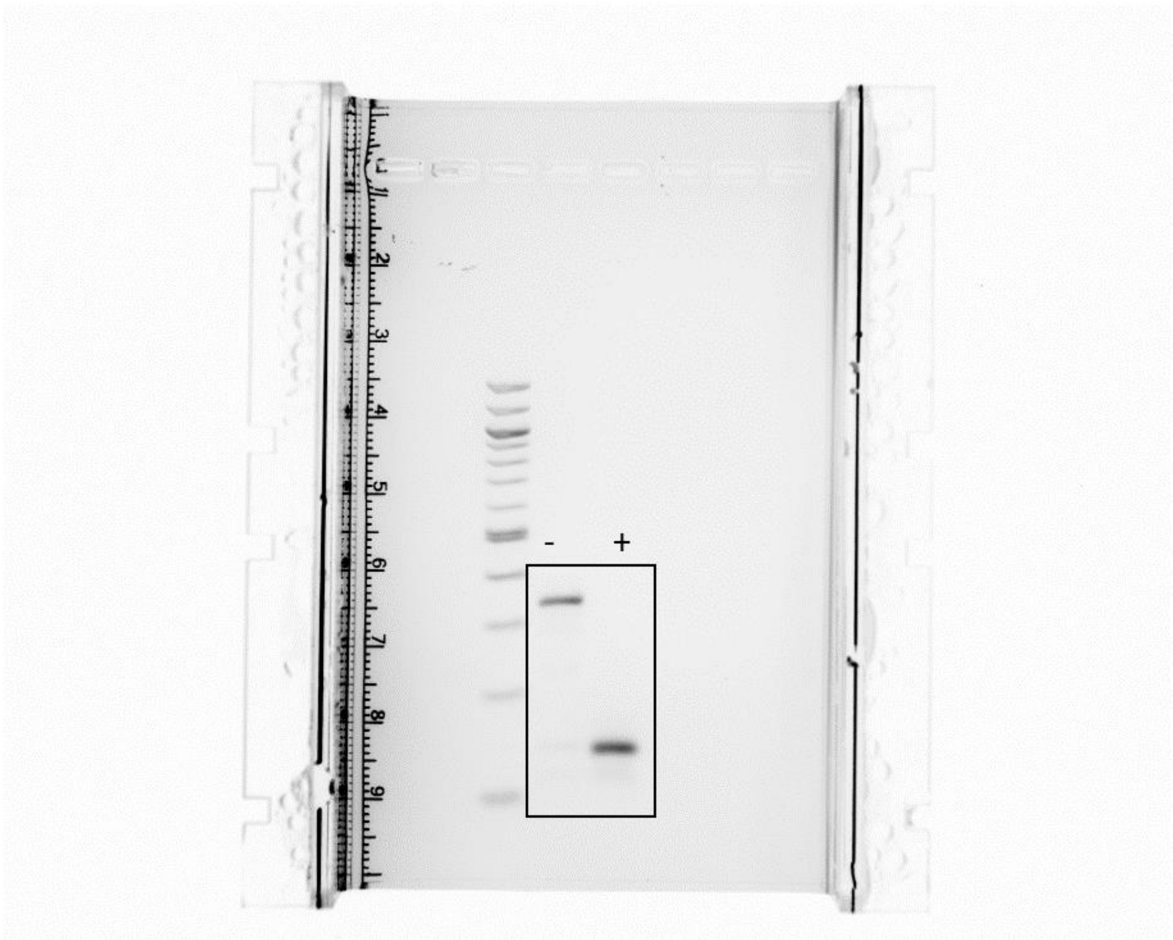

Full unedited gel for Figure 2F

Full unedited gel

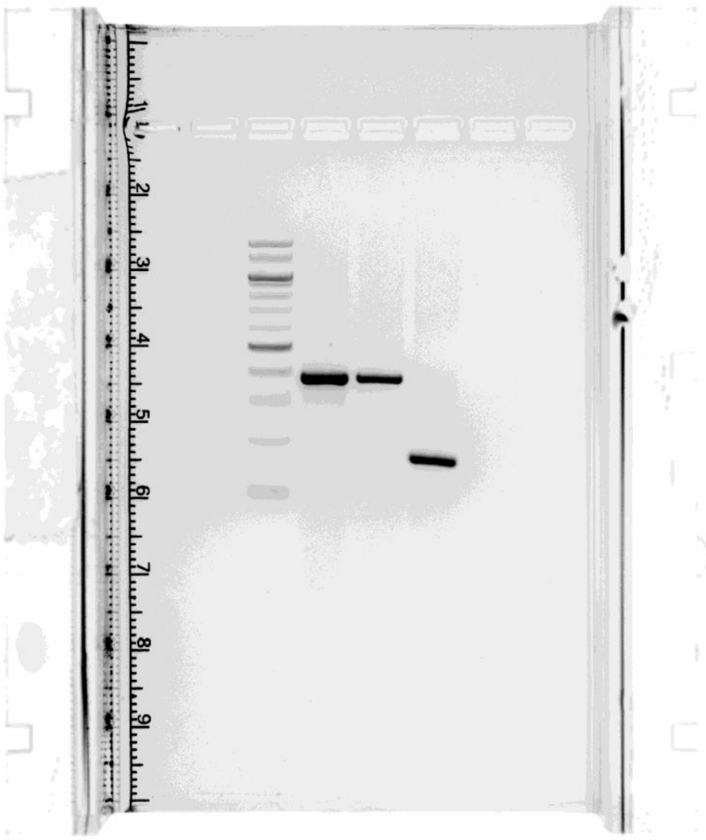

Full unedited gel with marked bands used in the figure

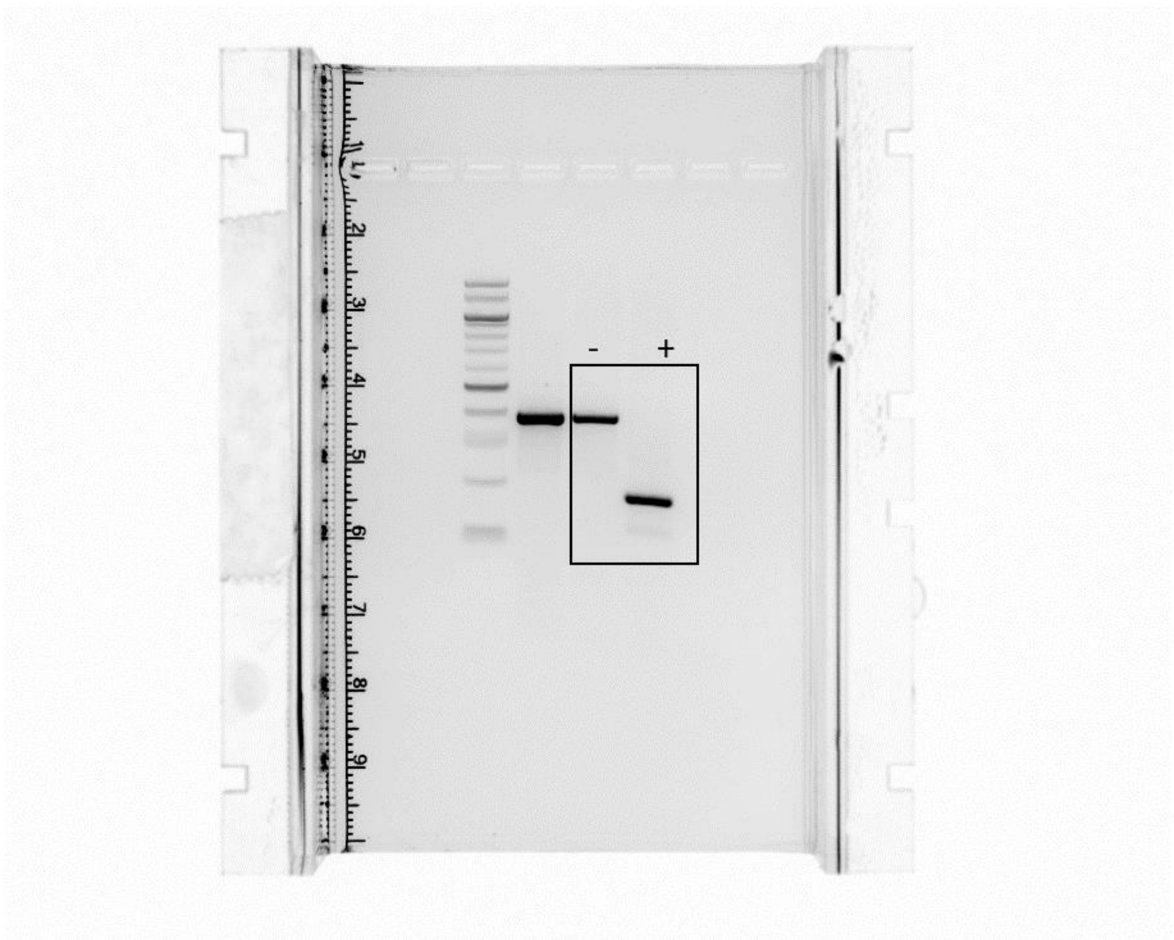

Full unedited gel for Figure 2H

Full unedited gel

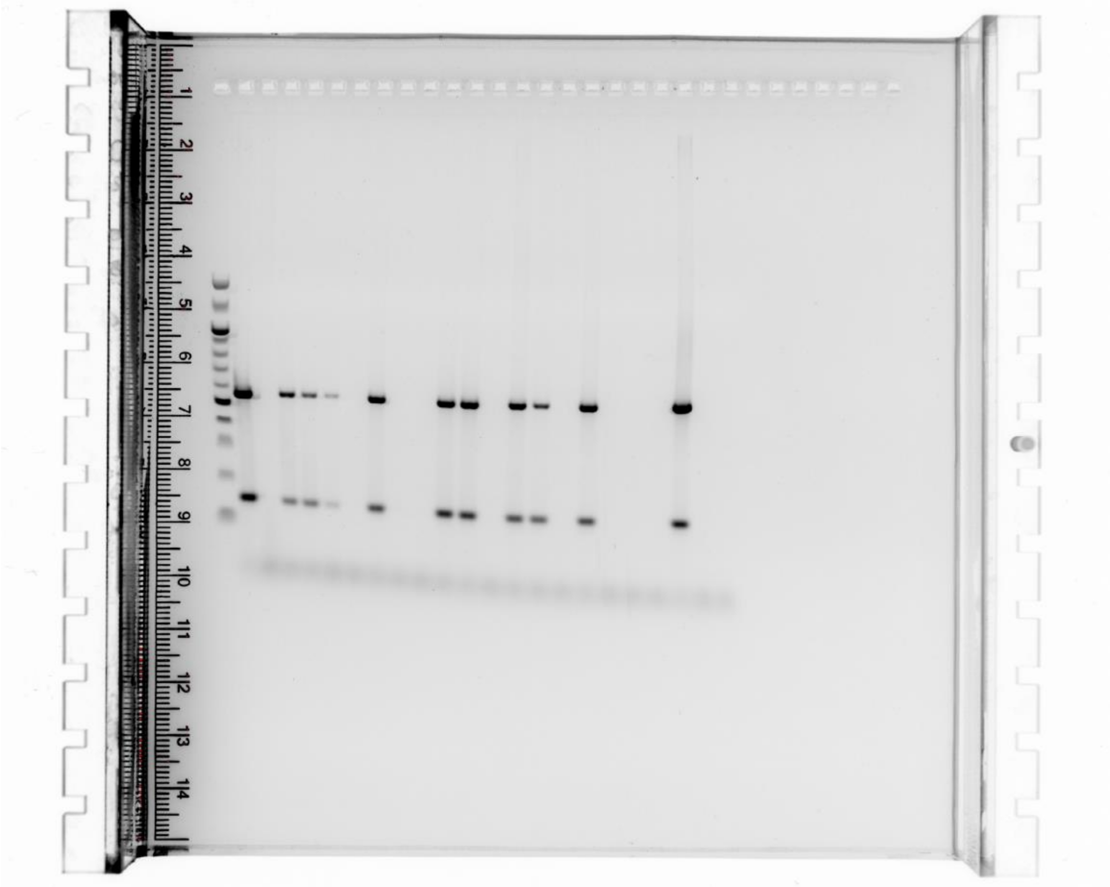

Full unedited gel with marked bands used in the figure

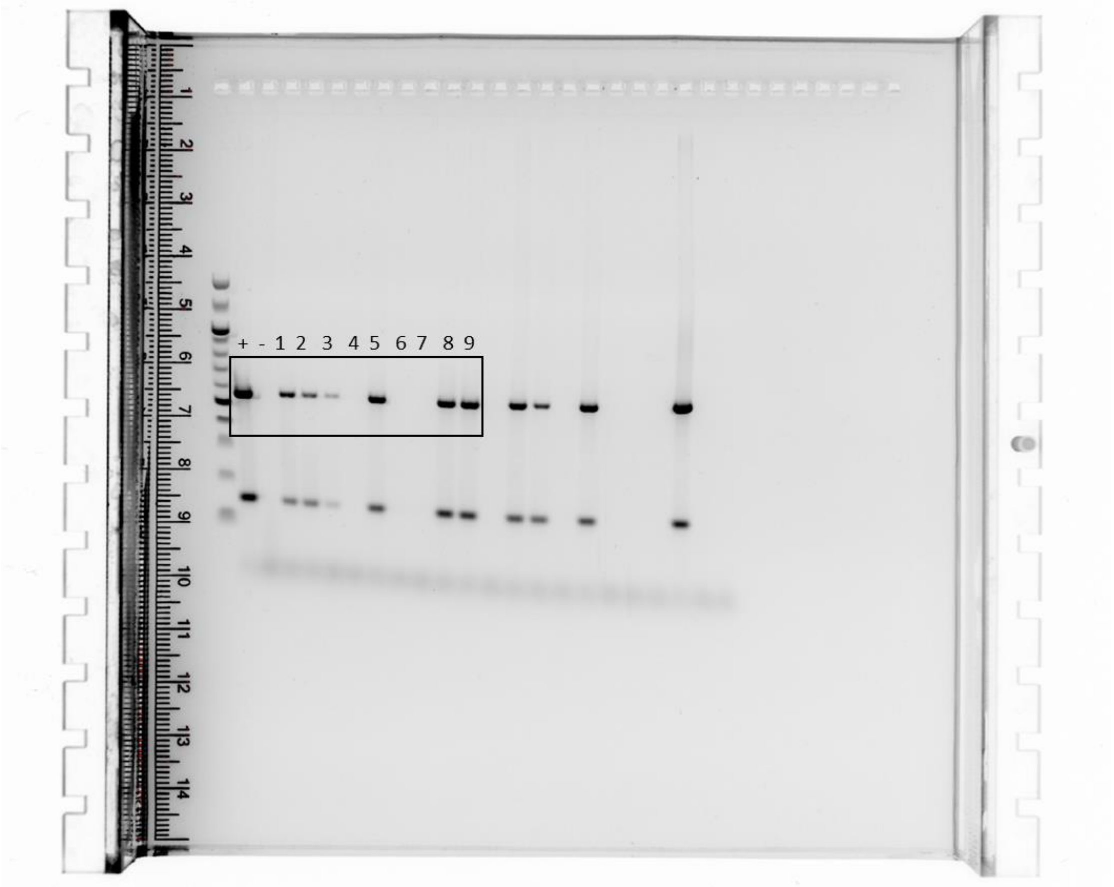

Full unedited gel for Figure 2N

Full unedited gel

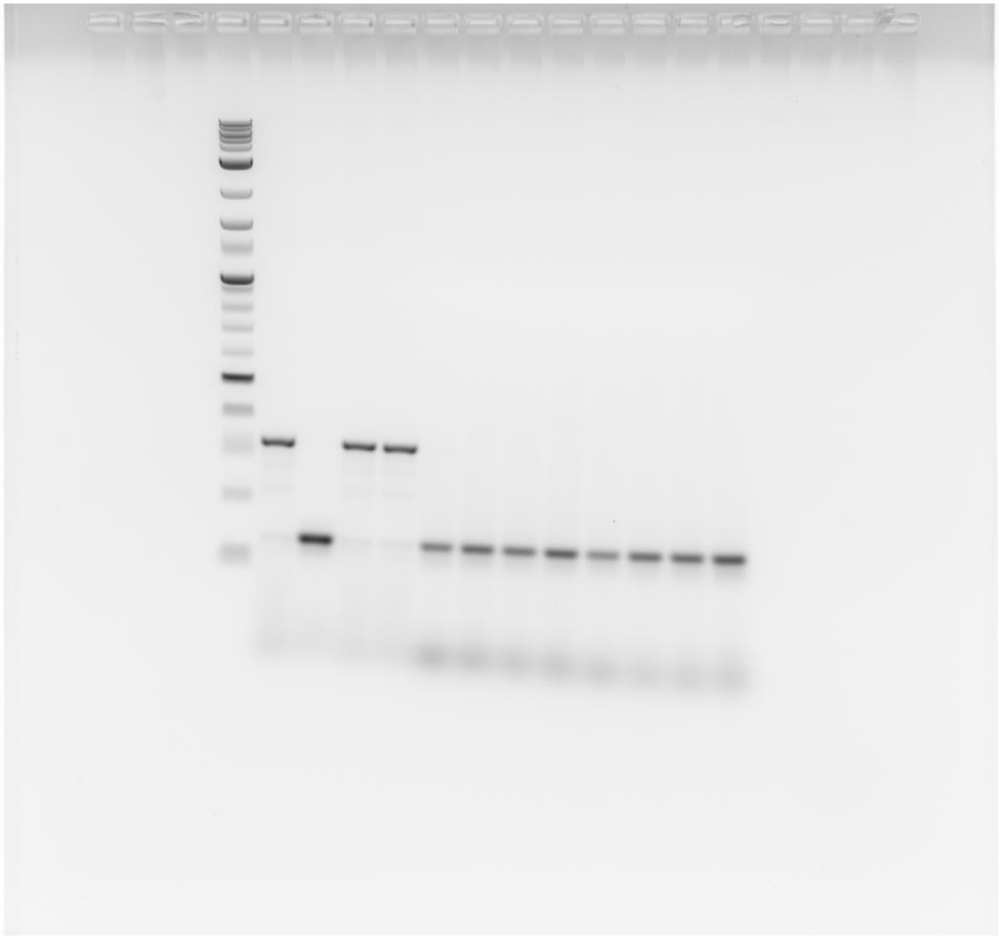

Full unedited gel with marked bands used in the figure

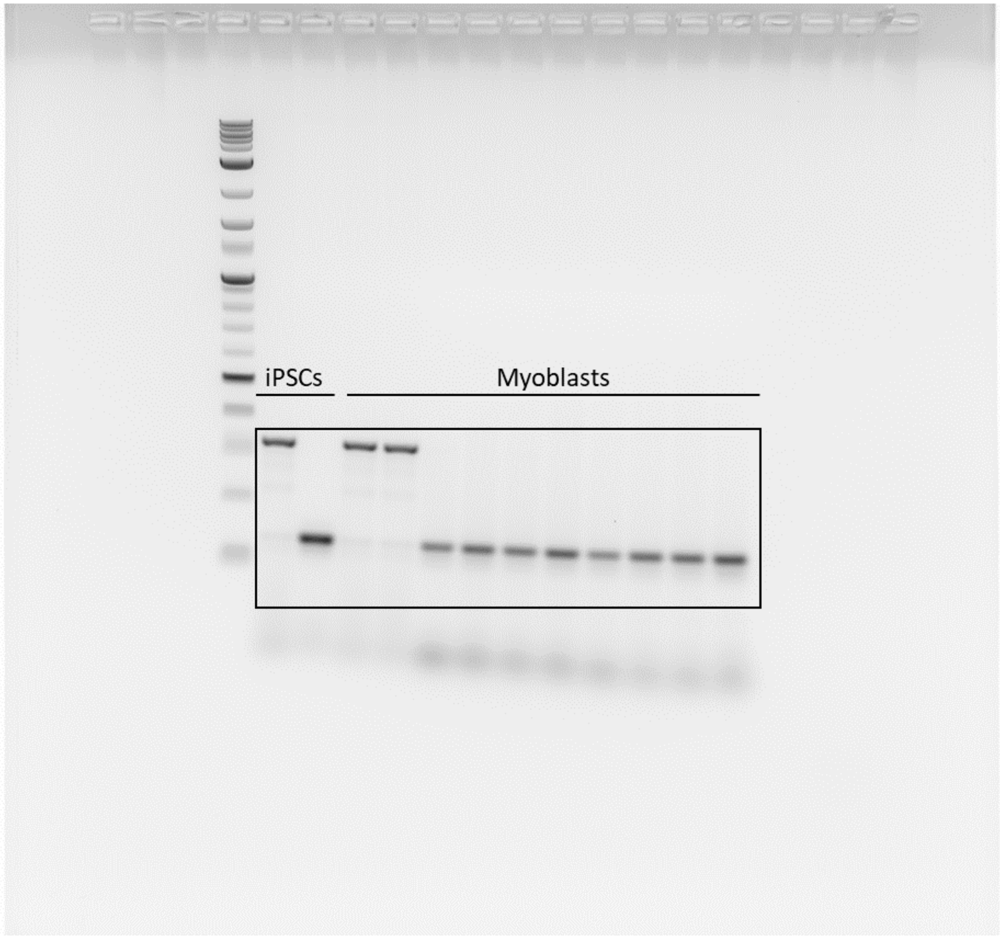

Full unedited gel for Figure 3F

Full unedited gel

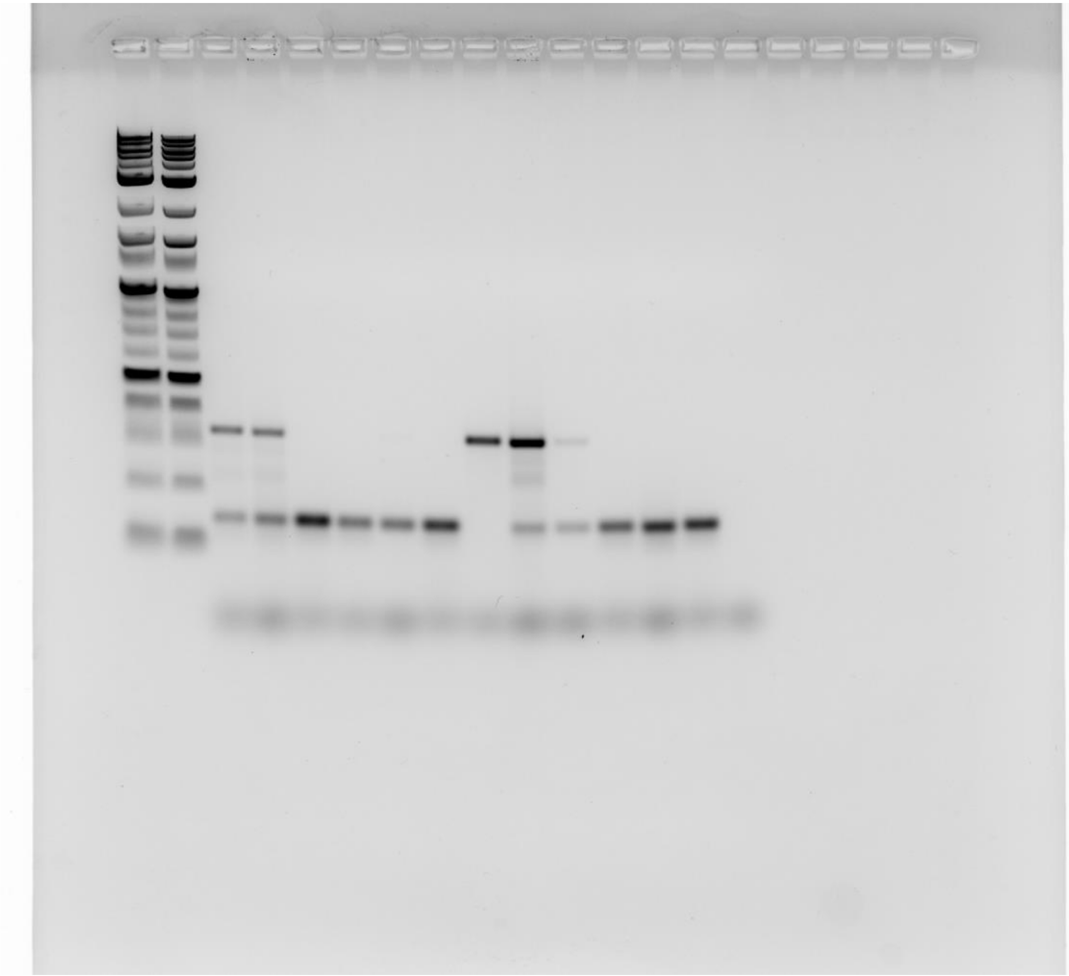

Full unedited gel with marked bands used in the figure

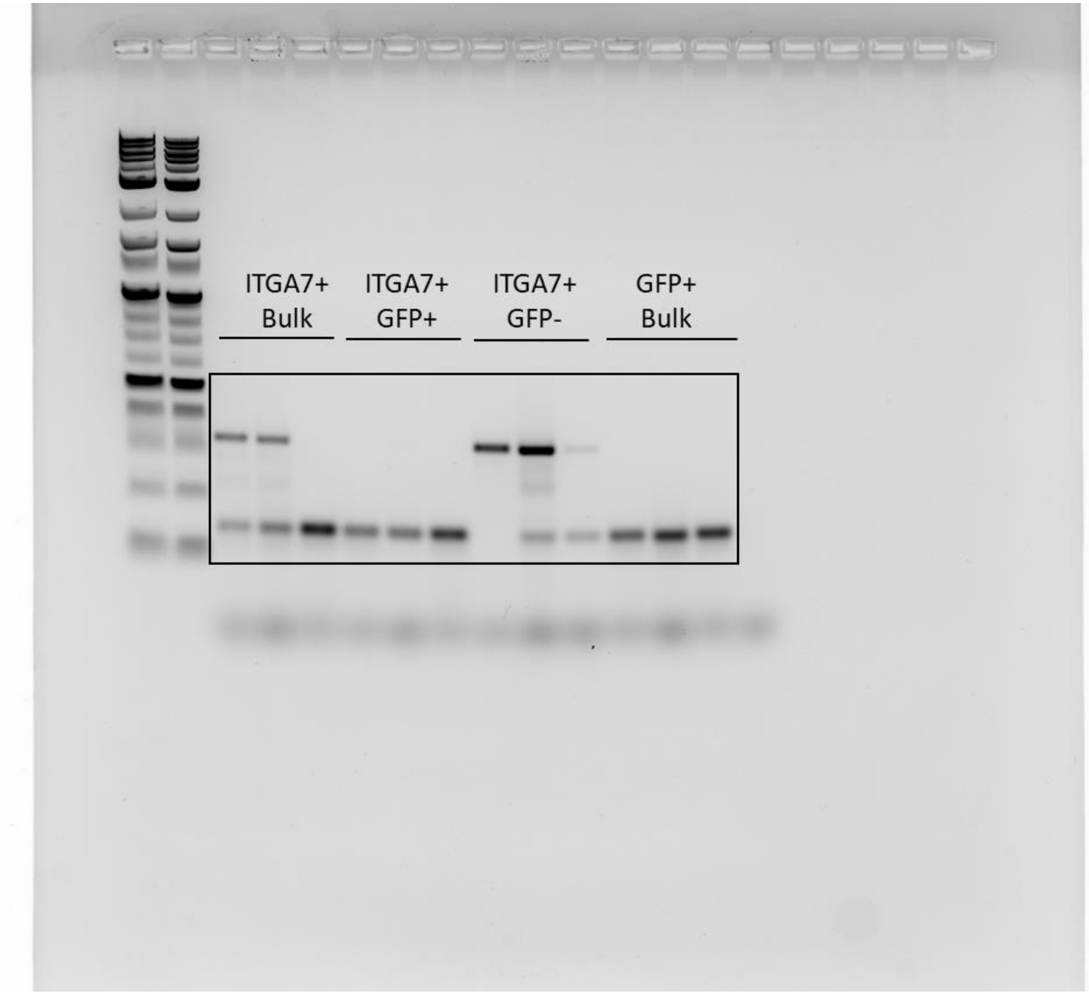

Full unedited gel for Figures 3H and 3K

Full unedited gel

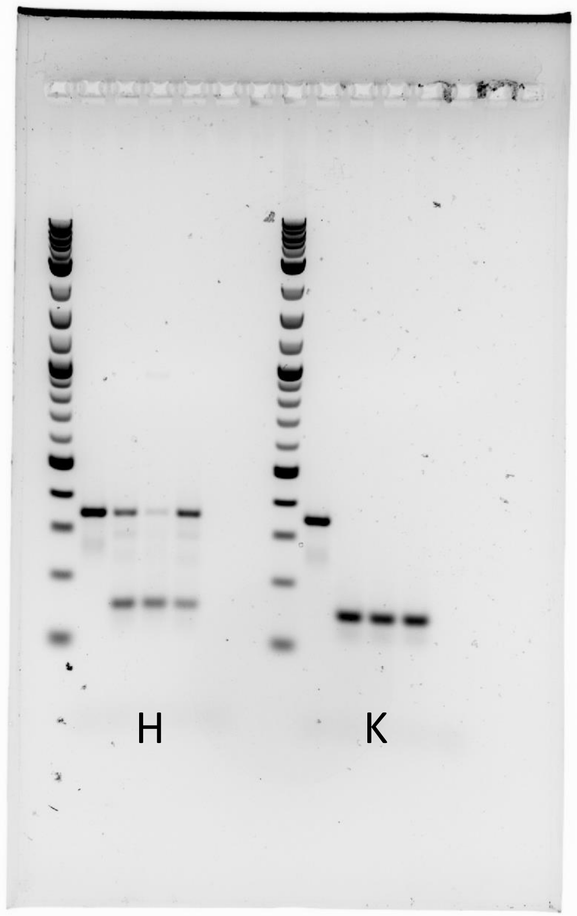

Full unedited gel with marked bands used in the figure

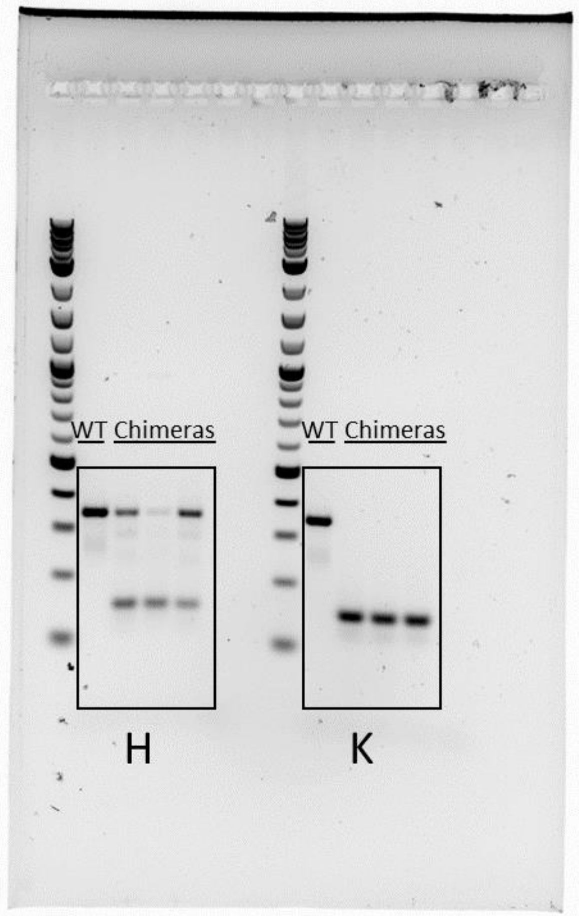

Full unedited gel for Figure 5D

Full unedited gel

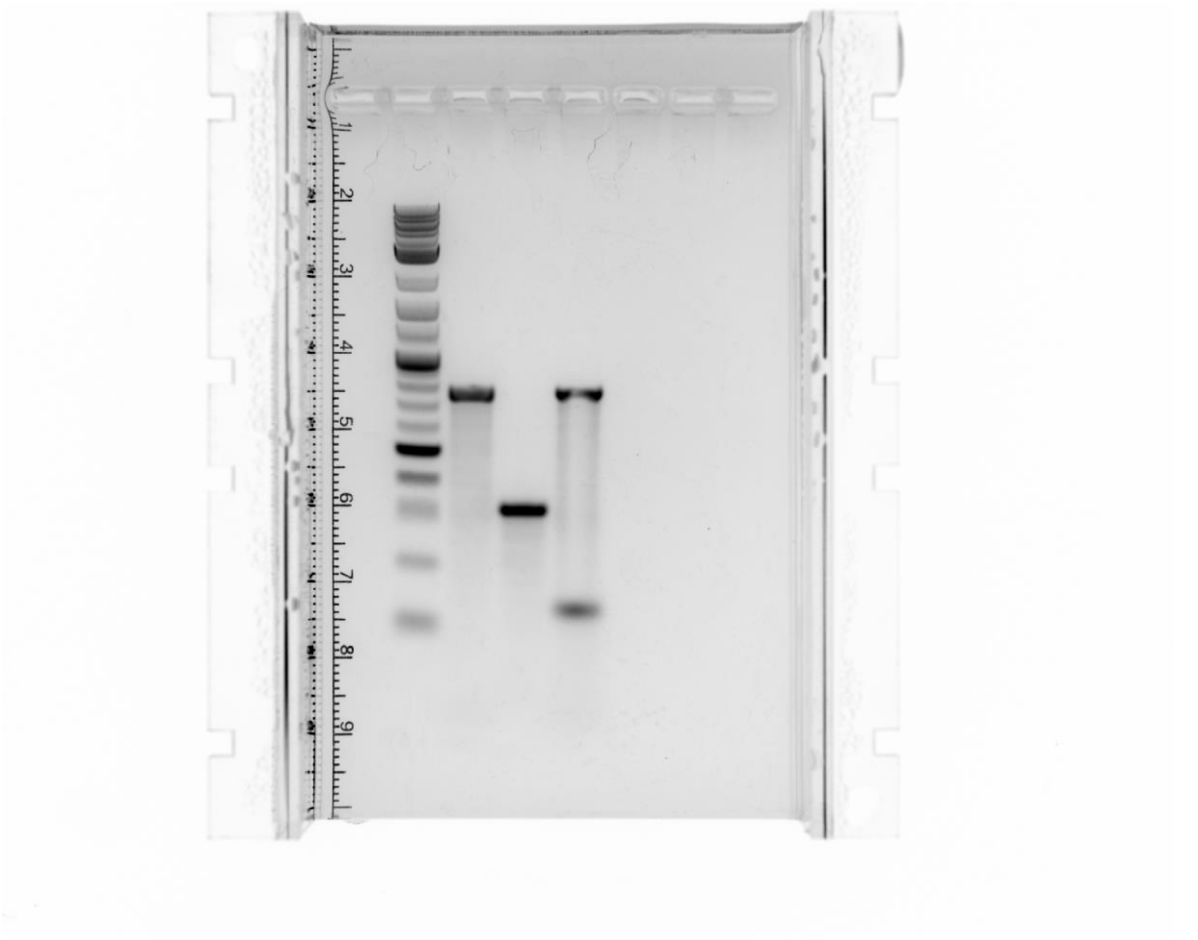

Full unedited gel with marked bands used in the figure

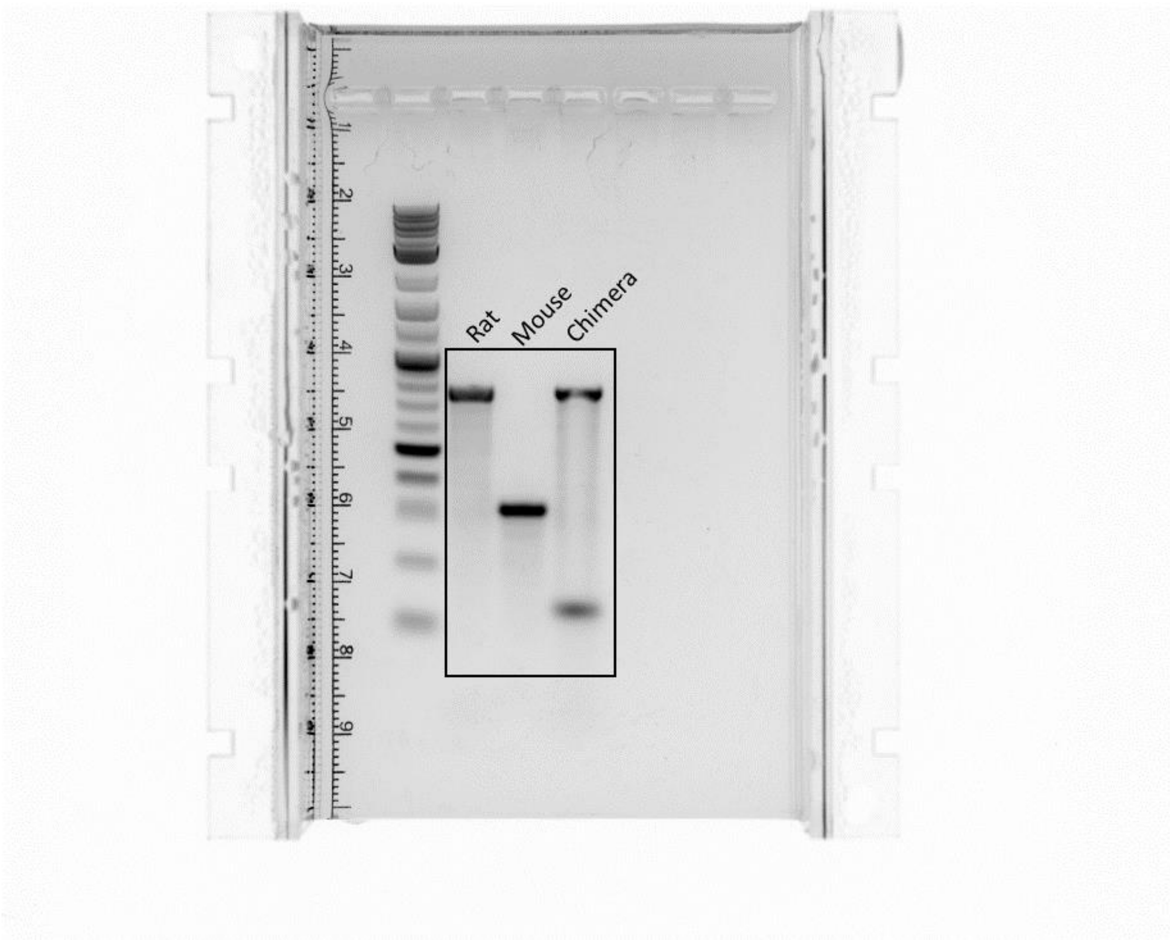

Full unedited gel for Figure 6D

Full unedited gel

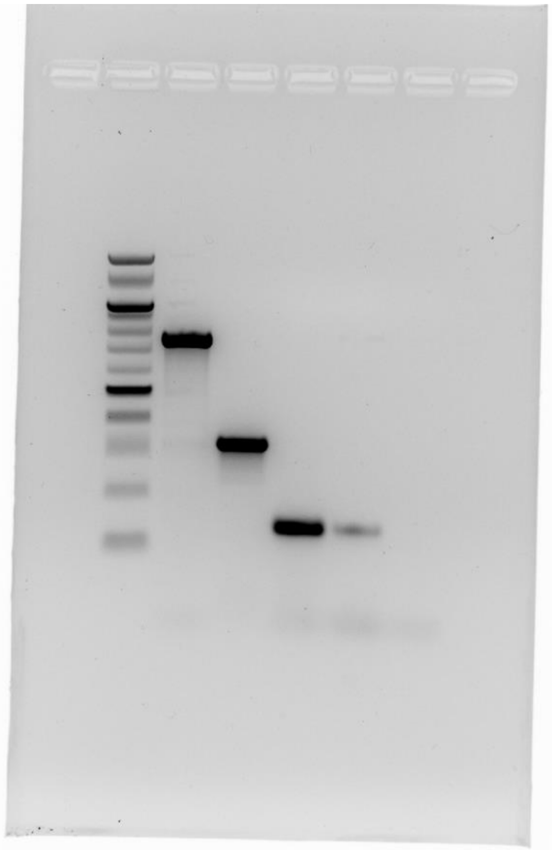

Full unedited gel with marked bands used in the figure

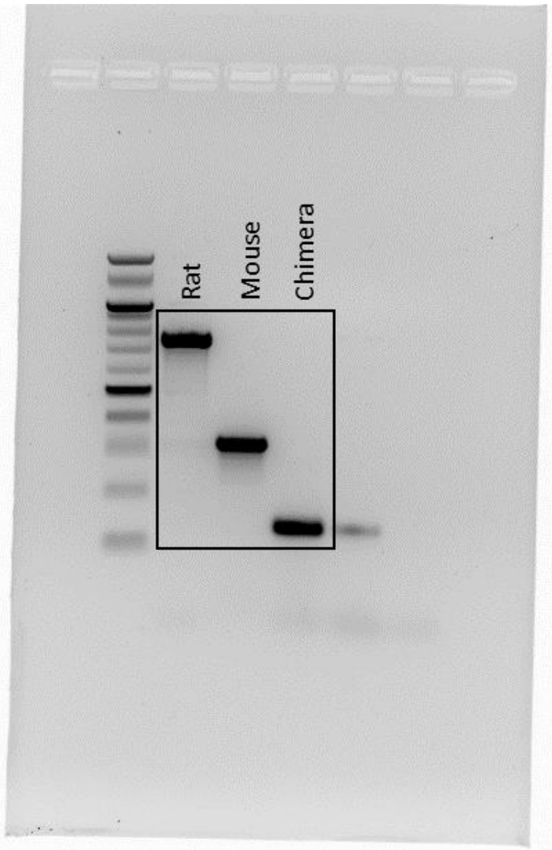

Full unedited gel for Figure 6E

Full unedited gel

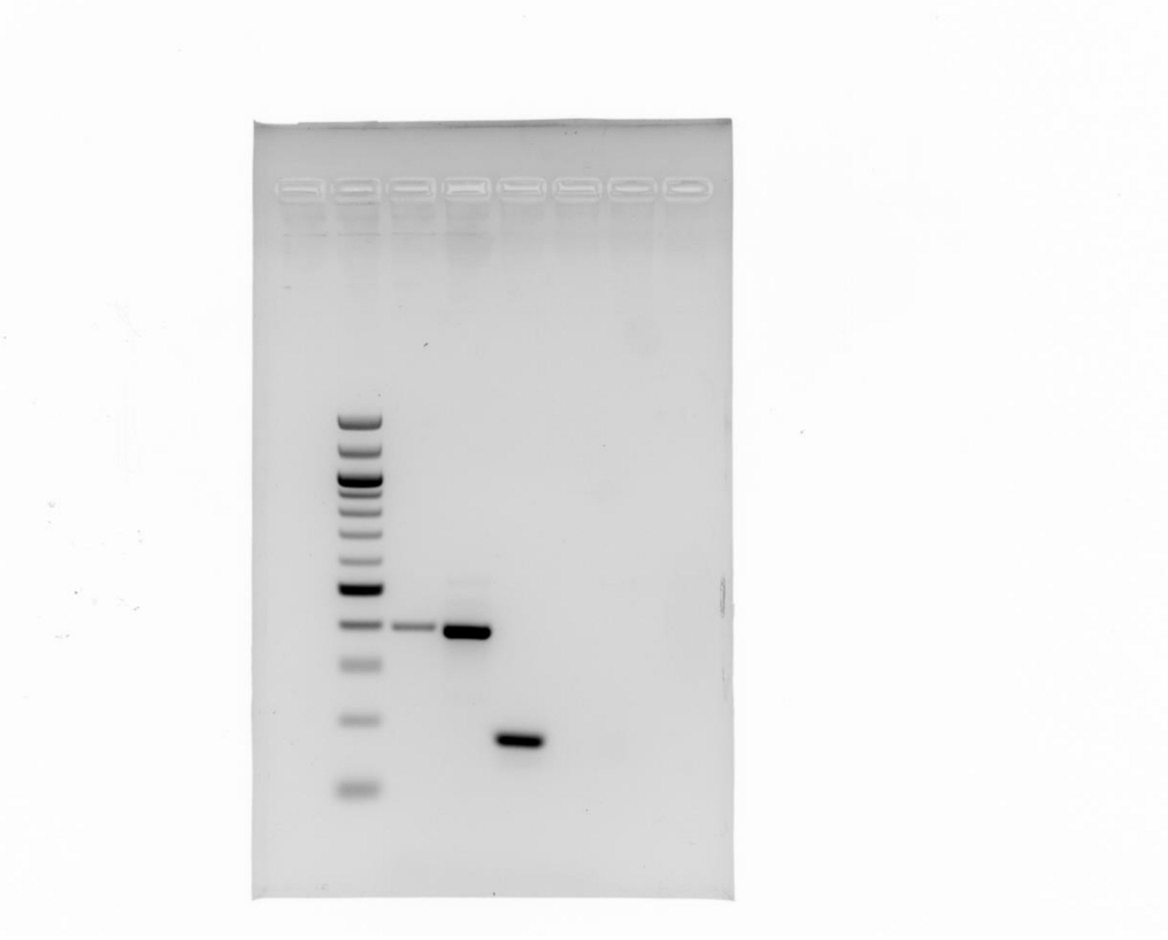

Full unedited gel with marked bands used in the figure

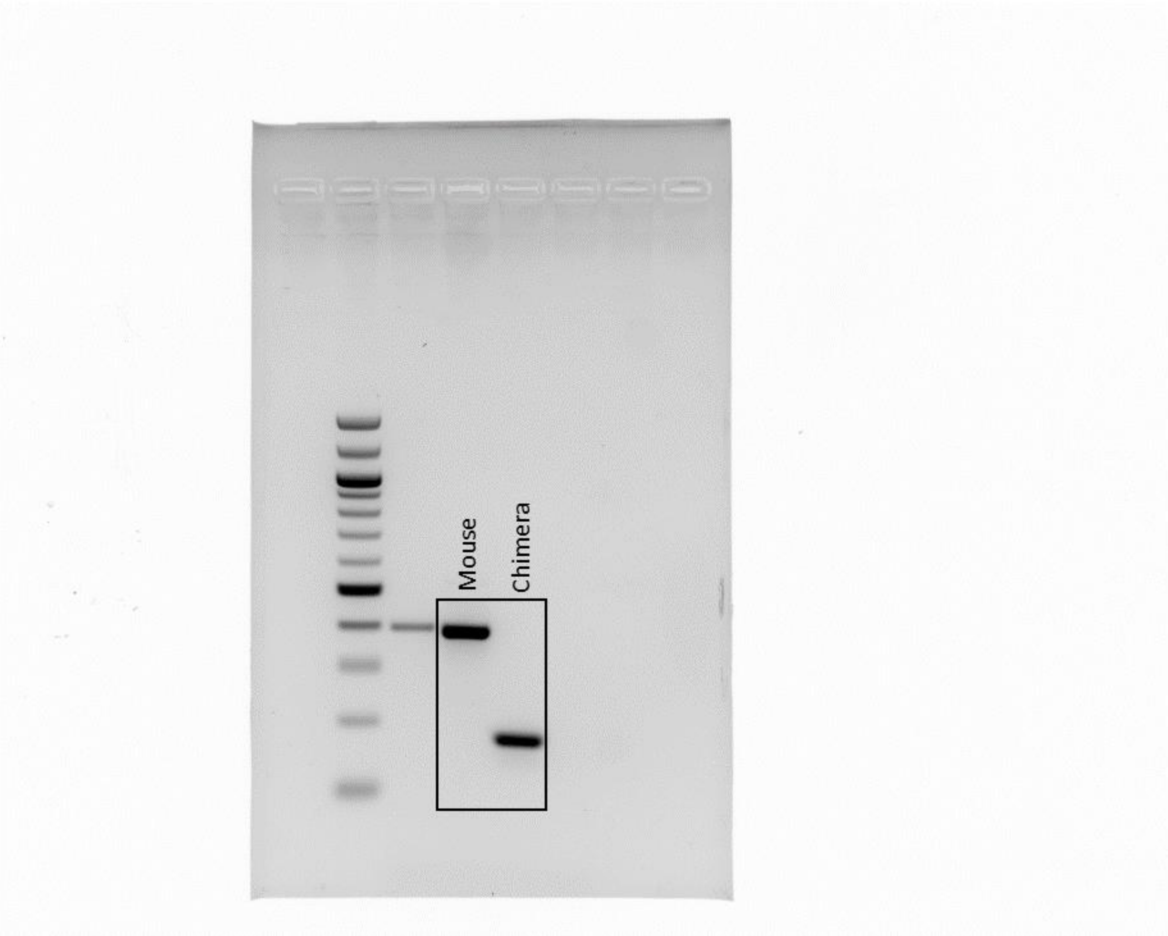

Full unedited gel for Figure 7I

Full unedited gel

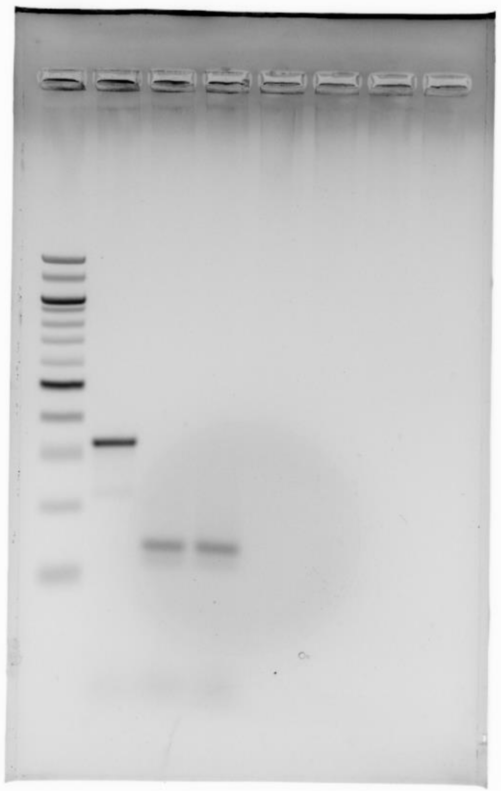

Full unedited gel with marked bands used in the figure

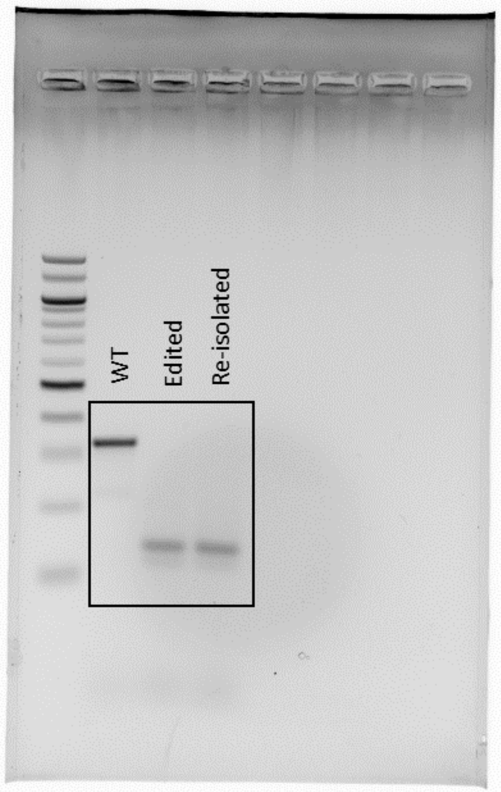

Full unedited gel for Supplemental Figure 1A

Full unedited gel

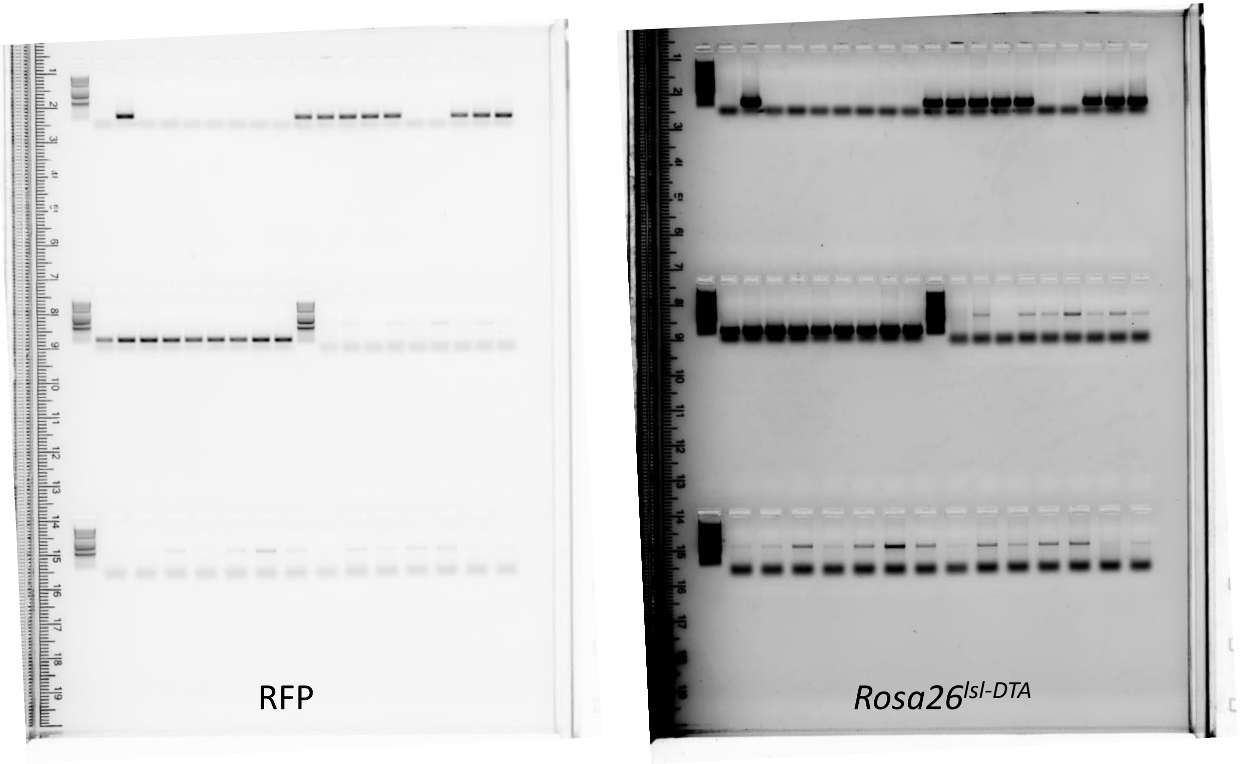

Full unedited gel with marked bands used in the figure

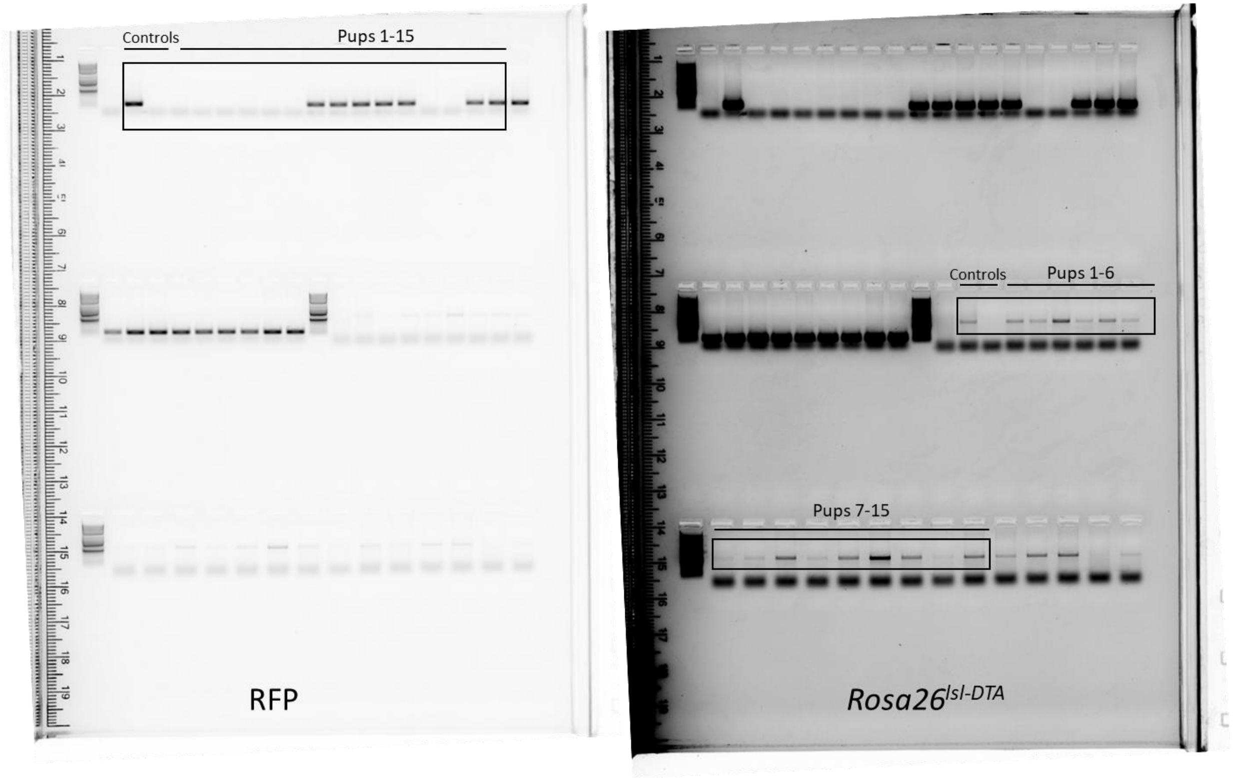

Full unedited gel for Supplemental Figure 2I

Full unedited gel

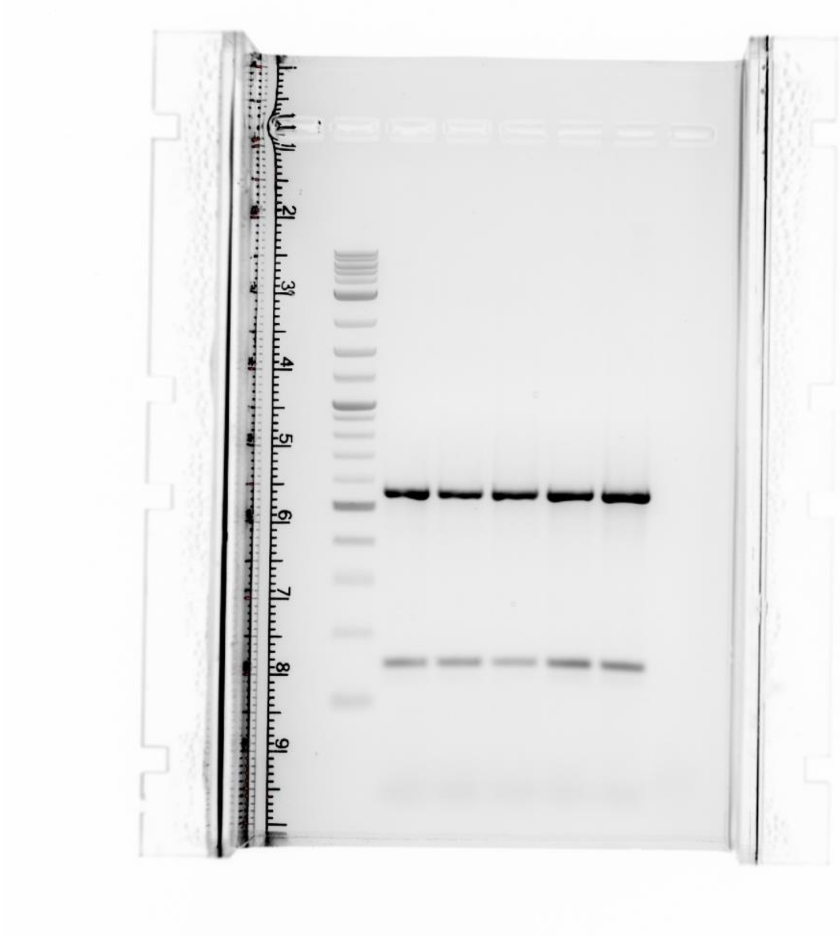

Full unedited gel with marked bands used in the figure

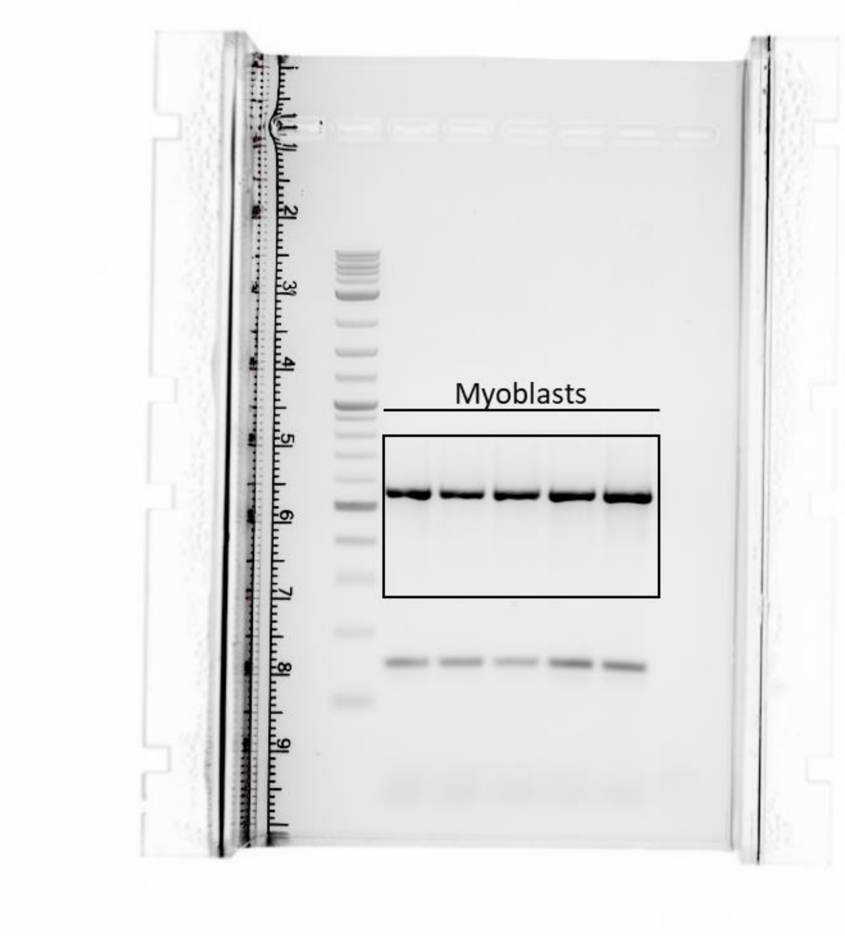

Full unedited gel for Supplemental Figure 2J

Full unedited gel

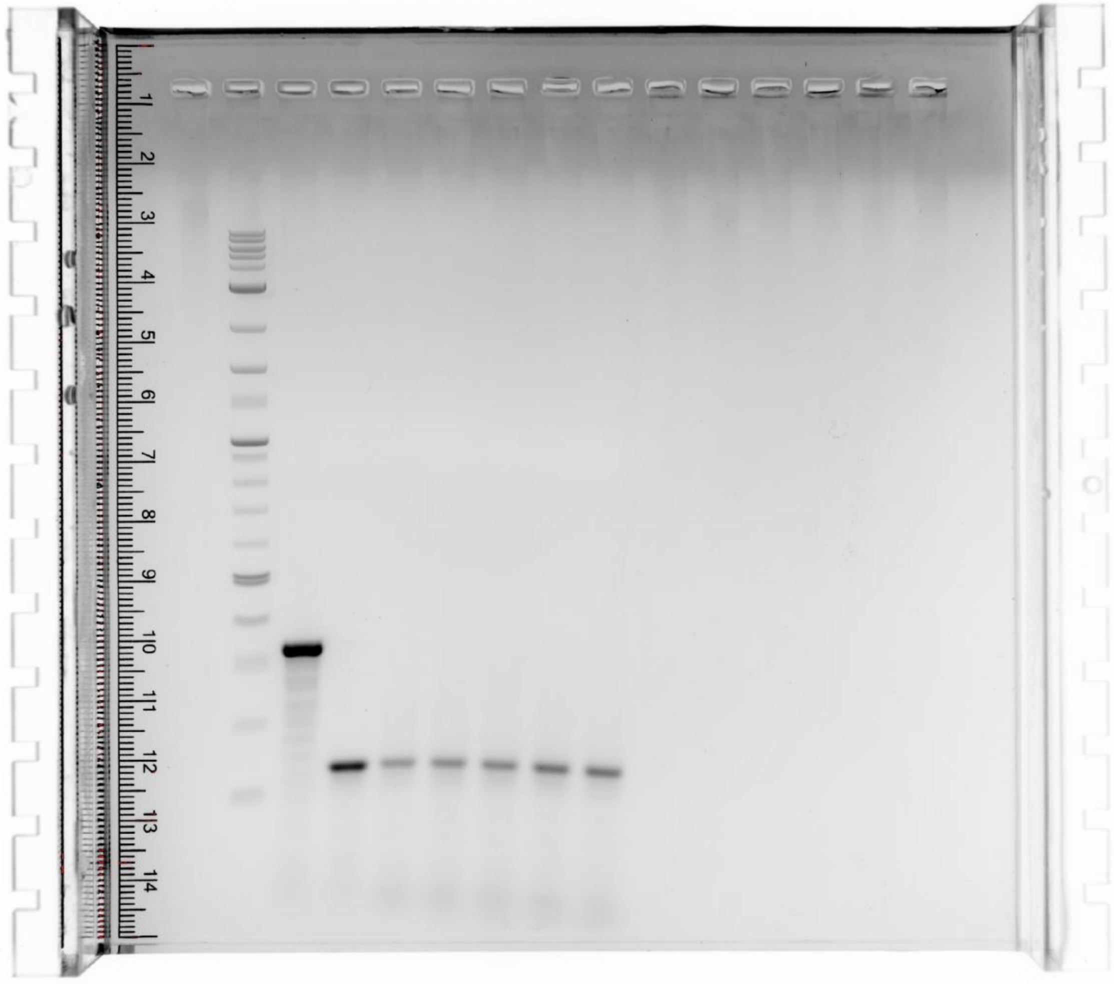

Full unedited gel with marked bands used in the figure

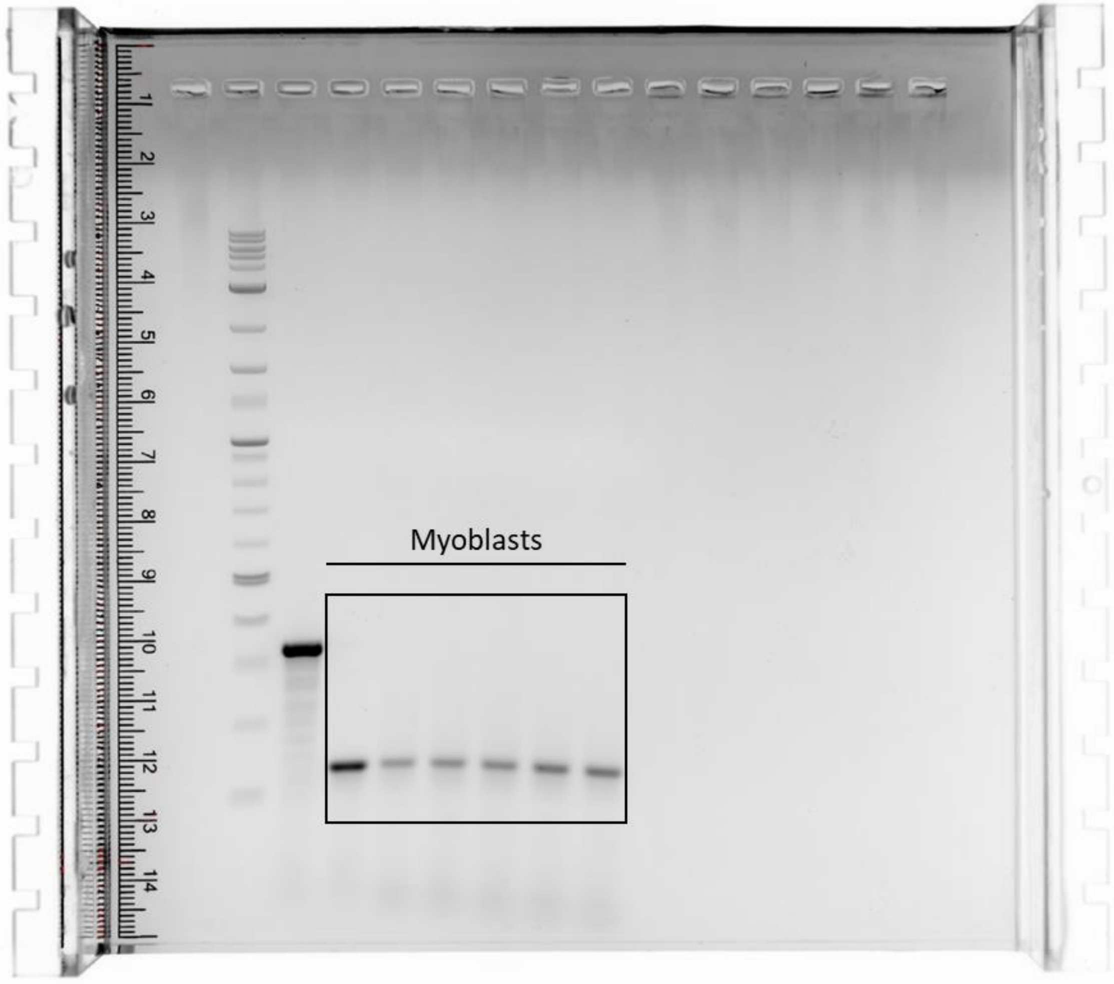

Full unedited gel for Supplemental Figure 3G

Full unedited gel

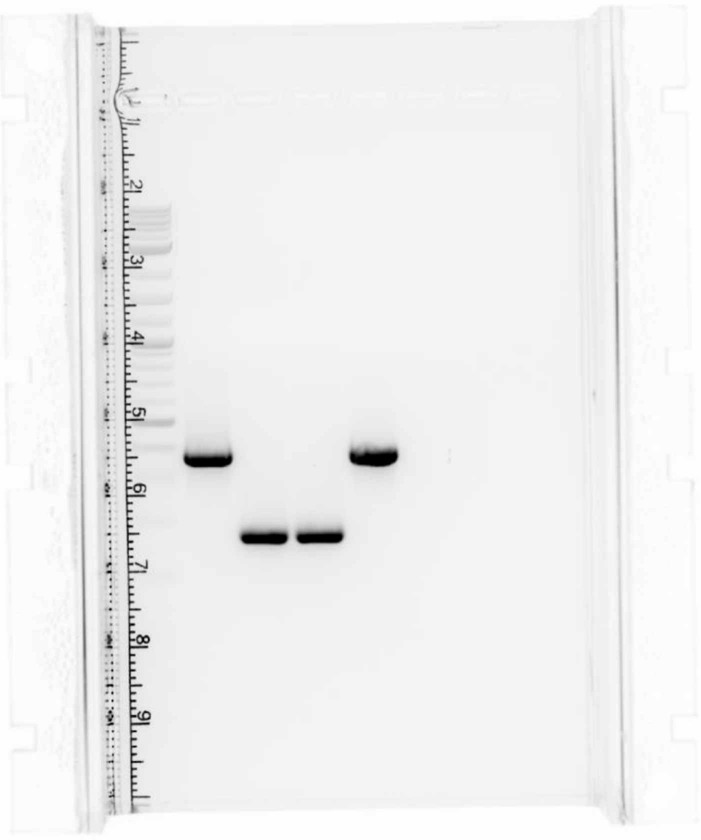

Full unedited gel with marked bands used in the figure

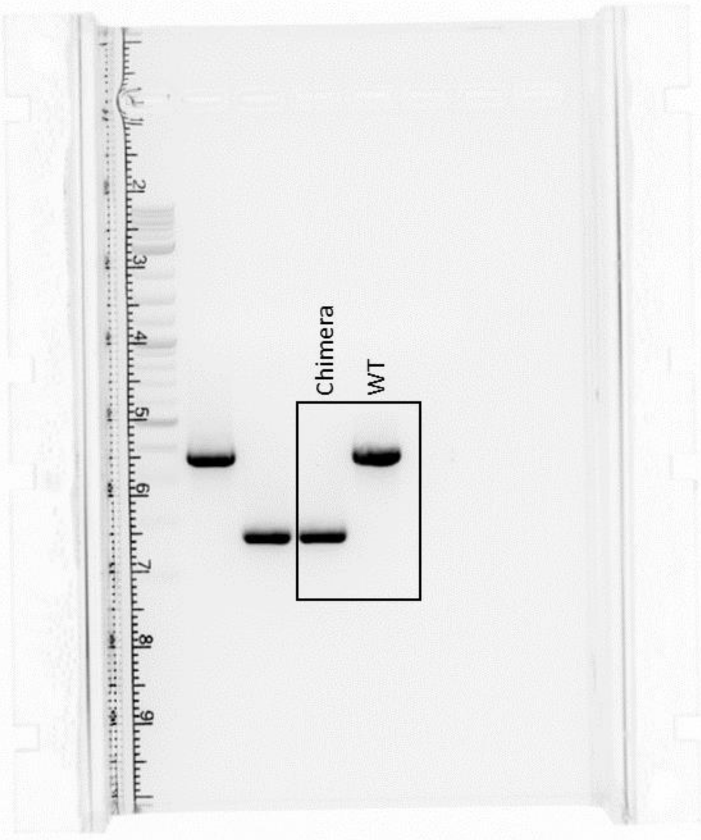

Full unedited gel for Supplemental Figure 4A

Full unedited gel

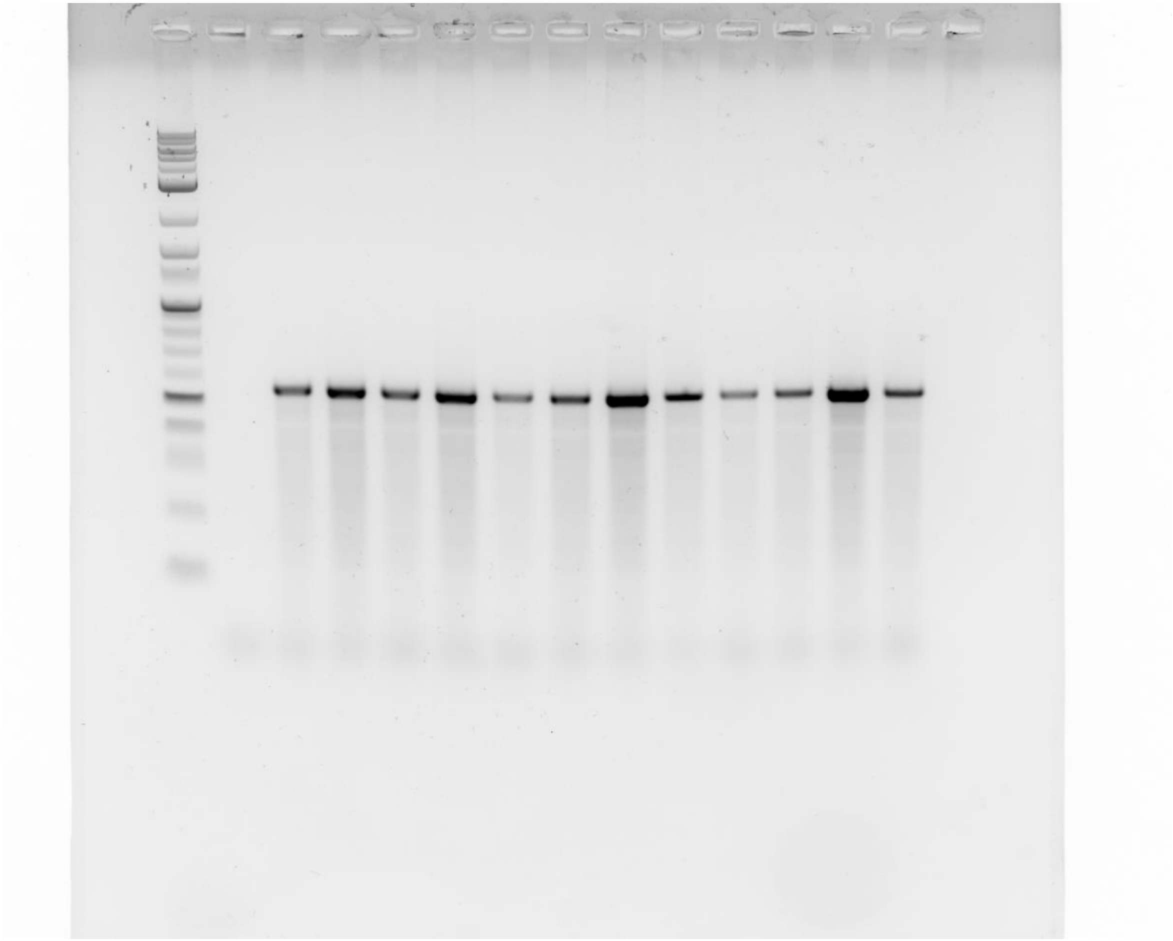

Full unedited gel with marked bands used in the figure

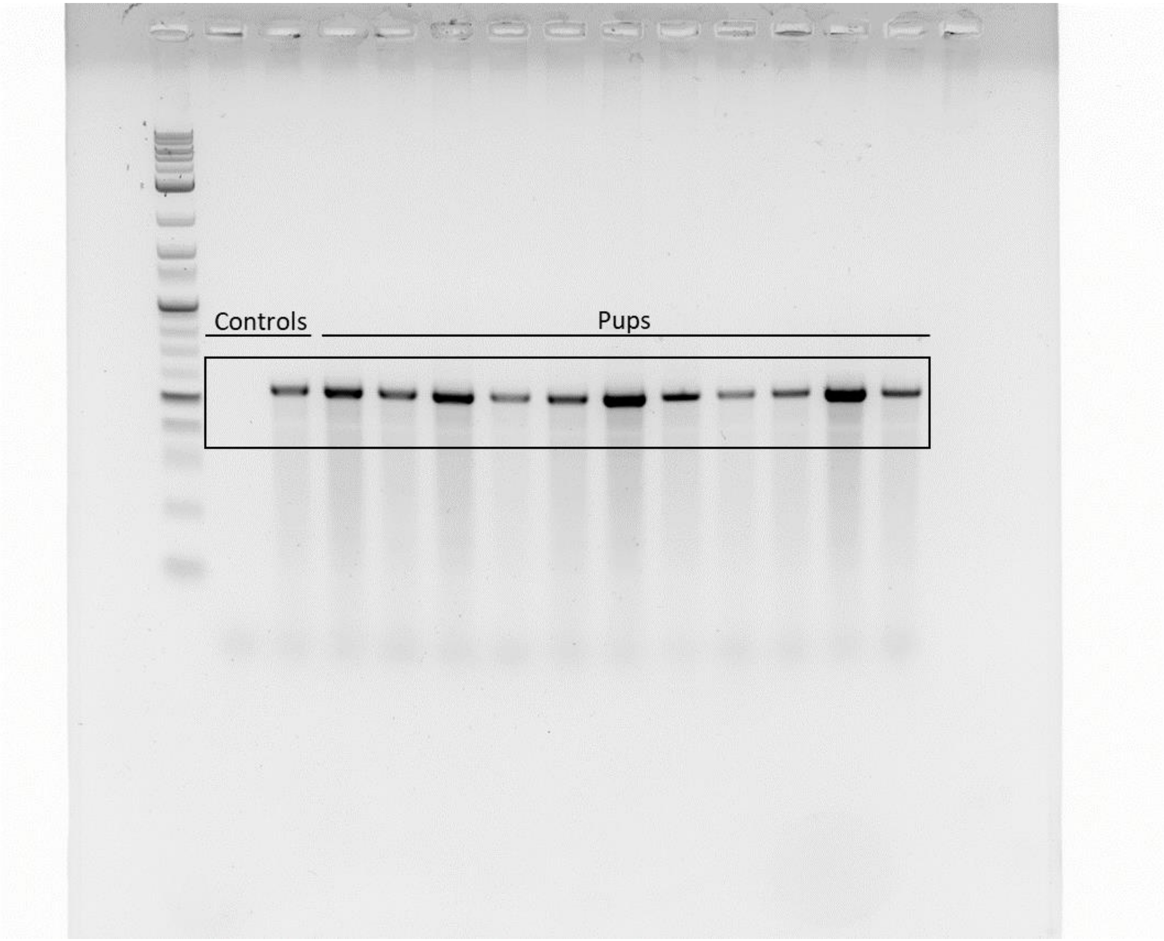

Full unedited gel for Supplemental Figure 7B

Full unedited gel

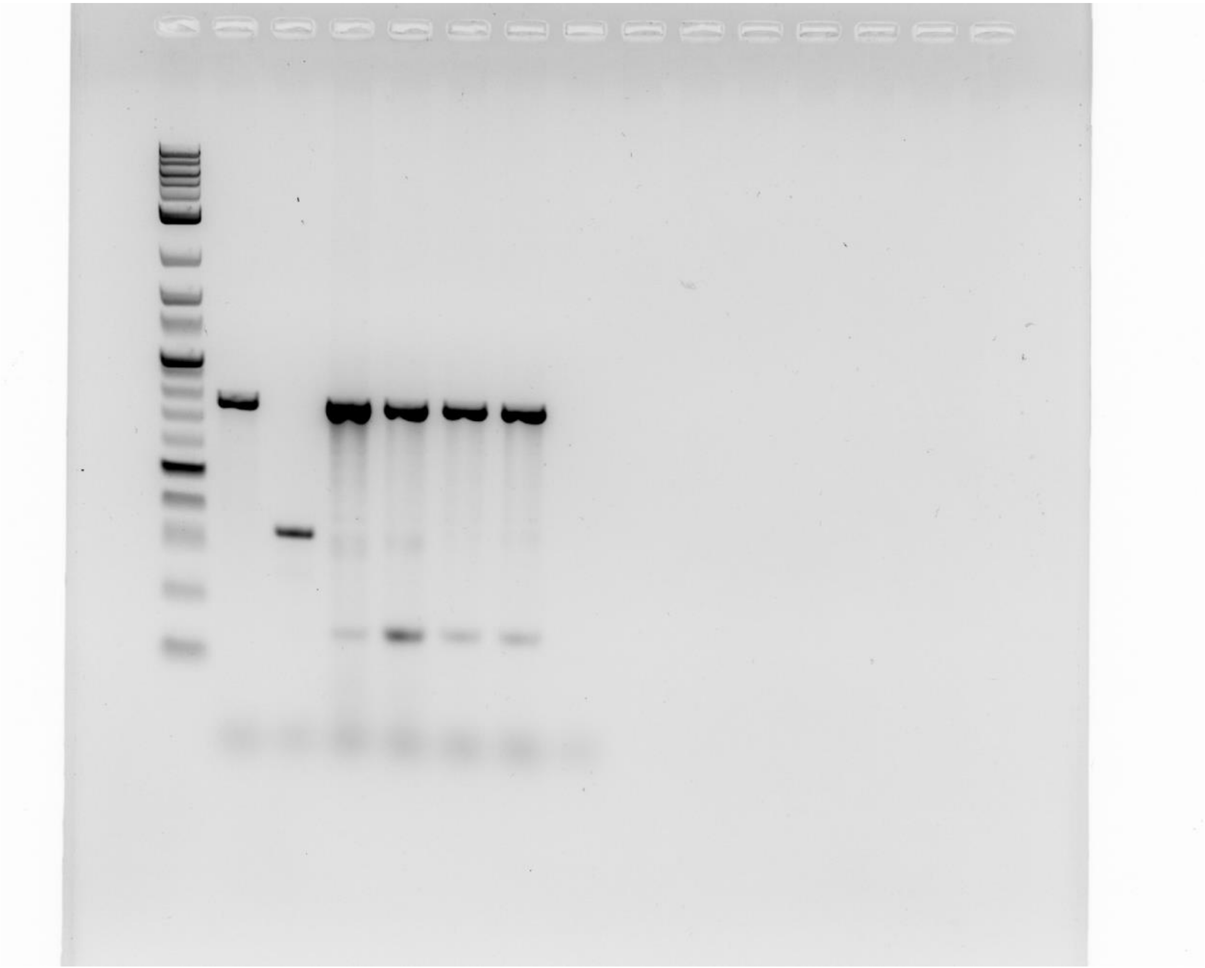

Full unedited gel with marked bands used in the figure

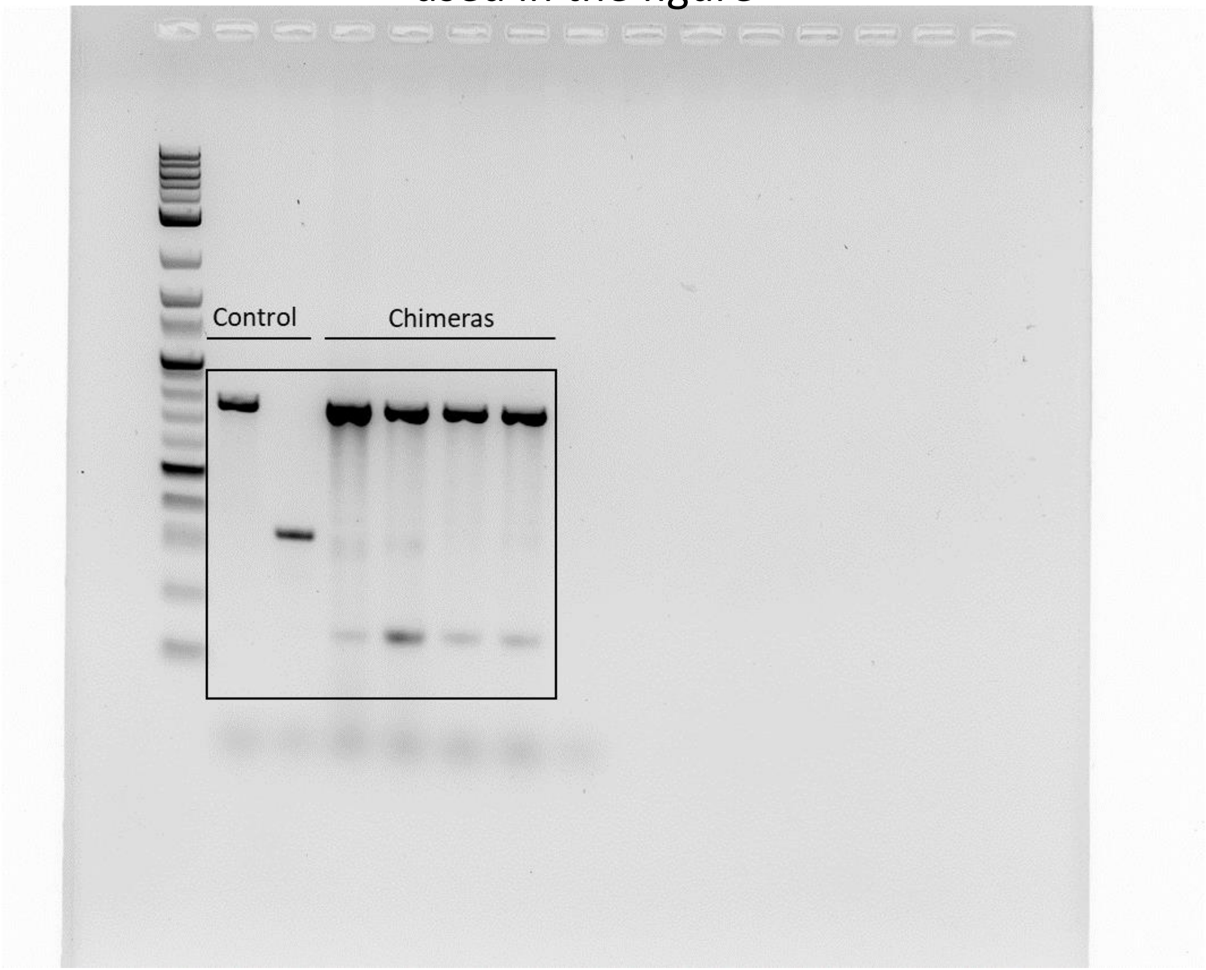

Full unedited gel for Supplemental Figure 7C

Full unedited gel

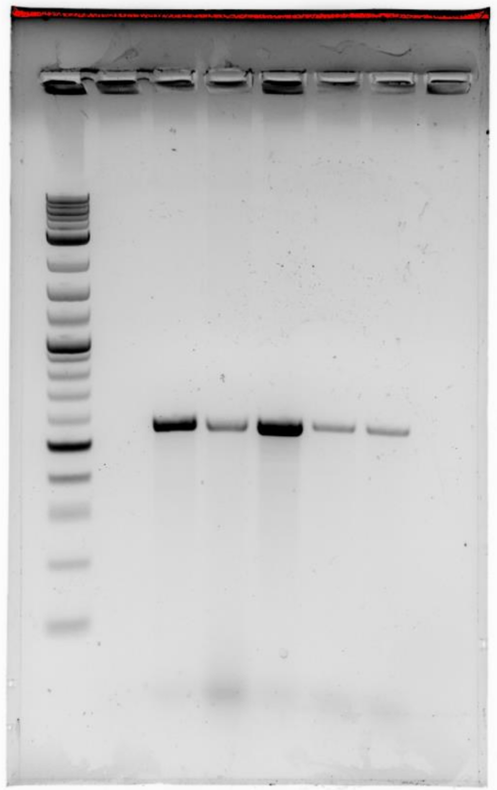

Full unedited gel with marked bands used in the figure

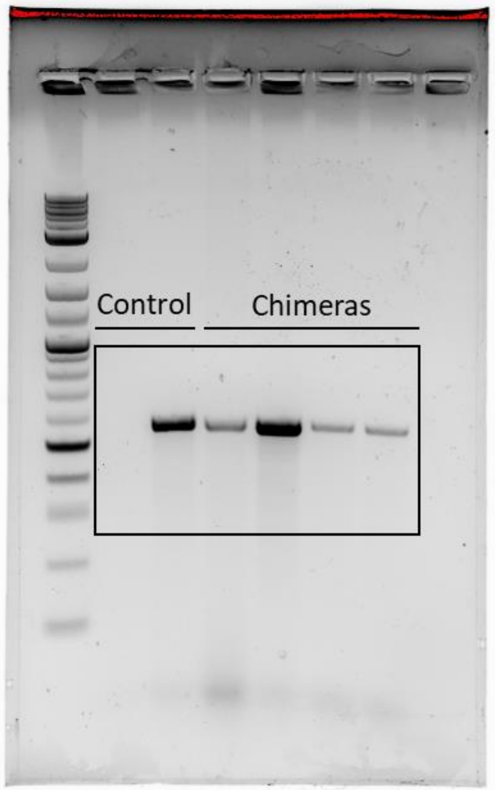

Full unedited gel for Supplemental Figure 7E

Full unedited gel

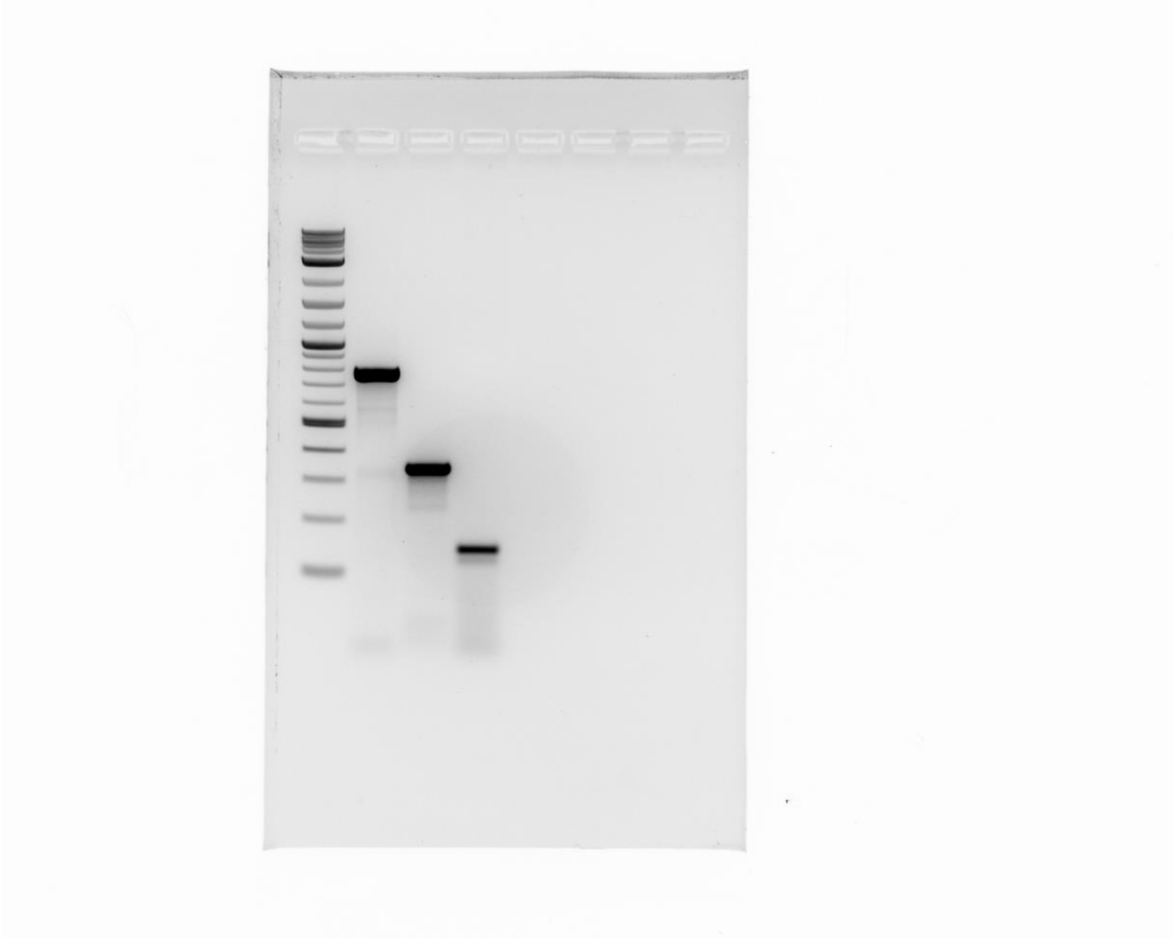

Full unedited gel with marked bands used in the figure

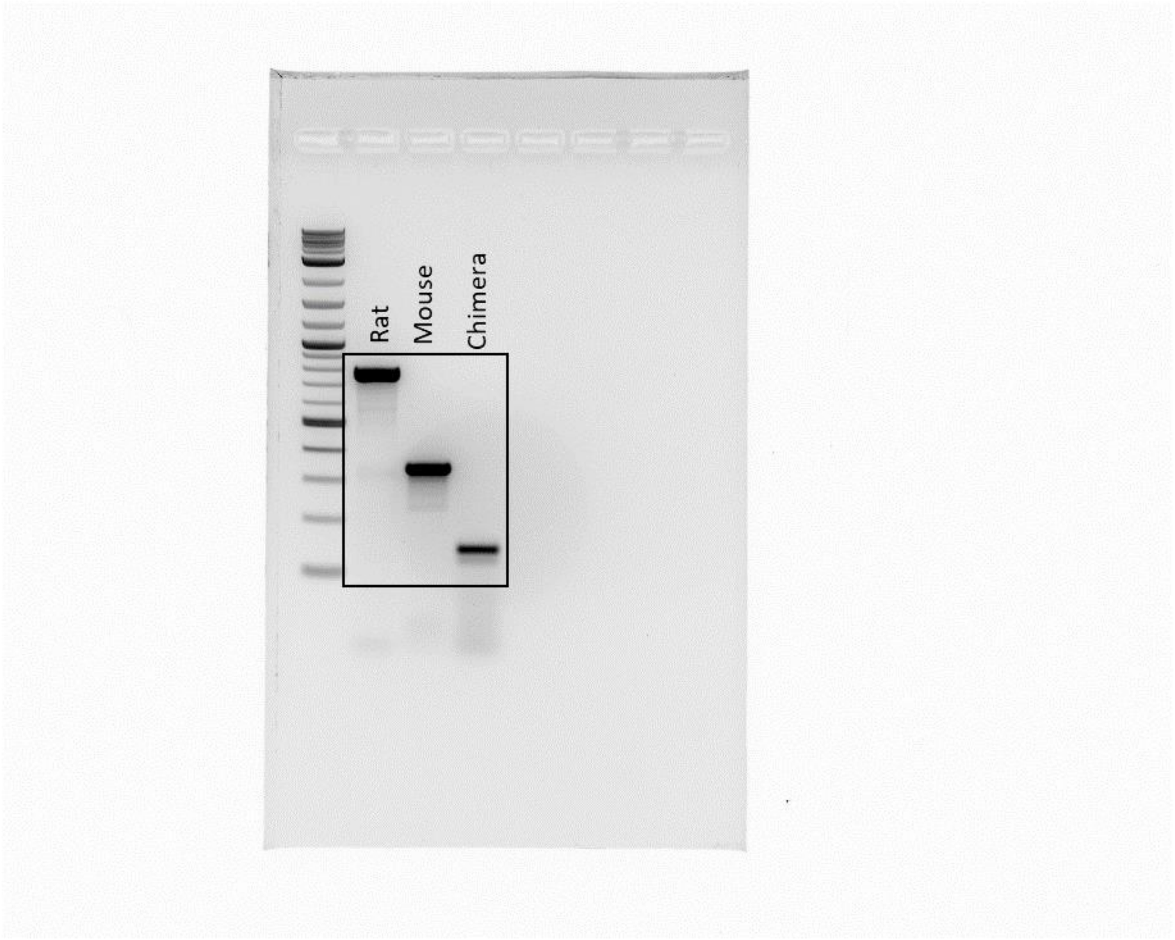

Supplement: Unedited blot and gel images [file jci-134-166998-s253.pdf]
